# Synthesis of Novel 2-(Pyridin-2-yl) Pyrimidine Derivatives and Study of Their Anti-Fibrosis Activity

**DOI:** 10.3390/molecules25225226

**Published:** 2020-11-10

**Authors:** Yi-Fei Gu, Yue Zhang, Feng-li Yue, Shao-tong Li, Zhuo-qi Zhang, Jing Li, Xu Bai

**Affiliations:** 1 The Center for Combinatorial Chemistry and Drug Discovery of Jilin University, The School of Pharmaceutical Sciences, Jilin University, 1266 Fujin Road, Changchun 130021, China; guyifei2007@163.com (Y.-F.G.); zy2932886799@outlook.com (Y.Z.); zqzhang@jlu.edu.cn (Z.-q.Z.); 2 Department of Pharmacology, College of Basic Medical Sciences, Jilin University, Changchun 130021, China; yuefl18@mails.jlu.edu.cn (F.-l.Y.); gaby9905@163.com (S.-t.L.)

**Keywords:** anti-fibrosis, collagen prolyl 4-hydroxylases, pyrimidine, COL1A1, synthesis

## Abstract

A pyrimidine moiety exhibiting a wide range of pharmacological activities has been employed in the design of privileged structures in medicinal chemistry. To prepare libraries of novel heterocyclic compounds with potential biological activities, a series of novel 2-(pyridin-2-yl) pyrimidine derivatives were designed, synthesized and their biological activities were evaluated against immortalized rat hepatic stellate cells (HSC-T6). Fourteen compounds were found to present better anti-fibrotic activities than Pirfenidone and Bipy55′DC. Among them, compounds ethyl 6-(5-(*p*-tolylcarbamoyl)pyrimidin-2-yl)nicotinate (**12m**) and ethyl 6-(5-((3,4-difluorophenyl)carbamoyl)pyrimidin-2-yl)nicotinate (**12q**) show the best activities with IC_50_ values of 45.69 μM and 45.81 μM, respectively. Furthermore, the study of anti-fibrosis activity was evaluated by Picro-Sirius red staining, hydroxyproline assay and ELISA detection of Collagen type I alpha 1 (COL1A1) protein expression. Our study showed that compounds **12m** and **12q** effectively inhibited the expression of collagen, and the content of hydroxyproline in cell culture medium in vitro, indicating that compounds **12m** and **12q** might be developed the novel anti-fibrotic drugs.

## 1. Introduction

Construction of novel heterocyclic compound libraries with potential biological activities is an important component of medicinal chemistry and chemical biology [1]. The pyrimidine moiety has been considered as a privileged structure in medicinal chemistry [2] and the compounds containing pyrimidine as the core are reported to exhibit diverse types of biological and pharmaceutical activities. For example, pyrimidine derivatives are known as antimicrobial [3,4,5], antiviral [6,7], antitumor [8,9,10] and antifibrotic compounds [11].

**Figure 1:**
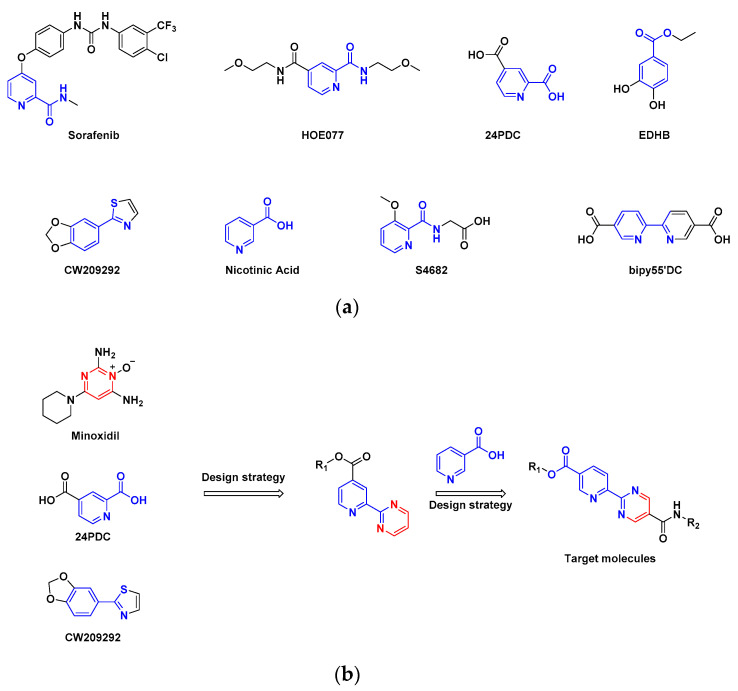
(**a**) Examples of small molecules with similarly active structure fragments; (**b**) design strategy.

The discovery of anti-fibrotic drugs has attracted great attention from organic and medicinal chemists. As shown in Figure 1a, vascular endothelial growth factor receptor 2 (VEGFR-2) and Platelet derived growth factor-β (PDGF-β) inhibitor sorafinib reduced collagen deposition by nearly 63% in a liver fibrosis model [12]; N^2^,N^4^-bis(2-methoxyethyl)pyridine-2,4-dicarboxamide (HOE-077), which is a prodrug of pyridine-2,4-dicarboxylic acid (24PDC), can inhibit collagen synthesis in rat and dog models by inactivating hepatic stellate cells that are mainly responsible for collagen synthesis in liver fibrosis [13,14]; Ethyl 3,4-dihydroxybenzoate and S4682 were effective in inhibiting collagen synthesis in keloid fibroblasts or the CCl_4_ model of chronic hepatic injury, by inhibition of collagen prolyl-4-hydroxylase (CP4H) [14,15]; 2-(benzo[d][1,3]dioxol-5-yl)thiazole (CW209292) displays anti-fibrotic activity in rats with dimethylnitrosamine (DMN)-induced hepatic fibrosis by blocking the mRNA expression of transforming growth factor-β1 (TGF-β1) in hepatic stellate cells [16]; nicotinic acid prevented fibrosis by its antioxidant properties and reducing the expression of TGF-β in thioacetamide (TAA)-induced hepatic fibrogenesis [17]. All of those small molecules contain the similarly structure fragment.

CP4Hs have been considered an important target for treating fibrotic disease and a few small molecular inhibitors have been reported in the literature [18,19]. However, most of these compounds usually have poor activities in cultured cells [20]. It is our ongoing interest to discover novel compounds with potent anti-fibrotic activities [21,22,23]. In this report, the novel compounds containing pyrimidine scaffold as the core were designed by combining pyrimidine ring with the similarly structure fragment through a molecular hybridization strategy, as shown in Figure 1b. Herein, we report the preparation of pyrimidine–pyridine compounds, CP4H activity in cultured cells and their anti-fibrotic activities against immortalized rat hepatic stellate cells (HSC-T6).

## 2. Results

### 2.1. Chemistry

**Scheme 1:**
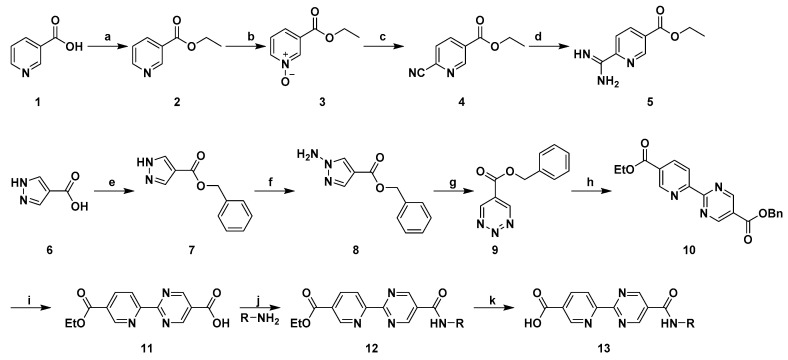
Synthetic route of compound 12 and 13. Reagent and conditions: (**a**) H_2_SO_4_, EtOH, reflux, 8 h; (**b**) 3-chloroperbenzoic acid, Dichloromethane (DCM), rt, 24 h; (**c**) TMSCN, Triethylamine (TEA), Acetonitrile (ACN), reflux, 10 h; (**d**) Na, EtOH, NH4Cl, reflux, 28 h; (**e**) BnOH, EDCI, HOBT, DCM, rt, 24 h; (**f**) amino 4-nitrobenzoate, NMP, rt, 8 h; (**g**) NaIO_4_, DCM, H_2_O, 2 h; (**h**) compound 5, K_2_CO_3_, Dioxane, rt, 8 h; (**i**) Pd/C, H_2_, MeOH, rt, 24 h; (**j**) EDCI, HOBT, DMF, rt, 30 h; (**k**) NaOH, EtOH, rt, 4 h.

In this work, the key intermediate **5** was obtained by a convenient four-step procedure in moderate yields and the synthetic route was depicted in Scheme 1: First, esterification of nicotinic acid was undertaken to yield **2**; then, compound **2** was oxidated with 3-chloroperoxybenzoic acid (mCPBA) to afford pyridine *N*-Oxides **3**; next, a nucleophilic substitution of the ortho-position of pyridine *N*-Oxides **3** with trimethylsilyl cyanide (TMSCN) was conducted to generate **4** [24]; finally, compound **4** was reacted with Na and NH_4_Cl in EtOH solution to get compound **5** [25].

The target compounds (**12a**–**12t** and **13a**–**13t**) were prepared according to Scheme 1, starting from the commercially available 4-pyrazolecarboxylic acid. Briefly, condensation of 4-pyrazolecarboxylic acid **6** with benzyl alcohol in the presence of 1-ethyl-3(3-dimethylpropylamine) carbodiimide (EDCI) and 1-hydroxybenzotriazole (HOBT) afforded compound **7** [26]. Intermediate **8** was prepared by using amino 4-nitrobenzoate in 1-methyl-2-pyrrolidinone (NMP). This step was followed by oxidation of **8** with NaIO_4_ at a low temperature [27]. Subsequently, a Diels–Alder reaction between the key intermediate **5** and Benzyl 1,2,3-triazine-5-carboxylate (**9**) led to the formation of the correspondent compound **10** [28], which in turn was converted to the carboxylic acid intermediate **11** by reaction with hydrogen at room temperature. Finally, EDCI and HOBT mediated an amide coupling reaction between the activated intermediate carboxylic acid **11** and various substituted amines formed the desired compounds **12a**–**12t** in moderate to good yields and compounds **12a**–**12t** were also converted into the desired product compounds **13a**–**13t** by hydrolysis reactions.

### 2.2. Biology

#### 2.2.1. MTT Assay on HSC-T6 Cell Proliferation

To evaluate the anti-proliferation activities of target compounds, HSC-T6 cells (Rat Hepatic Stellate Cell) were employed since they have been recognized as an effective and convenient method for anti-proliferation screening model in vitro [29,30]. In our experiment, HSC-T6 cells were seeded to the 96-well plate and were cultured with serum-free medium. This process resulted in HSC-T6 cells transforming into a static period and synchronizing, then the culture medium was refreshed with 2% fetal bovine serum to stimulate the activation of HSC-T6 cells. The inhibitory activities of all synthesized compounds were evaluated by 3-(4,5-dimethylthiazol-2-yl)-2,5-diphenyltetrazolium bromide (MTT) assay with Bipy55′DC, 24PDC and clinical drug Pirfenidone (PFD) as control (Bipy55′DC and 24PDC are typical CP4H inhibitors; Pirfenidone (PFD) can treat idiopathic pulmonary fibrosis with good tolerance and little side effects [31]). It showed that the cell growth increased in speed after cells were cultured with 2% fetal bovine serum in the control group. Moreover, the floating cell is rare in the 100 μM group when co-culturing with the **12m** and **12q** compounds for about 48 h, indicating that the death cell is rare, but we can observe that the hepatic stellate cells become slim. The half-maximal inhibitory concentration (IC_50_) values of compounds are shown in Table 1.

From the screening results in Table 1, some of the target compounds displayed better anti-fibrosis activity than Pirfenidone (PFD), Bipy55′DC and 24PDC on HSC-T6 cells. Regarding the activity of the tested compounds, compounds (**12k**, **12l**, **12m**, **12n**, **12o**, **12p** and **12q**) with ethyl groups on the substituent group R exhibited better activity effectiveness than compounds (**13k**, **13l**, **13m**, **13n**, **13o**, **13p** and **13q**) with hydrogen atoms on the substituent group R. In addition, compounds (**12k**, **12l**, **12m**, **12n**, **12o**, **12q** and **12r**) with phenyl rings on the substituent group R_1_ presented more highly inhibitory activities than those (**12b**, **12c**, **12d**, **12f**, **12e**, **12i** and **12j**) with benzyl groups on the substituent group R_1_. Similarly, compounds (**12l**, **12m**) with the 4-substituent group R_1_ showed better activity compared to compounds (**12n**, **12p**) with the 3-substituent group R_1_. According to a series of chemical structural modifications, we got two highly active compounds: **12m** and **12q**. To summarize what has been mentioned above, the results encouraged us to further investigate the anti-fibrotic activity of the two compounds **12m** and **12q**.

#### 2.2.2. **12m** and **12q** Screened for Prolyl-4-hydroxylase Content In Vitro

**Figure 2:**
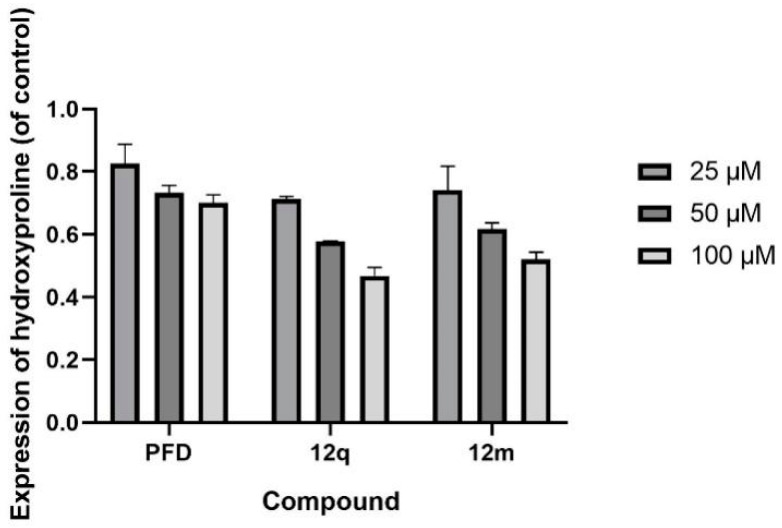
The effect of **PFD**, **12q** and **12m** on the expression of hydroxyproline in HSC-T6 cell line.

Initial screening for prolyl-4-hydroxylase inhibitory activity was performed by determining the hydroxyproline content in HSC-T6 cells [20] and collagen was also determined by estimating the content of hydroxyproline [32]. As shown in Figure 2, the content of hydroxyproline was significantly reduced in the presence of **12m** and **12q** at a concentration of 50 μM or 100 μM. The result of the hydroxyproline assay displayed that **12m** and **12q** are potent inhibitors of collagen prolyl-4-hydroxylase. The result also suggested that **12m** and **12q** do have a potential effect on suppressing the production of collagen in vitro.

#### 2.2.3. **12m** and **12q** Suppressed the Total Collagen Accumulation In Vitro

**Figure 3:**
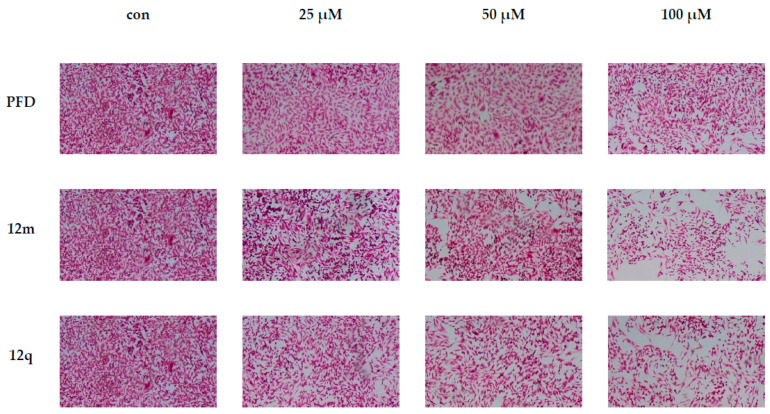
Picro-Sirius red (PSR) staining for total collagen accumulation in HSC-T6 cells.

Extracellular collagen deposition was established by Picro-Sirius red (PSR) staining to test inhibitory activity of **12m** and **12q** against total collagen accumulation [33,34]. As depicted in Figure 3, treatment with **12m** and **12q** reduced the cell density and inhibited the total collagen accumulation in a dose-dependent manner. In addition, it is worth noting that the total collagen accumulation was significantly decreased in the presence of **12m** and **12q** at a concentration of 100 μM.

#### 2.2.4. **12m** and **12q** Suppressed the Protein Expression of COL1A1 In Vitro

**Figure 4:**
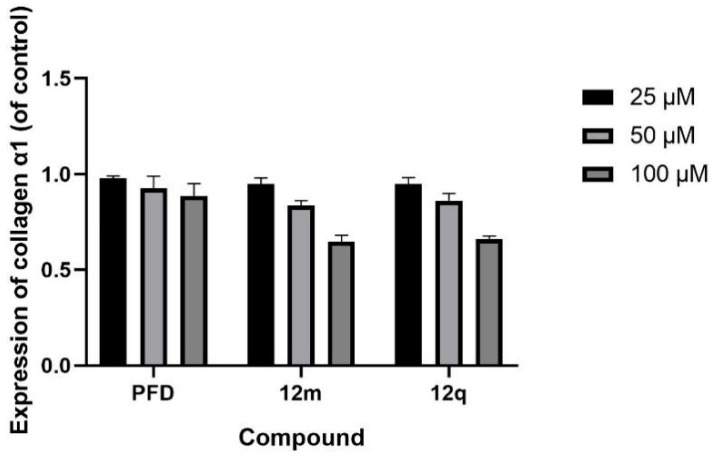
The effect of **PFD**, **12q** and **12m** on the expression of collagen α1 in HSC-T6 cell line.

Collagen type I alpha 1 (COL1A1), which has been generally recognized as a fibrotic marker, is overexpressed in the common fibrotic diseases. To further study the anti-fibrotic activity of compounds **12m** and **12q**, their abilities of inhibiting protein expression of COL1A1 in vitro were investigated through the ELISA method. As displayed in Figure 4, it was observed that **12m** could reduce the expression of COL1A1 in HSC-T6 cells, which means that the extracellular matrix molecules’ deposition was also reduced.

## 3. Experimental Section

### 3.1. General Information

All commercial reagents were used as received without additional purification. The melting point was uncorrected. Mass spectra were obtained using the electro spray ionization (ESI) method. The ^1^H and ^13^C Nuclear Magnetic Resonance (NMR) data were obtained on a 300 MHz NMR spectrometer (Bruker, Rheinstetten, Germany) with tetramethylsilane (TMS) as the internal standard, using Chloroform-d (CDCl_3_), DMSO-d_6_ as solvents. All the NMR spectra can be found in Supplementary Materials (Figures S1–S101). Multiplicities are indicated as follows: s, singlet; d, doublet; t, triplet; q, quartet; m, multiplet; dd, doubled doublet; coupling constants is in Hertz (Hz); chemicalshift (δ) is in parts per million (ppm).

### 3.2. Synthesis of Compounds

Ethyl nicotinate (**2**): ethanol (50 mL) and nicotinic acid 1 (2.0 g, 16.24 mmol) was added in a 100 mL round bottomed flask. To this, a catalytic amount of concentrated H_2_SO_4_ was added at room temperature and was heated under reflux with continuous stirring for 8 h at 85 °C. After 8 h, the reaction was monitored by TLC using an ethyl acetate and hexane (1:4) system. Stirring was continued until TLC indicated the completion of reaction. Then, the reaction mixture was allowed to reach room temperature. Ethanol was distilled off under reduced pressure and the residue was dissolved in water and then extracted twice with ethyl acetate. The combined organic solutions were washed with saturated NaHCO_3_ solution, and finally washed with water and dried over anhydrous Na_2_SO_4_. The solvent was removed under vacuum to get ethyl nicotinate (2.4 g, 98%): ^1^H NMR (300 MHz, DMSO-*d*_6_) δ 9.07 (dd, *J* = 2.2, 0.8 Hz, 1H), 8.80 (dd, *J* = 4.8, 1.7 Hz, 1H), 8.33–8.21 (m, 1H), 7.55 (ddd, *J* = 8.0, 4.8, 0.9 Hz, 1H), 4.34 (q, *J* = 7.1 Hz, 2H), 1.32 (t, *J* = 7.1 Hz, 3H). ^13^C NMR (75 MHz, DMSO-*d*_6_) δ 164.64, 153.56, 149.90, 136.73, 125.73, 123.84, 61.11, 14.00.

*3-(ethoxycarbonyl)pyridine 1-oxide* (**3**): A solution of ethyl nicotinate 2 (2.0 g, 14.6 mmol) in DCM (150 mL) was added m-CPBA (6.5 g, 29.2 mmol) at 0 °C. After stirring at 0 °C for 1 h and then at room temperature overnight, the mixture was poured into ice water. Then, 2N NaOH was added to adjust the pH to 8–9 and the resultant mixture was extracted with DCM (3 × 200 mL). The organic layer was dried over Na_2_SO_4_, filtered and concentrated. The crude product was purified by flash column chromatography on silica gel (2% *v/v* MeOH in DCM) to afford the title compound (2.19 g, 98%) as a white solid; melting point: 101–102 °C; ^1^H NMR (300 MHz, CDCl_3_) δ 8.82–8.75 (m, 1H), 8.34 (ddd, *J* = 6.5, 1.8, 1.1 Hz, 1H), 7.91–7.82 (m, 1H), 7.37 (dd, *J* = 7.8, 6.6 Hz, 1H), 4.43 (q, *J* = 7.1 Hz, 2H), 1.41 (t, *J* = 7.1 Hz, 3H). ^13^C NMR (75 MHz, DMSO-*d*_6_) δ 162.70, 142.45, 138.78, 129.60, 126.83, 125.19, 61.84, 13.89.

*Ethyl 6-cyanonicotinate* (**4**): To a solution of 3-(ethoxycarbonyl)pyridine *N*-oxide (2.19 g, 14.3 mmol) in acetonitrile (ACN) (30 mL) was added trimethylsilyl cyanide (7.2 mL, 57.2 mmol) and triethylamine (TEA) (3.74 mL, 28.6 mmol). The solution was heated at reflux for 10 h. The reaction was then concentrated and diluted with DCM and water before adding 1N HCl. The mixture was extracted with DCM and the organic solutions were combined and washed with brine, dried over Na_2_SO_4_, filtered and concentrated in vacuo. The crude product was purified by flash column chromatography on silica gel (2% *v/v* MeOH in DCM) to afford the title compound (1.26 g, 50%) as a white solid: melting point: 56–57 °C; ^1^H NMR (300 MHz, DMSO-*d*_6_) δ 9.19 (dd, *J* = 2.1, 0.9 Hz, 1H), 8.51 (dd, *J* = 8.1, 2.1 Hz, 1H), 8.21 (dd, *J* = 8.1, 0.9 Hz, 1H), 4.39 (q, *J* = 7.1 Hz, 2H), 1.35 (t, *J* = 7.1 Hz, 3H). ^13^C NMR (75 MHz, CDCl_3_) δ 163.56, 151.72, 137.95, 136.83, 128.82, 127.94, 116.50, 62.21, 14.10.

*Ethyl 6-carbamimidoylnicotinate hydrochloride* (**5**): A solution of sodium metal (8 mg, 0.35 mmol) in EtOH (20 mL) was added to a solution of ethyl 6-cyanonicotinate (1.26 g, 7.15 mmol) in EtOH (20 mL) and the mixture was stirred for 24 h at room temperature. Ammonium chloride (574 mg, 10.7 mmol) was added and the mixture was stirred at 50 °C for 24 h. The resulting suspension was filtered and the solvent removed under reduced pressure. The solid obtained was washed with ethyl ether (3 × 20 mL) to give compound 5 (982 mg, 60%) as a yellow solid: ^1^H NMR (300 MHz, DMSO-*d*_6_) δ 9.53 (s, 3H), 9.22 (dd, *J* = 2.0, 0.7 Hz, 1H), 8.62 (dd, *J* = 8.3, 2.1 Hz, 1H), 8.47 (d, *J* = 8.3 Hz, 1H), 4.40 (q, *J* = 7.1 Hz, 2H), 1.36 (t, *J* = 7.1 Hz, 3H). ^13^C NMR (75 MHz, DMSO-*d*_6_) δ 163.63, 161.60, 149.61, 147.36, 138.84, 129.31, 123.57, 61.77, 13.97. MS(ESI): *m*/*z* 194.20 [M + H]^+^.

*Benzyl 1H-pyrazole-4-carboxylate* (**7**): EDCI (2.6 g, 13.38 mmol) and HOBT (1.8 g, 13.38 mmol) were added at 0 °C. to a solution of 1H-pyrazole-4-carboxylic acid (1 g, 8.92 mmol) and benzyl alcohol (1.39 mL, 13.38 mmol) in dichloromethane followed by stirring at room temperature for 24 h. The reaction mixture was extracted three times with EtOAc (20 mL). The combined organic layers were washed with brine, dried (Na_2_SO_4_), filtered and concentrated in vacuo. The crude product was purified by flash column chromatography on silica gel (33% *v/v* ethyl acetate in Petroleum ether (60–90 °C) to afford the title compound (514 mg, 23%) as a white solid: melting point: 130–131 °C; ^1^H NMR (300 MHz, CDCl_3_) δ 8.09 (s, 2H), 7.46–7.33 (m, 5H), 5.31 (s, 2H). ^13^C NMR (75 MHz, DMSO-*d*_6_) δ 162.51, 136.51, 128.43, 127.90, 127.77, 113.21, 64.94. 

*Benzyl 1-amino-1H-pyrazole-4-carboxylate* (**8**): A solution of potassium tert-butoxide (KOBu-t) (264 mg, 2.35 mmol) in NMP (20 mL) was added to a stirring solution of 7 (300 mg, 2.14 mmol) in NMP (30 mL). The resulting light pink solution was stirred at room temperature for 20 min. After 20 min, a solution of O-(4-nitrobenzoyl)hydroxylamine(449 mg, 2.46 mmol) in NMP (10 mL) was added to the reaction mixture. The reaction mixture was stirred at room temperature for 8 h. After 8 h, the reaction mixture was treated with saturated aqueous NaCl (7%, 60 mL) and ethyl acetate (60 mL). The organic layer was separated and the aqueous layer was extracted with ethyl acetate (60 mL). The combined organic layers were washed with aqueous sodium bicarbonate (5%, 45 mL), H_2_O (45 mL), dried (Na_2_SO_4_), and concentrated on a rotary evaporator. The crude product was purified by flash chromatography on silica gel (20% *v/v* ethyl acetate in Petroleum ether (60–90°C) to afford the title compound (160 mg, 50%) as a yellow oil: ^1^H NMR (300 MHz, DMSO-*d*_6_) δ 8.11 (d, *J* = 0.7 Hz, 1H), 7.81 (d, *J* = 0.9 Hz, 1H), 7.49–7.30 (m, 5H), 6.75 (s, 2H), 5.28 (s, 2H). ^13^C NMR (75 MHz, DMSO-*d*_6_) δ 162.01, 137.58, 136.45, 131.74, 128.44, 127.91, 127.79, 127.72, 111.76, 64.96. MS(ESI): *m*/*z* 218.32 [M + H]^+^.

*Benzyl 1,2,3-triazine-5-carboxylate* (**9**): Compound **8** (400 mg, 1.84 mmol) was taken up in CH_2_Cl_2_ (30 mL) and cooled to 0 °C. NaIO_4_ (787 mg, 3.68 mmol) in H_2_O (15 mL), which was cooled to 0 °C and added to the stirring solution of **8** at 0 °C. The reaction mixture was warmed to room temperature and stirred for 2 h. After 2 h, the organic layer was separated and the aqueous layer was extracted with CH_2_Cl_2_ (20 mL × 3). The combined organic layers were washed with saturated aqueous NaCl (30 mL), dried (Na_2_SO_4_) and concentrated on a rotary evaporator. The crude product was purified by flash chromatography on silica gel (20% *v/v* ethyl acetate in Petroleum ether (60–90 °C) to afford the title compound (272 mg, 81%) as a white solid: melting point: 47–48 °C; ^1^H NMR (300 MHz, CDCl_3_) δ 9.48 (s, 2H), 7.48–7.39 (m, 5H), 5.47 (s, 2H). ^13^C NMR (75 MHz, CDCl_3_) δ 162.12, 147.86, 133.99, 129.14, 128.85, 128.77, 119.21, 77.41, 76.98, 76.56, 68.75. MS(ESI): *m*/*z* 216.30 [M + H]^+^.

*Benzyl 2-(5-(ethoxycarbonyl)pyridin-2-yl)pyrimidine-5-carboxylate* (**10**): benzyl 1,2,3-triazine-5-carboxylate (80 mg, 0.5 mmol) was added to a stirring solution of ethyl 6-carbamimidoylnicotinate hydrochloride (100 mg, 0.5 mmol) and K_2_CO_3_ (139 mg, 1 mmol) in CH_3_CN (20 mL) followed by stirring at room temperature for 8 h. The reaction mixture was extracted three times with EtOAc (20 mL). The combined organic layers were washed with brine, dried (Na_2_SO_4_), filtered and concentrated in vacuo. The crude product was purified by flash column chromatography on silica gel (2% *v/v* MeOH in DCM) to afford the title compound (127 mg, 70%) as a white solid: ^1^H NMR (300 MHz, CDCl_3_) δ 9.47 (d, *J* = 0.6 Hz, 2H), 9.45–9.40 (m, 1H), 8.68 (dd, *J* = 8.2, 0.7 Hz, 1H), 8.49 (dd, *J* = 8.3, 2.1 Hz, 1H), 7.51–7.37 (m, 5H), 5.46 (s, 2H), 4.46 (q, *J* = 7.1 Hz, 2H), 1.44 (t, *J* = 7.1 Hz, 3H). ^13^C NMR (75 MHz, CDCl_3_) δ 165.12, 164.72, 163.28, 158.84, 156.68, 151.18, 138.11, 134.93, 128.67, 128.65, 128.39, 127.63, 123.90, 123.15, 67.54, 61.60, 14.15. MS(ESI): *m*/*z* 364.29 [M + H]^+^.

*2-(5-(ethoxycarbonyl)pyridin-2-yl)pyrimidine-5-carboxylic acid* (**11**): a solution of benzyl 2-(5-(ethoxycarbonyl)pyridin-2-yl)pyrimidine-5-carboxylate (5.7 g, 15.69 mmol) in MeOH (150 mL) was added to palladium on carbon (200 mg, 0.18 mmol) and the reaction mixture was subjected to an atmosphere of hydrogen for 24 h. The catalyst was removed via filtration over Celite and the solvent removed in vacuo. The crude product was purified by flash column chromatography on silica gel (2% *v/v* MeOH in DCM) to afford the title compound (2.7 g, Yield 73%) as a yellow solid: melting point: 231–232 °C; ^1^H NMR (300 MHz, DMSO-*d*_6_) δ 13.91 (s, 1H), 9.40 (s, 2H), 9.27 (d, *J* = 1.4 Hz, 1H), 8.61 (d, *J* = 8.3 Hz, 1H), 8.51 (dd, *J* = 8.2, 2.2 Hz, 1H), 4.41 (q, *J* = 7.1 Hz, 2H), 1.38 (t, *J* = 7.1 Hz, 3H). MS(ESI): *m*/*z* 275.25 [M + H]^+^.

*Ethyl 6-(5-((4-fluorobenzyl)carbamoyl)pyrimidin-2-yl)nicotinate* (**12a**): A solution of 2-(5-(ethoxycarbonyl)pyridin-2-yl)pyrimidine-5-carboxylic acid (200 mg, 0.731 mmol) in DMF (2 mL) and DCM (6 mL) at 0 °C was treated with HOBT (114 mg, 0.841 mmol), and EDCI (162 mg, 0.841 mmol). The mixture was stirred at 0 °C for 1 h. Then, 4-Fluorobenzylamine (167 μL, 1.463 mmol) and triethylamine (150 μL, 0.407 mmol) were added and the mixture was warmed to room temperature for 30 h. The reaction mixture was extracted three times with EtOAc (15 mL). The combined organic layers were washed with brine, dried (MgSO_4_), filtered and concentrated in vacuo. The crude product was purified by flash column chromatography on silica gel (1.5% *v/v* MeOH in DCM) to afford the title compound (163 mg, 58%) as a yellow solid, melting point: 225–226 °C, ^1^H NMR (300 MHz, DMSO-*d*_6_) δ 9.50 (t, *J* = 5.8 Hz, 1H), 9.39 (s, 2H), 9.26 (dd, *J* = 2.2, 0.9 Hz, 1H), 8.59 (dd, *J* = 8.3, 0.9 Hz, 1H), 8.50 (dd, *J* = 8.3, 2.2 Hz, 1H), 7.50–7.32 (m, 2H), 7.25–7.11 (m, 2H), 4.54 (d, *J* = 5.9 Hz, 2H), 4.40 (q, *J* = 7.1 Hz, 2H), 1.37 (t, *J* = 7.1 Hz, 3H). ^13^C NMR (75 MHz, DMSO-*d*_6_) δ 164.33, 163.32, 162.84, 162.74, 156.95, 156.80, 150.13, 137.98, 134.95, 129.45, 129.35, 126.69, 126.54, 123.79, 115.17, 114.89, 61.35, 42.00, 14.01. MS(ESI): *m*/*z* 381.38 [M + H]^+^.

*6-(5-((4-fluorobenzyl)carbamoyl)pyrimidin-2-yl)nicotinic acid* (**13a**): ethyl ethyl 6-(5-((4-fluorobenzyl)carbamoyl)pyrimidin-2-yl)nicotinate (100 mg, 0.26 mmol) was taken up in methanol (12 mL) to which NaOH 1 mol·L^−1^ (5 mL) was added and the solution was stirred at room temperature for about 5 h. The solvent was evaporated and the residue dissolved in a minimum amount of water. The solution was extracted with dichloromethane (2 × 15 mL) and the aqueous layer acidified to pH~3 with concentrated HCl. The product precipitated as an almost white solid. After filtration and washing with water, the solid was dried (67 mg, 72%): melting point: 294–295 °C; ^1^H NMR (300 MHz, DMSO-*d*_6_) δ 13.61 (s, 1H), 9.56 (t, *J* = 5.8 Hz, 1H), 9.41 (s, 2H), 9.24 (s, 1H), 8.56 (d, *J* = 8.2 Hz, 1H), 8.46 (dd, *J* = 8.2, 2.0 Hz,1H), 7.42–7.30 (m, 4H), 7.27 (dt, *J* = 9.5, 4.1 Hz, 1H), 4.55 (d, *J* = 5.8 Hz, 2H). ^13^C NMR (75 MHz, DMSO-*d*_6_) δ 165.93, 163.48, 162.79, 156.89, 156.73, 150.48, 138.86, 138.21, 128.36, 127.42, 126.96, 126.62, 126.57, 123.80, 42.71. MS(ESI): *m*/*z* 353.41 [M + H]^+^.

*Ethyl 6-(5-((4-chlorobenzyl)carbamoyl)pyrimidin-2-yl)nicotinate* (**12b**): Yellow solid, melting point: 224–225 °C, Yield: 63%, ^1^H NMR (300 MHz, DMSO-*d*_6_) δ 9.52 (t, *J* = 5.9 Hz, 1H), 9.39 (s, 2H), 9.26 (dd, *J* = 2.2, 0.8 Hz, 1H), 8.59 (dd, *J* = 8.3, 0.8 Hz, 1H), 8.49 (dd, *J* = 8.3, 2.2 Hz, 1H), 7.41 (s, 4H), 4.54 (d, *J* = 5.9 Hz, 2H), 4.40 (q, *J* = 7.1 Hz,2H), 1.37 (t, *J* = 7.1 Hz, 3H). ^13^C NMR (75 MHz, DMSO-*d*_6_) δ 164.32, 163.33, 162.79, 156.92, 156.82, 150.14, 138.00, 137.84, 131.49, 129.25, 128.25, 126.69, 126.48, 123.81, 61.36, 42.03, 14.02. MS(ESI): *m*/*z* 397.33 [M + H]^+^.

*6-(5-((4-chlorobenzyl)carbamoyl)pyrimidin-2-yl)nicotinic acid* (**13b**): White solid, melting point: 306–307 °C, Yield: 78%, ^1^H NMR (300 MHz, DMSO-*d*_6_) δ 13.65 (s, 1H), 9.57 (t, *J* = 5.9 Hz, 1H), 9.41 (s, 2H), 9.25 (s, 1H), 8.58 (d, *J* = 8.3 Hz,1H), 8.48 (dd, *J* = 8.2, 2.1 Hz, 1H), 7.42 (s, 4H), 4.54 (d, *J* = 5.9 Hz, 2H). ^13^C NMR (75 MHz, DMSO-*d*_6_) δ 165.90, 163.47, 162.83, 156.91, 156.69, 150.48, 138.20, 137.95, 131.50, 129.31, 128.27, 127.68, 126.49, 123.78, 42.05. MS(ESI): *m*/*z* 369.32 [M + H]^+^.

*Ethyl 6-(5-((4-bromobenzyl)carbamoyl)pyrimidin-2-yl)nicotinate* (**12c**): Yellow solid, melting point: 227–228 °C, Yield: 69%, ^1^H NMR (300 MHz, DMSO-*d*_6_) δ 9.52 (t, *J* = 5.9 Hz, 1H), 9.39 (s, 2H), 9.26 (dd, *J* = 2.2, 0.8 Hz, 1H), 8.61–8.57 (m, 1H), 8.50 (dd, *J* = 8.2, 2.2 Hz, 1H), 7.57–7.52 (m, 2H), 7.35 (d, *J* = 8.5 Hz, 2H), 4.52 (d, *J* = 5.9 Hz, 2H), 4.40 (q, *J* = 7.0 Hz, 2H), 1.37 (t, *J* = 7.1 Hz, 3H). ^13^C NMR (75 MHz, DMSO-*d*_6_) δ 164.32, 163.35, 162.79, 156.93, 156.80, 150.13, 138.27, 137.96, 131.16, 129.62, 126.69, 126.48, 123.79, 119.96, 61.35, 42.11, 14.02. MS(ESI): m/z 441.29 [M + H]^+^.

*6-(5-((4-bromobenzyl)carbamoyl)pyrimidin-2-yl)nicotinic acid* (**13c**): White solid, melting point: 314–315 °C, Yield: 81%, ^1^H NMR (300 MHz, DMSO-*d*_6_) δ 13.64 (s, 1H), 9.52 (t, *J* = 5.9 Hz, 1H), 9.39 (s, 2H), 9.25 (s, 1H), 8.57 (d, *J* = 8.4 Hz, 1H), 8.47 (dd, *J* = 8.2, 2.1 Hz, 1H), 7.60–7.49 (m, 2H), 7.35 (d, *J* = 8.4 Hz, 2H), 4.52 (d, *J* = 5.9 Hz, 2H). ^13^C NMR (75 MHz, DMSO-*d*_6_) δ 165.92, 163.53, 162.88, 156.86, 156.74, 150.47, 138.34, 138.18, 131.21, 129.67, 127.64, 126.50, 123.80, 120.00, 42.14. MS(ESI): m/z 413.33 [M + H]^+^.

*Ethyl 6-(5-((4-methylbenzyl)carbamoyl)pyrimidin-2-yl)nicotinate* (**12d**): Yellow solid, melting point: 210–211 °C, Yield: 70%, ^1^H NMR (300 MHz, DMSO-*d*_6_) δ 9.45 (t, *J* = 5.9 Hz, 1H), 9.39 (s, 2H), 9.26 (dd, *J* = 2.2, 0.9 Hz, 1H), 8.59 (dd, *J* = 8.3, 0.9 Hz, 1H), 8.50 (dd, *J* = 8.3, 2.2 Hz, 1H), 7.27 (d, *J* = 8.0 Hz, 2H), 7.16 (d, *J* = 7.8 Hz, 2H), 4.51 (d, *J* = 5.8 Hz, 2H), 4.40 (q, *J* = 7.1 Hz, 2H), 2.28 (s, 3H), 1.37 (t, *J* = 7.1 Hz, 3H). ^13^C NMR (75 MHz, DMSO-*d*_6_) δ 164.37, 163.36, 162.67, 157.04, 156.78, 150.14, 137.98, 136.02, 135.74, 128.85, 127.39, 126.73, 126.71, 123.80, 61.35, 42.48, 20.60, 14.03. MS(ESI): m/z 377.39 [M + H]^+^.

*6-(5-((4-methylbenzyl)carbamoyl)pyrimidin-2-yl)nicotinic acid (***13d**): White solid, melting point: 295–296 °C, Yield: 76%, ^1^H NMR (300 MHz, DMSO-*d*_6_) δ 13.64 (s, 1H), 9.46 (t, *J* = 5.9 Hz, 1H), 9.39 (s, 2H), 9.24 (d, *J* = 1.4 Hz, 1H), 8.57 (d, *J* = 8.3 Hz, 1H), 8.47 (dd, *J* = 8.2, 2.1 Hz, 1H), 7.26 (d, *J* = 8.0 Hz, 2H), 7.16 (d, *J* = 7.8 Hz, 2H), 4.50 (d, *J* = 5.8 Hz, 2H), 2.28 (s, 3H). ^13^C NMR (75 MHz, DMSO-*d*_6_) δ 165.92, 163.46, 162.70, 156.84, 156.75, 150.47, 138.18, 136.03, 135.80, 128.88, 127.62, 127.43, 126.64, 123.77, 42.48, 20.65. MS(ESI): *m*/*z* 349.42 [M + H]^+^.

*Ethyl 6-(5-((4-methoxybenzyl)carbamoyl)pyrimidin-2-yl)nicotinate* (**12e**): Yellow solid, melting point: 218¨C219 °C, Yield: 76%, ^1^H NMR (300 MHz, DMSO-*d*_6_) δ 9.43 (t, *J* = 6.0 Hz, 1H), 9.38 (s,2H), 9.26 (dd, *J* = 2.2, 0.9 Hz, 1H), 8.59 (dd, *J* = 8.3, 0.9 Hz, 1H), 8.50 (dd, *J* = 8.3, 2.2 Hz, 1H), 7.31 (d, *J* = 8.8 Hz, 2H), 6.97–6.86 (m, 2H), 4.48 (d, *J* = 5.8 Hz, 2H), 4.40 (q, *J* = 7.1 Hz, 2H), 3.74 (s, 3H), 1.37 (t, *J* = 7.1 Hz, 3H). ^13^C NMR (75 MHz, DMSO-*d*_6_) δ 164.37, 163.34, 162.61, 158.35, 157.04, 156.78, 150.14, 137.99, 130.71, 128.79, 128.68, 126.73, 123.81, 113.76, 61.35, 55.05, 42.21, 14.03. MS(ESI): *m*/*z* 393.43 [M + H]^+^.

*6-(5-((4-methoxybenzyl)carbamoyl)pyrimidin-2-yl)nicotinic acid* (**13e**): Grey solid, melting point: 296–297 °C, Yield: 65%, ^1^H NMR (300 MHz, DMSO-*d*_6_) δ 13.63 (s, 1H), 9.44 (t, *J* = 5.8 Hz, 1H), 9.38 (s, 2H), 9.24 (s, 1H), 8.57 (d, *J* = 8.3 Hz, 1H), 8.47 (dd, *J* = 8.2, 2.1 Hz, 1H), 7.42–7.21 (m, 2H), 7.01–6.85 (m, 2H), 4.48 (d, *J* = 5.7 Hz, 2H), 3.73 (s, 3H). ^13^C NMR (75 MHz, DMSO-*d*_6_) δ 165.89, 163.49, 162.66, 158.38, 156.82, 150.44, 138.16, 130.79, 128.84, 127.63, 126.71, 123.77, 113.78, 55.08, 42.24. MS(ESI): *m*/*z* 365.05 [M + H]^+^.

*Ethyl 6-(5-((3-bromobenzyl)carbamoyl)pyrimidin-2-yl)nicotinate* (**12f**): Yellow solid, melting point: 209–210 °C, Yield: 70%, ^1^H NMR (300 MHz, DMSO-*d*_6_) δ 9.52 (t, *J* = 6.0 Hz, 1H), 9.40 (s, 2H), 9.26 (dd, *J* = 2.2, 0.9 Hz, 1H), 8.59 (dd, *J* = 8.3, 0.9 Hz, 1H), 8.50 (dd, *J* = 8.3, 2.2 Hz, 1H), 7.59 (s, 1H), 7.51–7.44 (m, 1H), 7.40 (d, *J* = 7.9 Hz, 1H), 7.32 (t, *J* = 7.7 Hz, 1H), 4.56 (d, *J* = 5.8 Hz, 2H), 4.40 (q, *J* = 7.1 Hz, 2H), 1.37 (t, *J* = 7.1 Hz, 3H). ^13^C NMR (75 MHz, DMSO-*d*_6_) δ 164.36, 163.38, 162.86, 156.97, 156.89, 150.19, 141.68, 138.04, 130.56, 130.17, 129.85, 126.73, 126.52, 126.46, 123.85, 121.67, 61.40, 42.19, 14.07. MS(ESI): *m*/*z* 441.26 [M + H]^+^.

*6-(5-((3-bromobenzyl)carbamoyl)pyrimidin-2-yl)nicotinic acid* (**13f**): White solid, melting point: 270–271 °C, Yield: 71%, ^1^H NMR (300 MHz, DMSO-*d*_6_) δ 13.64 (s, 1H), 9.56 (t, *J* = 5.9 Hz, 1H), 9.41 (s, 2H), 9.25 (s, 1H), 8.57 (d, *J* = 8.2 Hz, 1H), 8.47 (dd, *J* = 8.2, 2.1 Hz, 1H), 7.59 (t, *J* = 1.6 Hz, 1H), 7.50–7.46 (m, 1H), 7.40 (dd, *J* = 6.3, 1.5 Hz, 1H), 7.32 (t, *J* = 7.7 Hz, 1H), 4.55 (d, *J* = 5.8 Hz, 2H). ^13^C NMR (75 MHz, DMSO-*d*_6_) δ 165.89, 163.54, 162.89, 156.91, 156.73, 150.46, 141.75, 138.17, 130.52, 130.19, 129.82, 127.70, 126.54, 126.47, 123.78, 121.63, 42.20. MS(ESI): *m*/*z* 413.32 [M + H]^+^.

*Ethyl 6-(5-((3-methylbenzyl)carbamoyl)pyrimidin-2-yl)nicotinate* (**12g**): White solid, melting point: 180–181 °C, Yield: 70%, ^1^H NMR (300 MHz, DMSO-*d*_6_) δ 9.46 (t, *J* = 5.9 Hz, 1H), 9.40 (s, 2H), 9.26 (dd, *J* = 2.2, 0.9 Hz, 1H), 8.59 (dd, *J* = 8.3, 0.9 Hz,1H), 8.49 (dd, *J* = 8.3, 2.2 Hz, 1H), 7.28–7.14 (m, 3H), 7.08 (d, *J* = 7.4 Hz, 1H), 4.52 (d, *J* = 5.8 Hz, 2H), 4.40 (q, *J* = 7.1 Hz, 2H), 2.30 (s, 3H), 1.37 (t, *J* = 7.1 Hz, 3H). ^13^C NMR (75 MHz, DMSO-*d*_6_) δ 164.34, 163.30, 162.66, 156.96, 156.84, 150.15, 138.69, 137.98, 137.44, 128.25, 128.05, 127.59, 126.68, 126.60, 124.54, 123.80, 61.38, 42.71, 20.97, 14.04. MS(ESI): *m*/*z* 377.37 [M + H]^+^.

*6-(5-((3-methylbenzyl)carbamoyl)pyrimidin-2-yl)nicotinic acid* (**13g**): White solid, melting point: 276–277 °C, Yield: 81%, ^1^H NMR (300 MHz, DMSO-*d*_6_) δ 13.64 (s, 1H), 9.48 (t, *J* = 5.9 Hz, 1H), 9.40 (s, 2H), 9.25 (s, 1H), 8.57 (d, *J* = 8.3 Hz, 1H), 8.47 (dd, *J* = 8.2, 2.1 Hz, 1H), 7.24 (t, *J* = 7.4 Hz, 1H), 7.22–7.12 (m, 2H), 7.08 (d, *J* = 7.3 Hz, 1H), 4.52 (d, *J* = 5.8 Hz, 2H), 2.30 (s, 3H). ^13^C NMR (75 MHz, DMSO-*d*_6_) δ 165.93, 163.45, 162.73, 156.89, 156.75, 150.48, 138.75, 138.21, 137.45, 128.28, 128.06, 127.60, 126.62, 124.56, 123.80, 42.71, 21.01. MS(ESI): *m*/*z* 349.42 [M + H]^+^.

*Ethyl 6-(5-((2-fluorobenzyl)carbamoyl)pyrimidin-2-yl)nicotinate* (**12h**): Yellow solid, melting point: 235–236 °C, Yield: 70%, ^1^H NMR (300 MHz, DMSO-*d*_6_) δ 9.49 (t, *J* = 5.8 Hz, 1H), 9.40 (s, 2H), 9.26 (dd, *J* = 2.2, 0.9 Hz, 1H), 8.59 (dd, *J* = 8.3, 0.9 Hz,1H), 8.50 (dd, *J* = 8.3, 2.2 Hz, 1H), 7.47 (dd, *J* = 8.6, 6.7 Hz, 1H), 7.40–7.29 (m, 1H), 7.27–7.16 (m, 1H), 4.59 (d, *J* = 5.7 Hz, 2H), 4.40 (q, *J* = 7.1 Hz, 2H), 1.37 (t, *J* = 7.1 Hz, 3H). ^13^C NMR (75 MHz, DMSO-*d*_6_) δ 164.40, 163.44, 162.92, 161.75, 158.50, 157.04, 156.87, 150.18, 138.02, 129.89, 129.84, 129.18, 129.08, 126.79, 126.55, 125.45, 125.25, 124.39, 123.85, 115.27, 114.99, 61.39, 36.68, 14.05. MS(ESI): *m*/*z* 381.38 [M + H]^+^.

*6-(5-((2-fluorobenzyl)carbamoyl)pyrimidin-2-yl)nicotinic acid* (**13h**): White solid, melting point: 280–281 °C, Yield: 77%, ^1^H NMR (300 MHz, DMSO-*d*_6_) δ 13.64 (s, 1H), 9.51 (t, *J* = 5.7 Hz, 1H), 9.40 (s, 2H), 9.24 (s, 1H), 8.57 (d, *J* = 8.2 Hz, 1H), 8.47 (dd, *J* = 8.2, 2.1 Hz, 1H), 7.47 (td, *J* = 7.5, 1.6 Hz, 1H), 7.40–7.31 (m, 1H), 7.26–7.16 (m, 2H), 4.59 (d, *J* = 5.6 Hz, 2H). ^13^C NMR (75 MHz, DMSO-*d*_6_) δ 165.90, 163.54, 162.96, 161.75, 158.50, 156.89, 156.77, 150.47, 138.17, 129.89, 129.83, 129.16, 129.06, 127.66, 126.51, 125.49, 125.30, 124.40, 124.36, 123.79, 115.26, 114.98, 36.65, 36.59. MS(ESI): *m*/*z* 353.41 [M + H]^+^.

*Ethyl 6-(5-((2,3-difluorobenzyl)carbamoyl)pyrimidin-2-yl)nicotinate* (**12i**): White solid, melting point: 181–182 °C, Yield: 66%, ^1^H NMR (300 MHz, DMSO-*d*_6_) δ 9.52 (t, *J* = 6.0 Hz, 1H), 9.40 (s, 2H), 9.26 (dd, *J* = 2.2, 0.9 Hz, 1H), 8.59 (dd, *J* = 8.3, 0.9 Hz, 1H), 8.50 (dd, *J* = 8.3, 2.2 Hz, 1H), 7.51–7.34 (m, 2H), 7.24 (s, 1H), 4.54 (d, *J* = 5.7 Hz, 2H), 4.40 (q, *J* = 7.1 Hz, 2H), 1.37 (t, *J* = 7.1 Hz, 3H). ^13^C NMR (75 MHz, DMSO-*d*_6_) δ 164.37, 163.38, 162.93, 156.97, 156.89, 150.98, 150.82, 150.18, 150.00, 146.93, 146.77, 145.96, 144.97, 138.03, 136.72, 136.68, 136.65, 136.61, 126.73, 126.49, 124.16, 124.12, 124.07, 124.03, 123.84, 117.42, 117.20, 116.53, 116.30, 61.39, 41.78, 14.05. MS(ESI): *m*/*z* 399.37 [M + H]^+^.

*6-(5-((2,3-difluorobenzyl)carbamoyl)pyrimidin-2-yl)nicotinic acid* (**13i**): White solid, melting point: 283–284 °C, Yield: 54%, ^1^H NMR (300 MHz, DMSO-*d*_6_) δ 13.64 (s, 1H), 9.54 (t, *J* = 5.9 Hz, 1H), 9.40 (s, 2H), 9.25 (s, 1H), 8.57 (d, *J* = 8.1 Hz, 1H), 8.47 (dd, *J* = 8.2, 2.1 Hz, 1H), 7.52–7.32 (m, 2H), 7.31–7.15 (m, 1H), 4.54 (d, *J* = 5.8 Hz, 2H). ^13^C NMR (75 MHz, DMSO-*d*_6_) δ 165.92, 163.52, 162.96, 156.90, 156.74, 150.98, 150.81, 150.47, 150.16, 150.00, 147.72, 147.55, 146.92, 146.75, 138.19, 136.70, 127.64, 126.46, 124.16, 124.12, 124.08, 124.03, 123.79, 117.41, 117.19, 116.54, 116.31, 41.78. MS(ESI): *m*/z 371.48 [M + H]^+^.

*Ethyl 6-(5-((3-fluorobenzyl)carbamoyl)pyrimidin-2-yl)nicotinate* (**12j**): Yellow solid, melting point: 209–210 °C, Yield: 67%, ^1^H NMR (300 MHz, DMSO-*d*_6_) δ 9.52 (t, *J* = 6.0 Hz, 1H), 9.41 (s, 2H), 9.26 (dd, *J* = 2.2, 0.9 Hz, 1H), 8.60 (dd, *J* = 8.3, 0.8 Hz, 1H), 8.50 (dd, *J* = 8.3, 2.2 Hz, 1H), 7.46–7.35 (m, 1H), 7.27–7.17 (m, 2H), 7.11 (t, *J* = 9.1 Hz, 1H), 4.57 (d, *J* = 5.8 Hz, 2H), 4.40 (q, *J* = 7.1 Hz, 2H), 1.37 (t, *J* = 7.1 Hz, 3H). ^13^C NMR (75 MHz, DMSO-*d*_6_) δ 164.37, 163.38, 162.91, 156.99, 156.90, 150.19, 141.78, 138.05, 130.36, 130.25, 126.73, 126.53, 123.85, 123.37, 123.34, 114.22, 113.94, 113.87, 113.59, 61.40, 42.23, 14.06. MS(ESI): *m*/*z* 381.35 [M + H]^+^.

*6-(5-((3-fluorobenzyl)carbamoyl)pyrimidin-2-yl)nicotinic acid* (**13j**): White solid, melting point: 280–281 °C, Yield: 59%, ^1^H NMR (300 MHz, DMSO-*d*_6_) δ 13.64 (s, 1H), 9.56 (t, *J* = 5.9 Hz, 1H), 9.41 (s, 2H), 9.25 (s, 1H), 8.58 (d, *J* = 8.1 Hz, 1H), 8.47 (dd, *J* = 8.2, 2.1 Hz, 1H), 7.46–7.35 (m, 1H), 7.26–7.17 (m, 2H), 7.11 (ddd, *J* = 10.7, 5.3, 1.4 Hz, 1H), 4.57 (d, *J* = 5.9 Hz, 2H). ^13^C NMR (75 MHz, DMSO-*d*_6_) δ 165.93, 163.86, 162.94, 160.63, 156.95, 156.73, 150.49, 141.94, 141.85, 138.23, 130.35, 130.24, 126.52, 123.82, 123.40, 123.37, 114.25, 113.97, 113.86, 113.58, 42.24. MS(ESI): *m*/*z* 353.40 [M + H]^+^.

*Ethyl 6-(5-((4-chlorophenyl)carbamoyl)pyrimidin-2-yl)nicotinate* (**12k**): Yellow solid, melting point: 249–250 °C, Yield: 50%, ^1^H NMR (300 MHz, DMSO-*d*_6_) δ 10.80 (s, 1H), 9.46 (s, 2H), 9.28 (dd, *J* = 2.2, 0.8 Hz, 1H), 8.62 (dd, *J* = 8.3, 0.8 Hz, 1H), 8.51 (dd, *J* = 8.3, 2.2 Hz, 1H), 7.82 (d, *J* = 8.9 Hz, 2H), 7.54–7.39 (m, 2H), 4.41 (q, *J* = 7.1 Hz, 2H), 1.38 (t, *J* = 7.1 Hz, 3H). ^13^C NMR (75 MHz, DMSO-*d*_6_) δ 164.43, 163.54, 162.07, 157.20, 157.03, 150.26, 138.10, 137.49, 128.74, 127.99, 127.43, 126.89, 123.96, 121.97, 61.43, 14.09. MS(ESI): *m*/*z* 383.33 [M + H]^+^.

*6-(5-((4-chlorophenyl)carbamoyl)pyrimidin-2-yl)nicotinic acid* (**13k**): Grey solid, melting point: 319–320 °C, Yield: 71%, ^1^H NMR (300 MHz, DMSO-*d*_6_) δ 13.50 (s, 1H), 10.87 (s, 1H), 9.46 (s, 2H), 9.25 (s, 1H), 8.57 (d, *J* = 7.9 Hz, 1H), 8.47 (dd, *J* = 8.2, 1.7 Hz, 1H), 7.83 (d, *J* = 8.9 Hz, 2H), 7.44 (d, *J* = 8.8 Hz, 2H). ^13^C NMR (75 MHz, DMSO-*d*_6_) δ 165.93, 163.62, 162.03, 157.17, 156.63, 150.50, 138.14, 137.51, 128.66, 127.93, 127.56, 127.28, 123.83, 121.96. MS(ESI): *m*/*z* 355.39 [M + H]^+^.

*Ethyl 6-(5-((4-bromophenyl)carbamoyl)pyrimidin-2-yl)nicotinate* (**12l**): Yellow solid, melting point: 269–270 °C, Yield: 58%, ^1^H NMR (300 MHz, DMSO-*d*_6_) δ 10.81 (s, 1H), 9.46 (s, 2H), 9.28 (dd, *J* = 2.2, 0.8 Hz, 1H), 8.62 (dd, *J* = 8.3, 0.8 Hz, 1H), 8.52 (dd, *J* = 8.3, 2.2 Hz, 1H), 7.85–7.72 (m, 2H), 7.69–7.53 (m, 2H), 4.41 (q, *J* = 7.1 Hz, 2H), 1.38 (t, *J* = 7.1 Hz, 3H). ^13^C NMR (75 MHz, DMSO-*d*_6_) δ 164.41, 163.54, 162.07, 157.19, 157.01, 150.25, 138.09, 137.90, 131.65, 127.43, 126.87, 123.94, 122.30, 116.06, 61.42, 14.08. MS(ESI): *m*/*z* 427.34 [M + H]^+^.

*6-(5-((4-bromophenyl)carbamoyl)pyrimidin-2-yl)nicotinic acid* (**13l**): Yellow solid, melting point: 320–321 °C, Yield: 86%, ^1^H NMR (300 MHz, DMSO-*d*_6_) δ 10.93 (s, 1H), 9.48 (s, 2H), 9.25 (s, 1H), 8.59 (d, *J* = 8.1 Hz, 1H), 8.48 (dd, *J* = 8.2, 2.0 Hz, 1H), 7.80 (d, *J* = 8.9 Hz, 2H), 7.57 (d, *J* = 8.8 Hz, 2H). ^13^C NMR (75 MHz, DMSO-*d*_6_) δ 165.82, 163.49, 162.02, 157.21, 156.54, 150.37, 138.34, 137.95, 131.56, 127.74, 127.30, 123.89, 122.33, 116.02. MS(ESI): *m*/*z* 339.36 [M + H]^+^.

*Ethyl 6-(5-(p-tolylcarbamoyl)pyrimidin-2-yl)nicotinate* (**12m**): Yellow solid, melting point: 272–273 °C, Yield: 71%, ^1^H NMR (300 MHz, DMSO-*d*_6_) δ 10.61 (s, 1H), 9.45 (s, 2H), 9.27 (dd, *J* = 2.2, 0.8 Hz, 1H), 8.61 (dd, *J* = 8.3, 0.8 Hz, 1H), 8.51 (dd, *J* = 8.3, 2.2 Hz, 1H), 7.67 (d, *J* = 8.4 Hz, 2H), 7.21 (d, *J* = 8.3 Hz, 2H), 4.40 (q, *J* = 7.1 Hz, 2H), 2.30 (s, 3H), 1.37 (t, *J* = 7.1 Hz, 3H). ^13^C NMR (75 MHz, DMSO-*d*_6_) δ 164.35, 163.29, 161.57, 157.06, 156.96, 150.20, 138.02, 135.94, 133.31, 129.13, 127.51, 126.73, 123.84, 120.36, 61.38, 20.49, 14.04. MS(ESI): *m*/*z* 363.37 [M + H]^+^.

*6-(5-(p-tolylcarbamoyl)pyrimidin-2-yl)nicotinic acid* (**13m**): Yellow solid, melting point: 330–331 °C, Yield: 76%, ^1^H NMR (300 MHz, DMSO-*d*_6_) δ 10.69 (s, 1H), 9.46 (s, 2H), 9.25 (s, 1H), 8.59 (d, *J* = 8.3 Hz, 1H), 8.48 (d, *J* = 7.6 Hz, 1H), 7.68 (d, *J* = 7.6 Hz, 2H), 7.19 (d, *J* = 7.4 Hz, 2H), 2.29 (s, 3H). ^13^C NMR (75 MHz, DMSO-*d*_6_) δ 165.86, 163.40, 161.65, 157.10, 156.68, 150.42, 138.27, 136.00, 133.33, 129.11, 127.69, 127.54, 123.82, 120.46, 20.50. MS(ESI): *m*/*z* 335.37 [M + H]^+^.

*Ethyl 6-(5-((3-bromophenyl)carbamoyl)pyrimidin-2-yl)nicotinate* (**12n**): White solid, melting point: 216–217 °C, Yield: 50%, ^1^H NMR (300 MHz, DMSO-*d*_6_) δ 10.82 (s,1H), 9.46 (s, 2H), 9.28 (d, *J* = 1.4 Hz, 1H), 8.62 (d, *J* = 8.2 Hz, 1H), 8.52 (dd, *J* = 8.3, 2.2 Hz, 1H), 8.11 (d, *J* = 1.7 Hz,1H), 7.81–7.70 (m, 1H), 7.47–7.29 (m, 2H), 4.41 (q, *J* = 7.1 Hz, 2H), 1.38 (t, *J* = 7.1 Hz, 3H). ^13^C NMR (75 MHz, DMSO-*d*_6_) δ 164.44, 163.62, 162.24, 157.29, 157.24, 150.28, 140.11, 138.12, 130.84, 127.01, 126.95, 123.99, 122.75, 121.49, 119.91, 119.17, 61.44, 14.10. MS(ESI): *m*/*z* 427.33 [M + H]^+^.

*6-(5-((3-bromophenyl)carbamoyl)pyrimidin-2-yl)nicotinic acid* (**13n**): White solid, melting point: 310–311 °C, Yield: 54%, ^1^H NMR (300 MHz, DMSO-*d*_6_) δ 13.64 (s, 1H), 10.98 (s, 1H), 9.49 (s, 2H), 9.26 (s, 1H), 8.58 (s, 1H), 8.49 (s, 1H), 8.13 (s, 1H), 7.80 (s, 1H), 7.35 (s, 2H). ^13^C NMR (75 MHz, DMSO-*d*6) δ 165.90, 163.64, 162.19, 157.28, 156.62, 150.51, 140.16, 138.23, 130.74, 127.84, 127.15, 126.84, 123.88, 122.70, 121.44, 119.13. MS(ESI): *m*/*z* 399.41 [M + H]^+^.

*Ethyl 6-(5-((4-methoxyphenyl)carbamoyl)pyrimidin-2-yl)nicotinate* (**12o**): Yellow solid, melting point: 269–270 °C, Yield: 70%, ^1^H NMR (300 MHz, DMSO-*d*_6_) δ 10.57 (s, 1H), 9.45 (s, 2H), 9.27 (dd, *J* = 2.2, 0.8 Hz, 1H), 8.62 (dd, *J* = 8.3, 0.8 Hz, 1H), 8.51 (dd, *J* = 8.3, 2.2 Hz, 1H), 7.75–7.64 (m, 2H), 7.02–6.93 (m, 2H), 4.41 (q, *J* = 7.1 Hz, 2H), 3.76 (s, 3H), 1.38 (t, *J* = 7.1 Hz, 3H). ^13^C NMR (75 MHz, DMSO-*d*_6_) δ 164.41, 163.36, 161.38, 157.06, 157.02, 156.00, 150.22, 138.04, 131.51, 127.58, 126.80, 123.86, 122.04, 113.92, 61.39, 55.21, 14.07. MS(ESI): *m*/*z* 379.35 [M + H]^+^.

*6-(5-((4-methoxyphenyl)carbamoyl)pyrimidin-2-yl)nicotinic acid* (**13o**): Grey solid, melting point: 285–286 °C, Yield: 65%, ^1^H NMR (300 MHz, DMSO-*d*_6_) δ 10.72 (s, 1H), 9.49 (s, 2H), 9.27 (s, 1H), 8.61 (d, *J* = 7.6 Hz, 1H), 8.49 (dd, *J* = 8.2, 1.8 Hz, 1H), 7.74 (d, *J* = 9.0 Hz, 2H), 7.04–6.92 (m, 2H), 3.77 (s, 3H). ^13^C NMR (75 MHz, DMSO-*d*_6_) δ 165.86, 163.34, 161.38, 157.08, 156.70, 155.97, 150.43, 138.24, 131.59, 127.74, 127.52, 123.81, 122.07, 113.87, 55.21. MS(ESI): *m*/*z* 351.35 [M + H]^+^.

*Ethyl 6-(5-(m-tolylcarbamoyl)pyrimidin-2-yl)nicotinate* (**12p**): Yellow solid, melting point: 204–205 °C, Yield: 60%, ^1^H NMR (300 MHz, DMSO-*d*_6_) δ 10.60 (s, 1H), 9.45 (s, 2H), 9.27 (d, *J* = 1.4 Hz, 1H), 8.61 (d, *J* = 8.2 Hz, 1H), 8.51 (dd, *J* = 8.2, 2.1 Hz, 1H), 7.59 (d, *J* = 9.1 Hz, 2H), 7.28 (t, *J* = 7.6 Hz, 1H), 6.99 (d, *J* = 7.6 Hz, 1H), 4.41 (q, *J* = 7.1 Hz, 2H), 2.33 (s, 3H), 1.38 (t, *J* = 7.1 Hz, 3H). ^13^C NMR (75 MHz, DMSO-*d*_6_) δ 164.37, 163.35, 161.76, 157.10, 156.97, 150.21, 138.37, 138.05, 137.96, 128.59, 127.54, 126.77, 124.99, 123.87, 120.89, 117.56, 61.39, 21.15, 14.05. MS(ESI): m/z 363.35 [M + H]^+^.

*6-(5-(m-tolylcarbamoyl)pyrimidin-2-yl)nicotinic acid* (**13p**): White solid, melting point: 311–312 °C, Yield: 60%, ^1^H NMR (300 MHz, DMSO-*d*_6_) δ 13.67 (s, 1H), 10.61 (s, 1H), 9.46 (s, 2H), 9.28 (s, 1H), 8.60 (d, *J* = 7.9 Hz, 1H), 8.49 (dd, *J* = 8.2, 1.9 Hz, 1H), 7.61 (d, *J* = 9.2 Hz, 2H), 7.29 (t, *J* = 7.8 Hz, 1H), 6.99 (d, *J* = 7.6 Hz, 1H), 2.34 (s, 3H). ^13^C NMR (75 MHz, DMSO-*d*_6_) δ 165.94, 163.52, 161.82, 157.11, 156.73, 150.51, 138.41, 138.20, 137.97, 128.61, 127.65, 127.51, 124.99, 123.84, 120.89, 117.56, 21.17. MS(ESI): *m*/*z* 355.42 [M + H]^+^.

*Ethyl 6-(5-((3,4-difluorophenyl)carbamoyl)pyrimidin-2-yl)nicotinate* (**12q**): Yellow solid, melting point: 256–257 °C, Yield: 61%, ^1^H NMR (300 MHz, DMSO-*d*_6_) δ 10.88 (s, 1H), 9.45 (s, 2H), 9.27 (dd, *J* = 2.2, 0.9 Hz, 1H), 8.61 (dd, *J* = 8.3, 0.9 Hz, 1H), 8.50 (dd, *J* = 8.3, 2.2 Hz, 1H), 7.94 (ddd, *J* = 9.9, 7.3, 2.0 Hz, 1H), 7.59–7.41 (m, 2H), 4.40 (q, *J* = 7.1 Hz, 2H), 1.37 (t, *J* = 7.1 Hz, 3H). ^13^C NMR (75 MHz, DMSO-*d*_6_) δ 164.38, 163.57, 162.11, 157.20, 156.92, 150.25, 138.10, 135.46, 127.18, 126.85, 123.96, 117.69, 117.45, 116.86, 109.55, 109.27, 61.42, 14.07. MS(ESI): m/z 385.35 [M + H]^+^.

*6-(5-((3,4-difluorophenyl)carbamoyl)pyrimidin-2-yl)nicotinic acid* (**13q**): White solid, melting point: 304–305 °C, Yield: 70%, ^1^H NMR (300 MHz, DMSO-*d*_6_) δ 13.63 (s, 1H), 10.90 (s, 1H), 9.46 (s, 2H), 9.26 (s, 1H), 8.57 (s, 1H), 8.48 (dd, *J* = 8.2, 1.8 Hz, 1H), 7.95 (ddd, *J* = 13.1, 7.5, 2.4 Hz, 1H), 7.63–7.36 (m, 2H). ^13^C NMR (75 MHz, DMSO-*d*_6_) δ 165.90, 163.82, 162.07, 157.17, 156.61, 150.49, 147.54, 147.31, 147.14, 144.32, 144.16, 138.18, 135.58, 135.54, 135.46, 135.42, 127.63, 127.08, 123.86, 117.62, 117.39, 116.81, 116.77, 116.73, 116.69, 109.52, 109.23. MS(ESI): *m*/*z* 357.42 [M + H]^+^.

*Ethyl 6-(5-((3-fluorophenyl)carbamoyl)pyrimidin-2-yl)nicotinate* (**12r**): White solid, melting point: 239–240 °C, Yield: 65%, ^1^H NMR (300 MHz, DMSO-*d*_6_) δ 10.87 (s, 1H), 9.46 (s, 2H), 9.32–9.24 (m, 1H), 8.62 (d, *J* = 8.3 Hz, 1H), 8.52 (dd, *J* = 8.3, 2.2 Hz, 1H), 7.76 (d, *J* = 11.4 Hz, 1H), 7.56 (d, *J* = 9.1 Hz, 1H), 7.45 (dd, *J* = 15.0, 8.1 Hz, 1H), 7.02 (dd, *J* = 11.3, 5.6 Hz, 1H), 4.41 (q, *J* = 7.1 Hz, 2H), 1.38 (t, *J* = 7.1 Hz, 3H). ^13^C NMR (75 MHz, DMSO-*d*_6_) δ 164.32, 163.60, 163.45, 162.11, 160.40, 157.16, 156.86, 150.20, 140.26, 140.11, 138.01, 130.47, 130.35, 127.21, 126.77, 123.88, 116.03, 110.89, 110.61, 107.22, 106.87, 61.38, 14.03. MS(ESI): *m*/*z* 367.33 [M + H]^+^.

*6-(5-((3-fluorophenyl)carbamoyl)pyrimidin-2-yl)nicotinic acid* (**13r**): Light green solid, melting point: 296–297 °C, Yield: 76%, ^1^H NMR (300 MHz, DMSO-*d*_6_) δ 13.64 (s, 1H), 10.92 (s, 1H), 9.48 (s, 2H), 9.27 (s, 1H), 8.60 (d, *J* = 7.7 Hz, 1H), 8.49 (dd, *J* = 8.2, 1.8 Hz, 1H), 7.78 (dt, *J* = 11.6, 2.2 Hz, 1H), 7.65–7.56 (m, 1H), 7.44 (td, *J* = 8.2, 6.9 Hz, 1H), 7.00 (td, *J* = 8.3, 1.8 Hz, 1H). ^13^C NMR (75 MHz, DMSO-*d*_6_) δ 165.88, 163.63, 162.22, 160.43, 157.19, 156.69, 150.48, 140.32, 140.17, 138.18, 130.46, 130.33, 127.70, 127.26, 123.86, 116.13, 116.09, 110.90, 110.62, 107.32, 106.97. MS(ESI): *m*/*z* 339.45 [M + H]^+^.

*Ethyl 6-(5-((2-fluorophenyl)carbamoyl)pyrimidin-2-yl)nicotinate* (**12s**): Yellow solid, melting point: 189–190 °C, Yield: 62%, ^1^H NMR (300 MHz, DMSO-*d*_6_) δ 10.65 (s, 1H), 9.47 (s, 2H), 9.28 (dd, *J* = 2.2, 0.9 Hz, 1H), 8.63 (dd, *J* = 8.3, 0.9 Hz, 1H), 8.52 (dd, *J* = 8.3, 2.2 Hz, 1H), 7.73 (dt, *J* = 7.9, 4.1 Hz, 1H), 7.47–7.17 (m, 3H), 4.41 (q, *J* = 7.1 Hz, 2H), 1.38 (t, *J* = 7.1 Hz, 3H). ^13^C NMR (75 MHz, DMSO-*d*_6_) δ 164.37, 163.58, 162.14, 157.27, 156.94, 153.69, 150.22, 138.07, 127.33, 127.23, 126.81, 126.74, 126.56, 125.07, 124.90, 124.48, 124.43, 123.94, 116.06, 115.80, 61.41, 14.06. MS(ESI): *m*/*z* 367.35 [M + H]^+^.

*6-(5-((2-fluorophenyl)carbamoyl)pyrimidin-2-yl)nicotinic acid* (**13s**): White solid, melting point: 285–286 °C, Yield: 42%, ^1^H NMR (300 MHz, DMSO-*d*_6_) δ 13.68 (s, 1H), 10.81 (s, 1H), 9.52 (s, 2H), 9.28 (s, 1H), 8.61 (d, *J* = 8.2 Hz, 1H), 8.50 (dd, *J* = 8.2, 2.1 Hz, 1H), 7.73 (td, *J* = 7.9, 1.6 Hz, 1H), 7.43–7.20 (m, 3H). ^13^C NMR (75 MHz, DMSO-*d*_6_) δ 165.89, 163.64, 162.12, 157.35, 157.10, 156.60, 153.82, 150.52, 138.26, 127.78, 127.36, 127.26, 126.72, 125.08, 124.92, 124.45, 124.40, 123.88, 116.06, 115.80. MS(ESI): *m*/*z* 339.26 [M + H]^+^.

*Ethyl 2-(5-(ethoxycarbonyl)pyridin-2-yl)pyrimidine-5-carboxylate* (**12t**): Yellow solid, melting point: 214–215 °C, Yield: 75%, ^1^H NMR (300 MHz, DMSO-d6) δ 9.34 (s, 2H), 9.26 (dd, *J* = 2.2, 0.9 Hz, 1H), 8.95 (t, *J* = 5.4 Hz, 1H), 8.58 (dd, *J* = 8.3, 0.9 Hz, 1H), 8.49 (dd, *J* = 8.3, 2.2 Hz, 1H), 4.40 (q, *J* = 7.1 Hz, 2H), 3.46–3.25 (m, 2H), 1.37 (t, *J* = 7.1 Hz, 3H), 1.18 (t, *J* = 7.2 Hz, 3H). ^13^C NMR (75 MHz, DMSO-d6) δ 164.38, 163.23, 162.40, 157.05, 156.66, 150.15, 137.96, 126.87, 126.71, 123.77, 61.37, 34.19, 14.51, 14.05. MS(ESI): *m*/*z* 301.32 [M + H]^+^.

*6-(5-(ethoxycarbonyl)pyrimidin-2-yl)nicotinic acid* (**13t**): White solid, melting point: 284–285 °C, Yield: 66%, ^1^H NMR (300 MHz, DMSO-*d*_6_) δ 13.61 (s, 1H), 9.35 (s, 2H), 9.24 (s, 1H), 8.99 (t, *J* = 5.4 Hz, 1H), 8.56 (d, *J* = 8.2 Hz, 1H), 8.47 (dd, *J* = 8.2, 2.1 Hz, 1H), 3.48–3.19 (m, 2H), 1.18 (t, *J* = 7.2 Hz, 3H). ^13^C NMR (75 MHz, DMSO-*d*_6_) δ 165.89, 163.28, 162.39, 156.70, 156.57, 150.43, 138.19, 127.56, 126.79, 123.73, 34.17, 14.53. MS(ESI): *m*/*z* 273.28 [M + H]^+^.

*Ethyl 6-(5-((4-fluorophenyl)carbamoyl)pyrimidin-2-yl)nicotinate* (**12u**): White solid, melting point: 198–199 °C, Yield: 72%, ^1^H NMR (300 MHz, DMSO-d6) δ 10.74 (s,1H), 9.46 (s, 2H), 9.28 (dd, *J* = 2.1, 0.8 Hz, 1H), 8.62 (dd, *J* = 8.3, 0.8 Hz, 1H), 8.51 (dd, *J* = 8.3, 2.2 Hz, 1H), 7.81 (dd, *J* = 5.7, 3.5 Hz, 2H), 7.33–7.19 (m, 2H), 4.41 (q, *J* = 7.1 Hz, 2H), 1.38 (t, *J* = 7.1 Hz, 3H). ^13^C NMR (75 MHz, DMSO-d6) δ 164.39, 163.49, 161.82, 160.23, 157.12, 157.03, 150.23, 138.05, 134.85, 127.44, 126.84, 123.90, 122.38, 122.27, 115.55, 115.25, 61.40, 14.07. MS(ESI): *m*/*z* 367.35 [M + H]^+^.

*6-(5-((4-fluorophenyl)carbamoyl)pyrimidin-2-yl)nicotinic acid* (**13u**): Grey solid, melting point: 290–291 °C, Yield: 65%, ^1^H NMR (300 MHz, DMSO-*d*_6_) δ 13.62 (s, 1H), 10.81 (s, 1H), 9.46 (s, 2H), 9.25 (s, 1H), 8.58 (d, J = 7.8 Hz, 1H), 8.47 (d, *J* = 7.5 Hz, 1H), 7.82 (dd, *J* = 8.7, 5.1 Hz, 2H), 7.23 (t, *J* = 8.8 Hz, 2H). ^13^C NMR (75 MHz, DMSO-d6) δ 165.91, 163.47, 161.80, 160.17, 157.16, 156.98, 150.49, 138.21, 134.86, 127.66, 127.32, 123.83, 122.33, 122.23, 115.51, 115.22. MS(ESI): *m*/*z* 339.26 [M + H]^+^.

### 3.3. MTT Assay

HSC-T6 cells were obtained from National Science and Technology Infrastructure. To determine the half-maximal inhibitory concentration (IC_50_) of the compounds we synthesized, HSC-T6 cells were seeded into 96-well with a density of 2000 cells/well in DMEM culture medium with 10% fetal bovine serum in a humidified atmosphere containing 5% CO_2_. After attachment (about 24 h), the cells were starved in a serum-free medium for about 12 h to induce HSC-T6 transformation into a static period as well as synchronization, and then compounds to be tested were added to corresponding wells with different concentrations in culture medium with 2% fetal bovine serum. After 48 h of incubation, 20 μL of 3-[4,5-dimethyl-2-thiazolyl)-2,5-diphenyl-2H-tetrazolium bromide (MTT) solution (5 mg/mL) was added to each well and the plates were incubated for an additional 4 h. Subsequently, the medium was aspirated carefully, and 150 μL of DMSO was added to dissolve the crystal. The optical density was measured at 490 nm using a RT-2100C Microplate Reader (Rayto, Shenzhen, China). The results were used to calculate IC_50_ values. Each experiment was repeated three times.

### 3.4. Hydroxyproline Assay

Cells were plated on 96-wells with a density of 7000 cells/well. After attachment (24 h), each compound, at different concentrations, was added to cells. After 48 h of incubation, cell culture supernatants were taken out for the next step. The hydroxyproline contents in cell culture supernatants were measured with a conventional hydroxyproline assay kit (A030-2-1, Nanjing Jiancheng Bioengineering Institute, Nanjing, China). Subsequently, the hydroxyproline analysis was performed using Chloramine-T spectrophotometric absorbance.

### 3.5. Picro-Sirius Red (PSR) Staining

The HSC-T6 cells cultured in 6-well plates were carefully washed twice with phosphate buffered saline (PBS), xylene, ethanol and incubated in the PSR staining solution (SIRIUS RED.0.1% in saturated picric acid 26357-02, Electron Microscopy Organizer Sciences, Lot no.:190628-01) at room temperature for 1 h. The staining solution was then removed and the cells were washed three times with ethanol. Then, the HSC-T6 cells were incubated in the hematoxylin staining solution at room temperature for 10 min. The hematoxylin staining solution was removed and the cells were washed three times with ethanol and kept in xylene for 5 min. The stained cells were dried and a picture was taken with a microscope.

### 3.6. ELISA Detection for COL1A1

HSC-T6 cells were plated on 96-wells with a density of 7000 cells/well. After attachment (24 h), each compound, at different concentrations, was added to the remaining cell lines mentioned above. After 48 h of incubation, cell culture supernates were centrifuged for 20 min at 1000× *g*. A quantity of 50 μL of standard or cell culture supernates was added to the appropriate wells and 100 μL of Enzymeconjugate was added to standard wells and sample wells, except for the blank well. After 1 h of incubation at 37 °C, the Microtiter Plate was washed 4 times. Then, Substrate A 50 μL and Substrate B 50 μL were added to each well (Rat ColIELISA KIT, RUIXIN Biology, Guangzhou, China, lot:07/2020). After 15 min of incubation at 37 °C, 50 μL Stop Solution was added to each well and the optical density was measured at 490 nm using RT-2100C Microplate Reader.

## 4. Conclusions

In conclusion, we have successfully prepared a representative library of 2-(pyridin-2-yl)pyrimidine derivatives by starting from the readily available nicotinic acid and 1H-pyrazole-4-carboxylic acid. In addition, those compounds were evaluated for their anti-fibrotic activities in vitro. Among them, fourteen compounds exhibited inhibitory activities stronger than Pirfenidone, 24PDC and Bipy55′DC, with **12m** and **12q** displaying activities with IC_50_ values of 45.69 μM and 45.81 μM against HSC-T6, respectively. Furthermore, the results of hydroxyproline assay displayed that **12m** and **12q** might be inhibitors of collagen prolyl-4-hydroxylase. In addition, hydroxyproline assay and Picro-Sirius red (PSR) staining of compounds **12m** and **12q** exhibited potent anti-fibrosis abilities in alleviating the total collagen accumulation in HSC-T6 cells in a dose-dependent manner. Further, ELISA results for compounds **12m** and **12q** showed better activity in alleviating COL1A1 in HSC-T6 cells. The in-depth mechanisms of action of the compounds **12m** and **12q** remain to be further investigated.

## Figures and Tables

**Table 1:** The IC_50_ values of the compounds **12a**–**12t** and **13a**–**13t** by MTT assay.

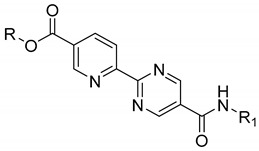							
Compd	R	R_1_	IC_50_^a^/(μmol·L^−^^1^)	Compd	R	R_1_	IC_50_^a^/(μmol·L^−^^1^)
**12a**	Et	p-F-C_6_H_4_CH_2_-	>1000	**13a**	H	p-F-C_6_H_4_CH_2_-	>1000
**12b**	Et	p-Cl-C_6_H_4_CH_2_-	>1000	**13b**	H	p-Cl-C_6_H_4_CH_2_-	>1000
**12c**	Et	p-Br-C_6_H_4_CH_2_-	>1000	**13c**	H	p-Br-C_6_H_4_CH_2_-	174.553 ± 1.872
**12d**	Et	p-CH_3_-C_6_H_4_CH_2_-	71.465 ± 1.854	**13d**	H	p-CH_3_-C_6_H_4_CH_2_-	>1000
**12e**	Et	p-CH_3_O-C_6_H_4_CH_2_-	>1000	**13e**	H	p-CH_3_O-C_6_H_4_CH_2_-	>1000
**12f**	Et	m-Br-C_6_H_4_CH_2_-	>1000	**13f**	H	m-Br-C_6_H_4_CH_2_-	>1000
**12g**	Et	m-CH_3_-C_6_H_4_CH_2_-	>1000	**13g**	H	m-CH_3_-C_6_H_4_CH_2_-	266.06 ± 2.22
**12h**	Et	o-F-C_6_H_4_CH_2_-	>1000	**13h**	H	o-F-C_6_H_4_CH_2_-	>1000
**12i**	Et	m,p-2F-C_6_H_3_CH_2_-	>1000	**13i**	H	m,p-2F-C_6_H_3_CH_2_-	>1000
**12j**	Et	m-F-C_6_H_4_CH_2_-	>1000	**13j**	H	m-F-C_6_H_4_CH_2_-	>1000
**12k**	Et	p-Cl-C_6_H_4_-	47.415 ± 1.351	**13k**	H	p-Cl-C_6_H_4_-	>1000
**12l**	Et	p-Br-C_6_H_4_-	51.532 ± 1.062	**13l**	H	p-Br-C_6_H_4_-	156.539 ± 2.981
**12m**	Et	p-CH_3_-C_6_H_4_-	45.693 ± 1.660	**13m**	H	p-CH_3_-C_6_H_4_-	>1000
**12n**	Et	m-Br-C_6_H_4_-	200.946 ± 2.303	**13n**	H	m-Br-C_6_H_4_-	>1000
**12o**	Et	p-CH_3_O-C_6_H_4_-	67.379 ± 1.240	**13o**	H	p-CH_3_O-C_6_H_4_-	>1000
**12p**	Et	m-CH_3_-C_6_H_4_-	259.261 ± 2.414	**13p**	H	m-CH_3_-C_6_H_4_-	220.356 ± 2.691
**12q**	Et	m,p-2F-C_6_H_3_-	45.819 ± 1.747	**13q**	H	m,p-2F-C_6_H_3_-	>1000
**12r**	Et	m-F-C_6_H_4_-	63.728 ± 1.804	**13r**	H	m-F-C_6_H_4_-	>1000
**12s**	Et	o-F-C_6_H_4_-	92.777 ± 1.967	**13s**	H	o-F-C_6_H_4_-	>1000
**12t**	Et	Et	>1000	**13t**	H	Et	>1000
**12u**	Et	p-F-C_6_H_4_-	369.324 ± 2.351	**13u**	H	p-F-C_6_H_4_-	>1000
**24PDC** ^b^			>1000	**Bipy55′DC** ^b^			>1000
**PFD ^b^**			3025 ± 0.481				

^a^ Inhibitory activity was assayed by exposure to substances for 48 h and expressed as concentration required to inhibit HSC-T6 by 50% (IC_50_). ^b^ Positive control.

## Supplementary Materials

The following are available online. Figures are ^1^H NMR and ^13^C NMR of the synthesized compounds.
