# Supplementary material for: Synthesis of Novel 2-(Pyridin-2-yl) Pyrimidine Derivatives and Study of Their Anti-Fibrosis Activity
Source: Molecules. 2020 Nov 10;25(22):5226. doi: 10.3390/molecules25225226 (PMC7697764; doi:10.3390/molecules25225226)
Supplement: Supplementary file 1 [file molecules-25-05226-s001.pdf]

molecules

MDPI

Supporting Information

Synthesis of Novel 2-(Pyridin-2-yl) Pyrimidine Derivatives and Study of Their Anti-Fibrosis Activity

Yi-Fei Gu [1], Yue Zhang [1], Feng-li Yue [2], Shao-tong Li [2], Zhuo-qi Zhang [1], Jing Li [2,*] and Xu Bai [1,*]

[1] The Center for Combinatorial Chemistry and Drug Discovery of Jilin University, The School of Pharmaceutical Sciences, Jilin University, 1266 Fujin Road, Changchun, Jilin 130021, China; guyifei2007@163.com (Y.-F.G.); zy2932886799@outlook.com (Y.Z.); zqzhang@jlu.edu.cn (Z.-q.Z.)

[2] Department of Pharmacology, College of Basic Medical Sciences, Jilin University, Changchun 130021, China; yuefl18@mails.jlu.edu.cn (F.-l.Y.); gaby9905@163.com (S.-t.L.)

* Correspondence: lijing@jlu.edu.cn (J.L.); xbai@jlu.edu.cn (X.B.); Tel.: +86-431-8561-9260 (X.B.)

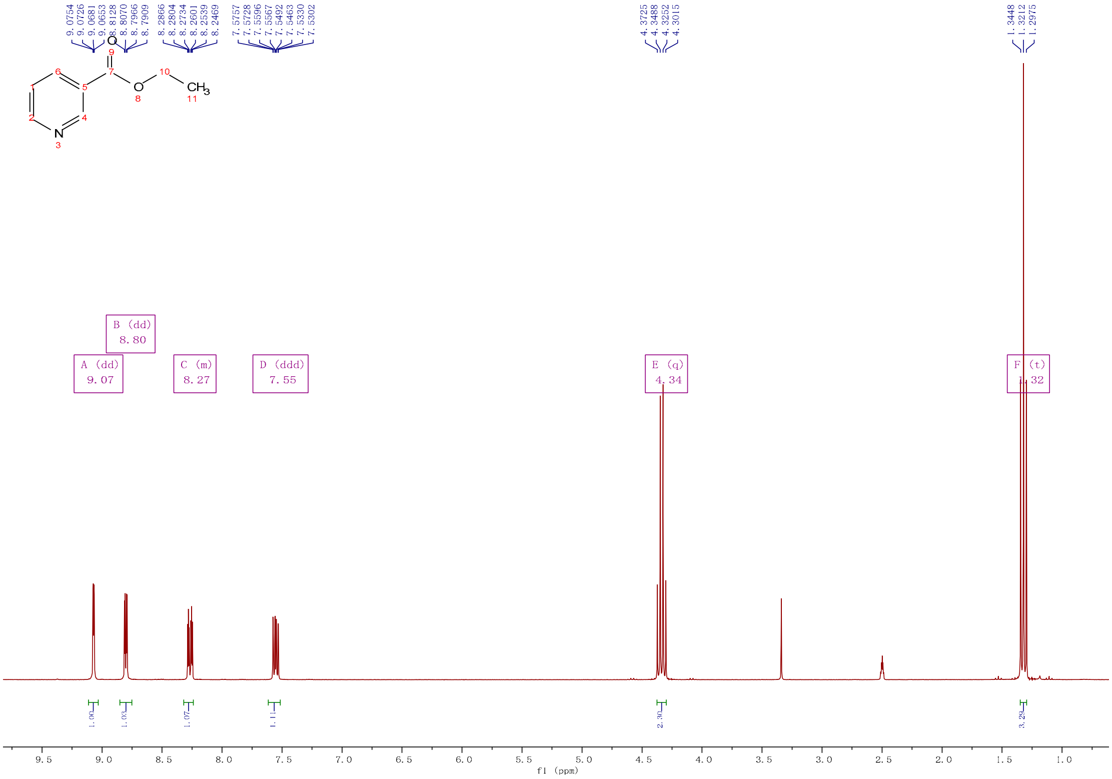

Figure S1: [1] H-NMR of spectrum compound 2

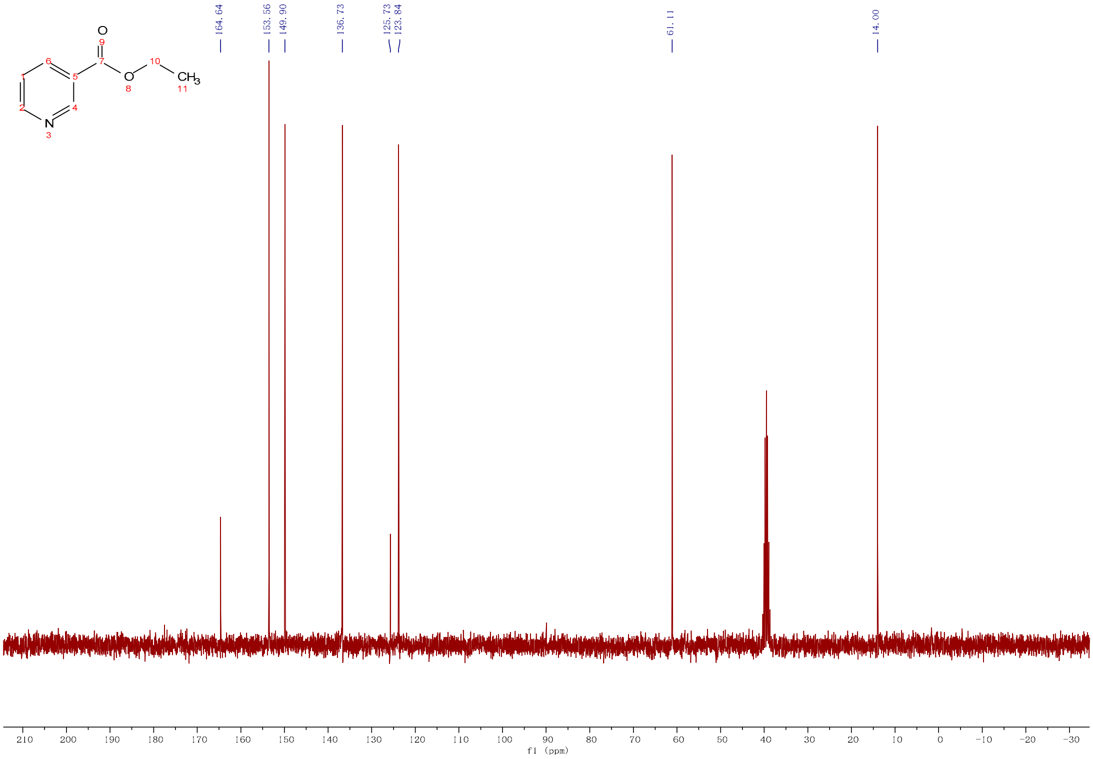

Figure S2: ^{13}C -NMR of spectrum compound 2

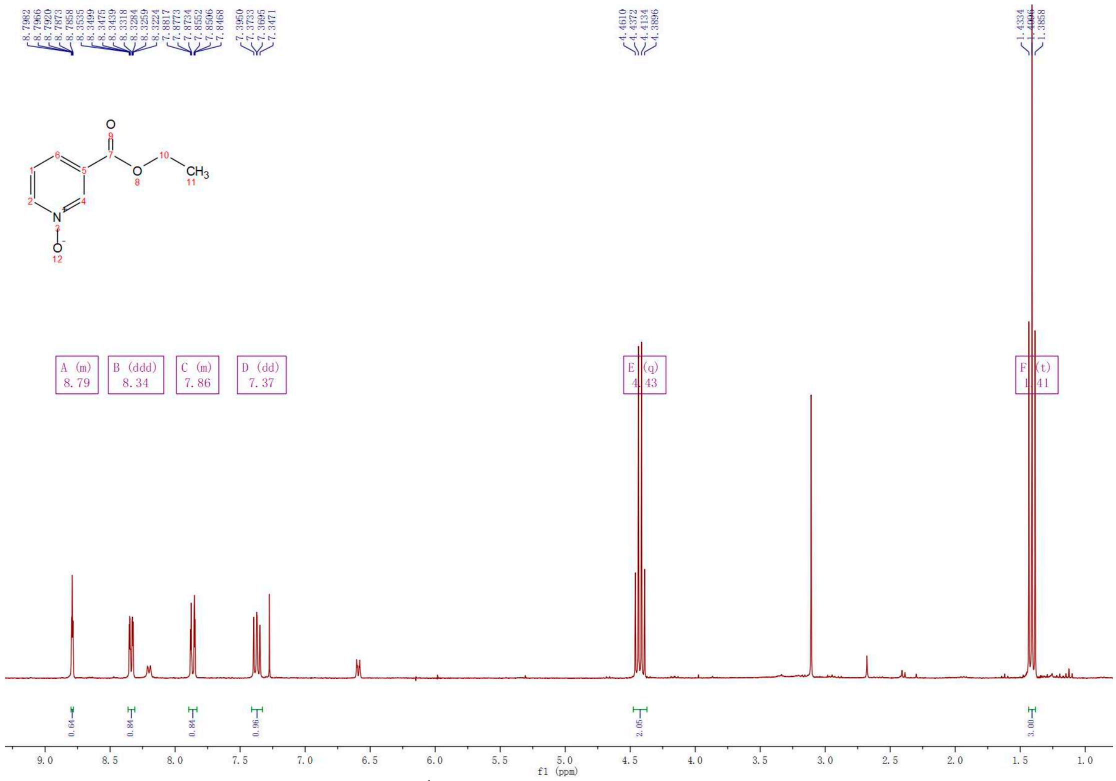

Figure S3: [1] H-NMR of spectrum compound 3

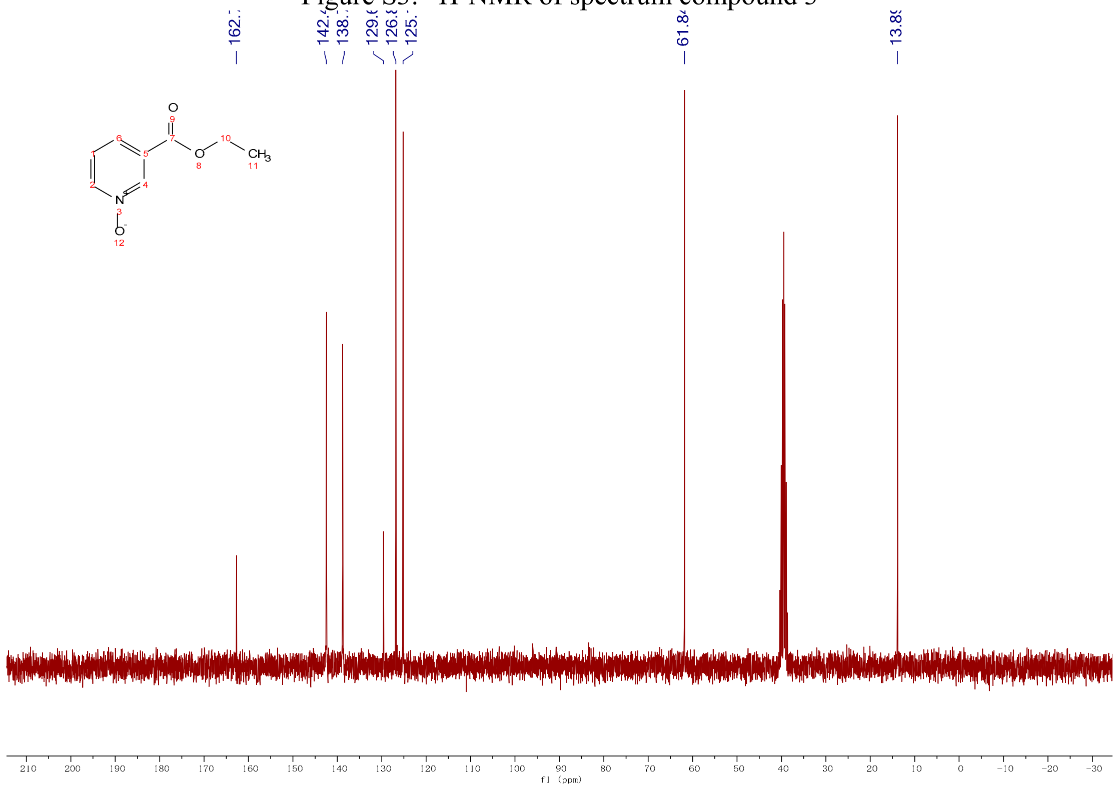

Figure S4: ^{13}C -NMR of spectrum compound 3

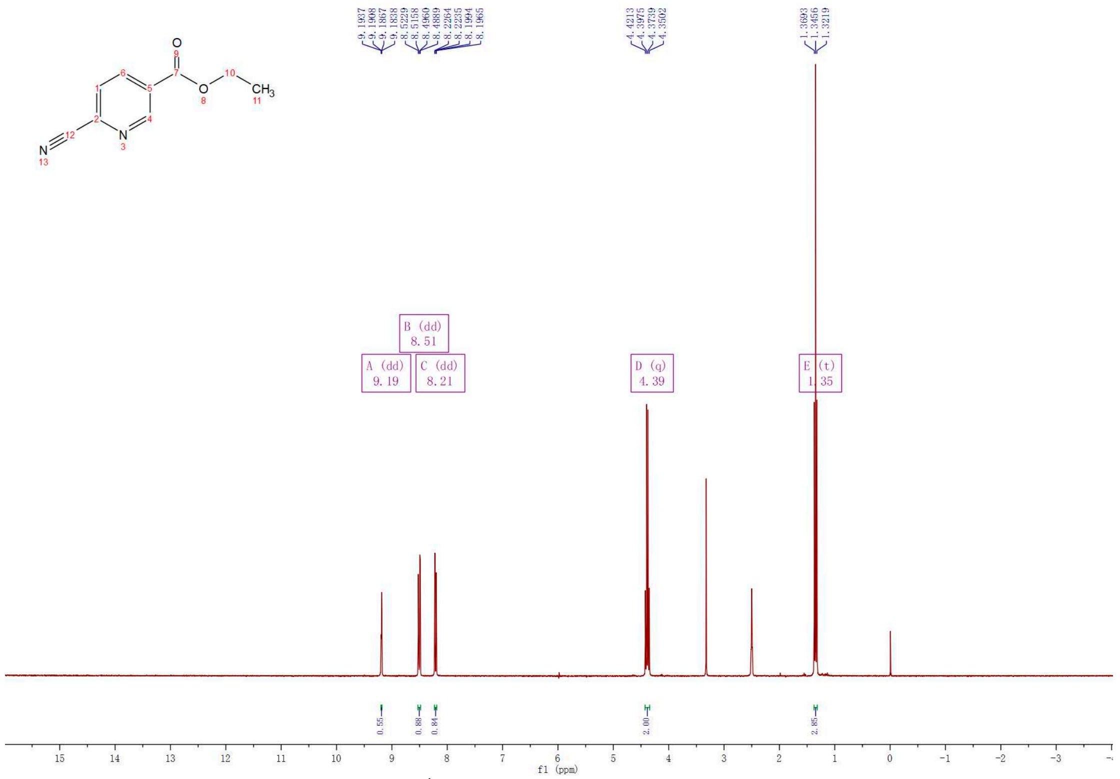

Figure S5: [1] H-NMR of spectrum compound 4

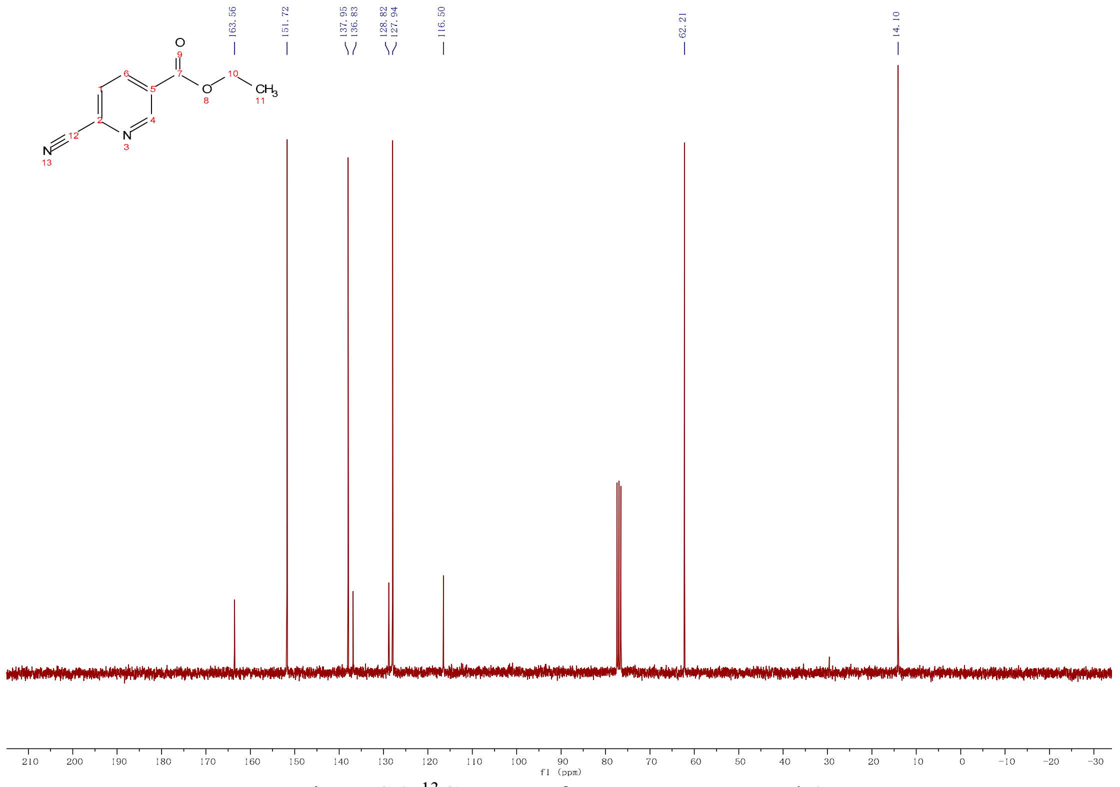

Figure S6: ^{13}C -NMR of spectrum compound 4

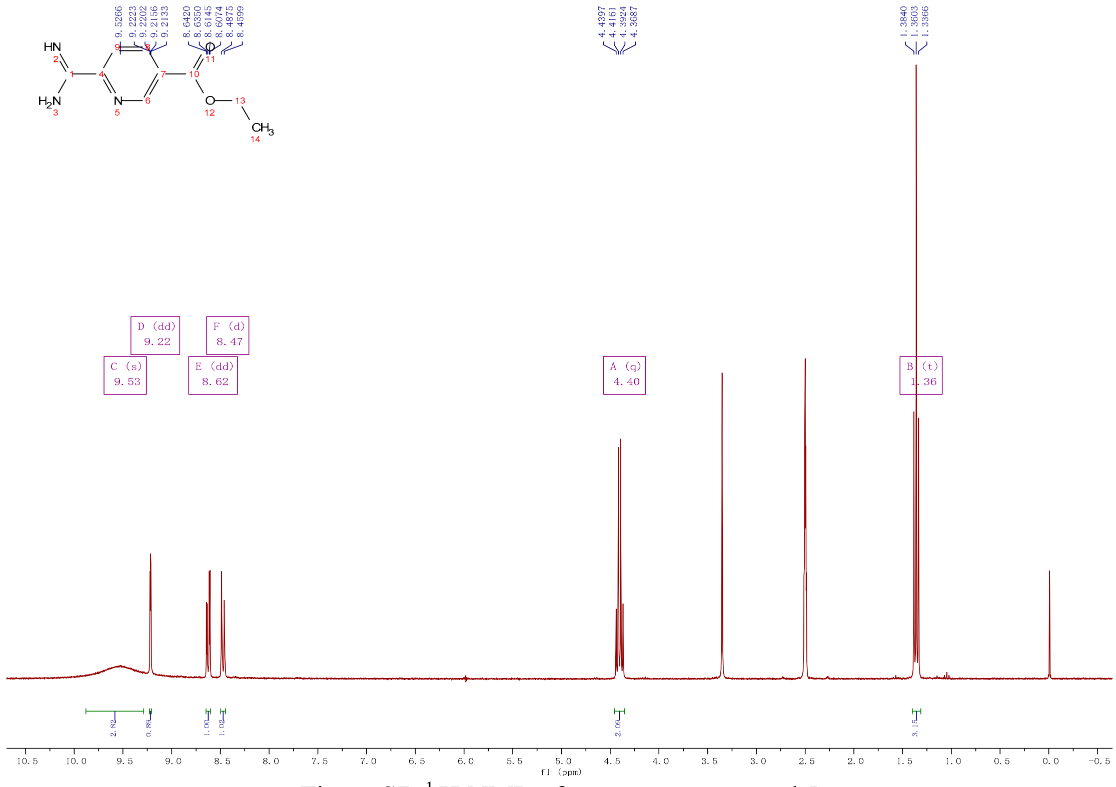

Figure S7: ^{1}H-NMR of spectrum compound 5

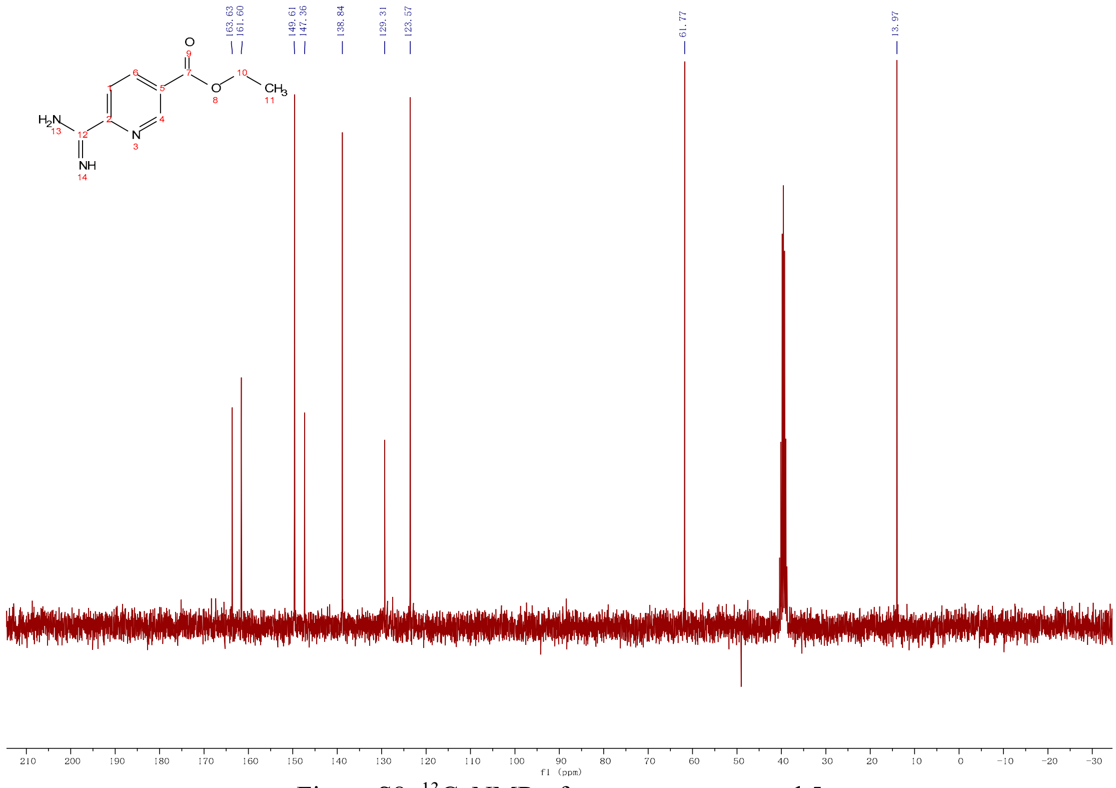

Figure S8: ^{13}C -NMR of spectrum compound 5

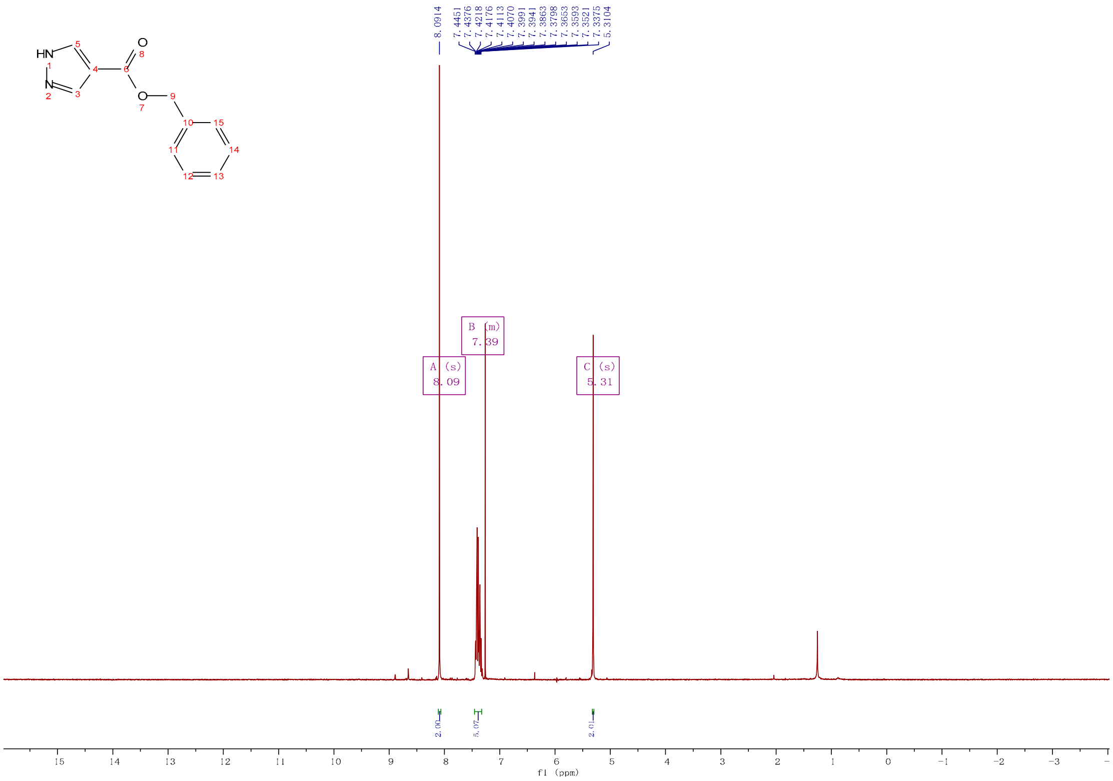

Figure S9: [1] H-NMR of spectrum compound 7

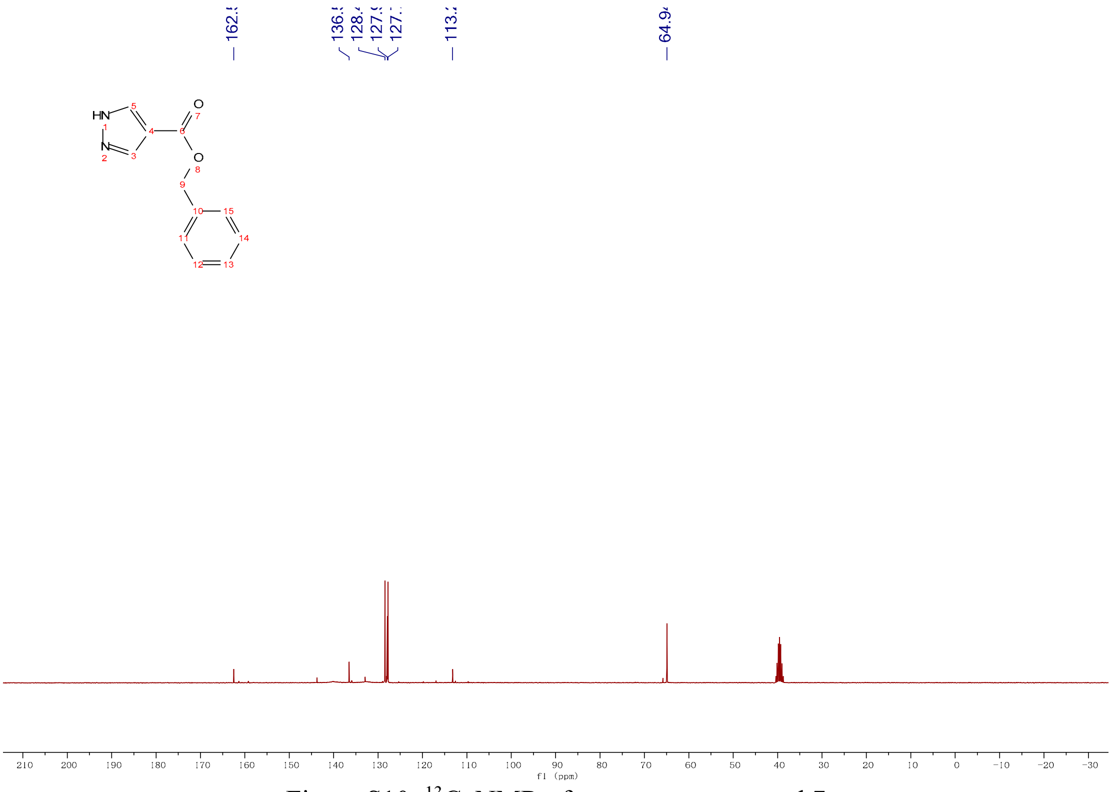

Figure S10: ^{13}C -NMR of spectrum compound 7

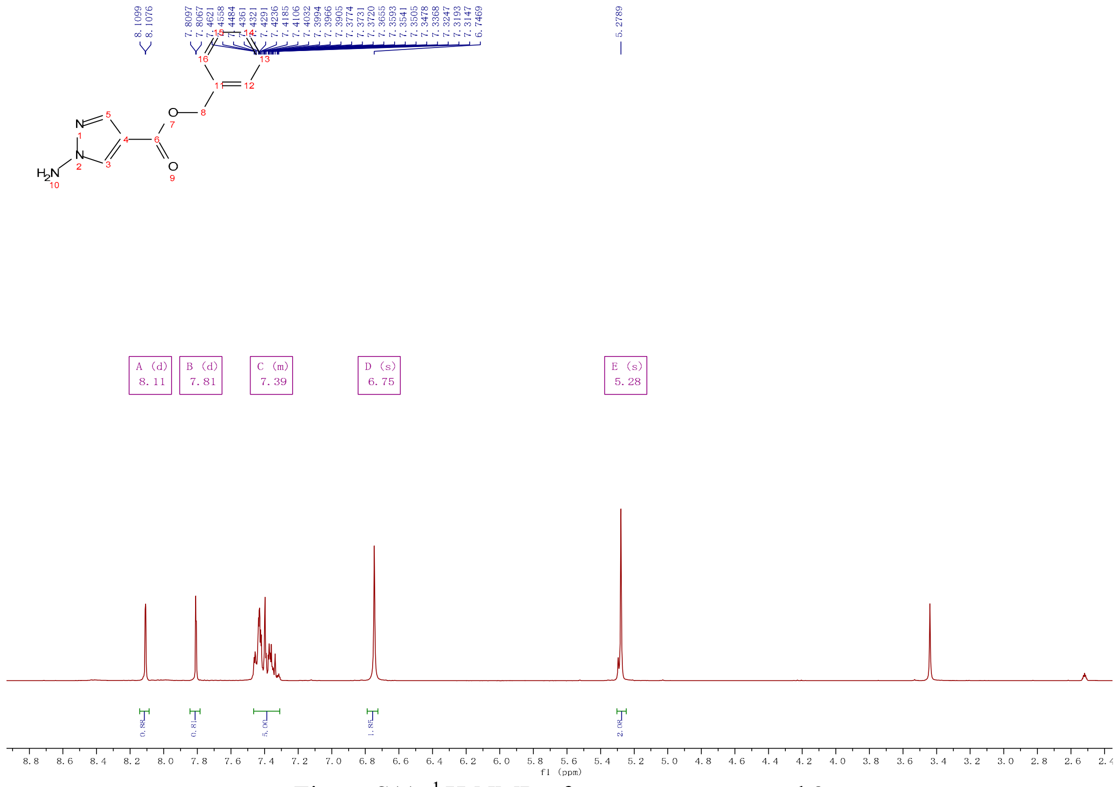

Figure S11: [1] H-NMR of spectrum compound 8

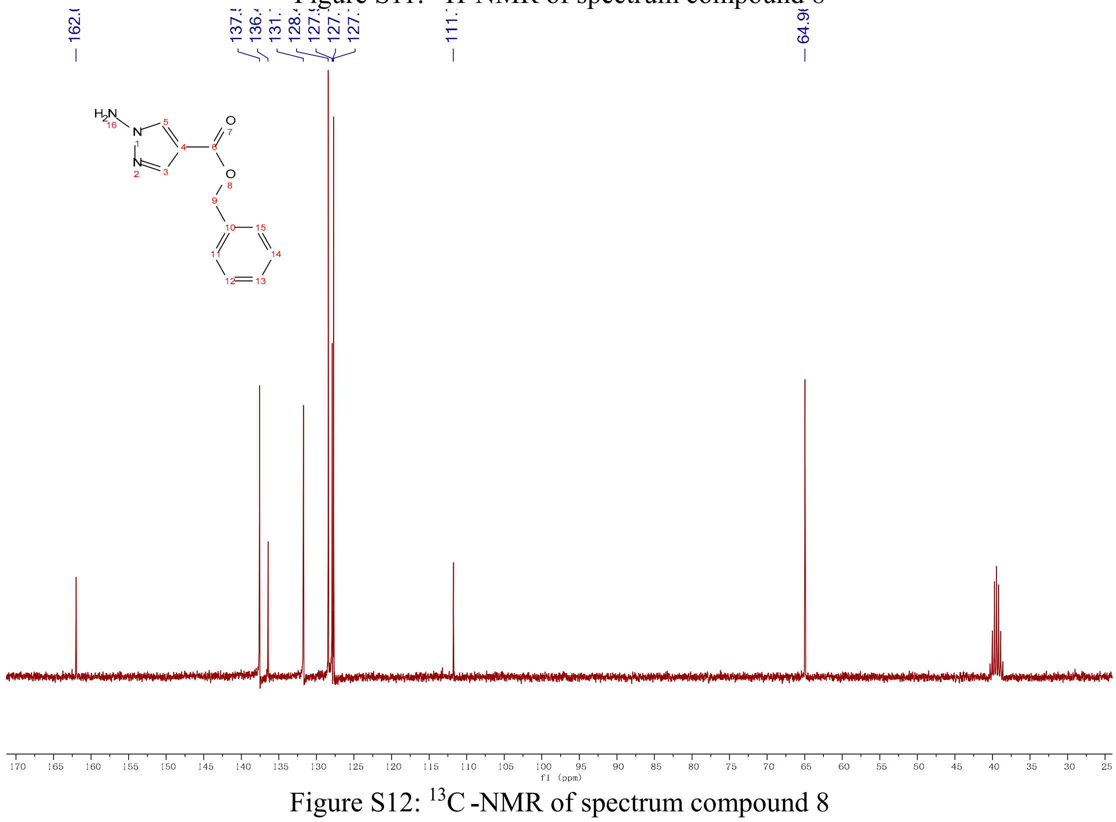

Figure S12: ^{13}C -NMR of spectrum compound 8

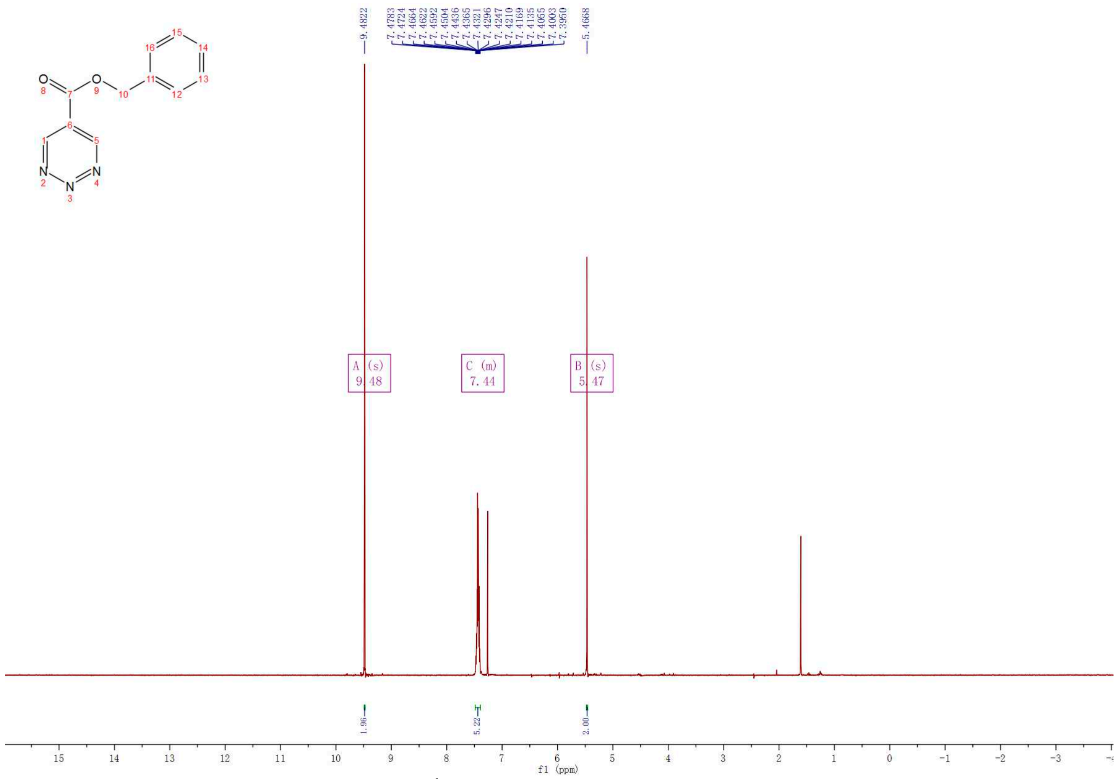

Figure S13: ^{1}H-NMR of spectrum compound 9

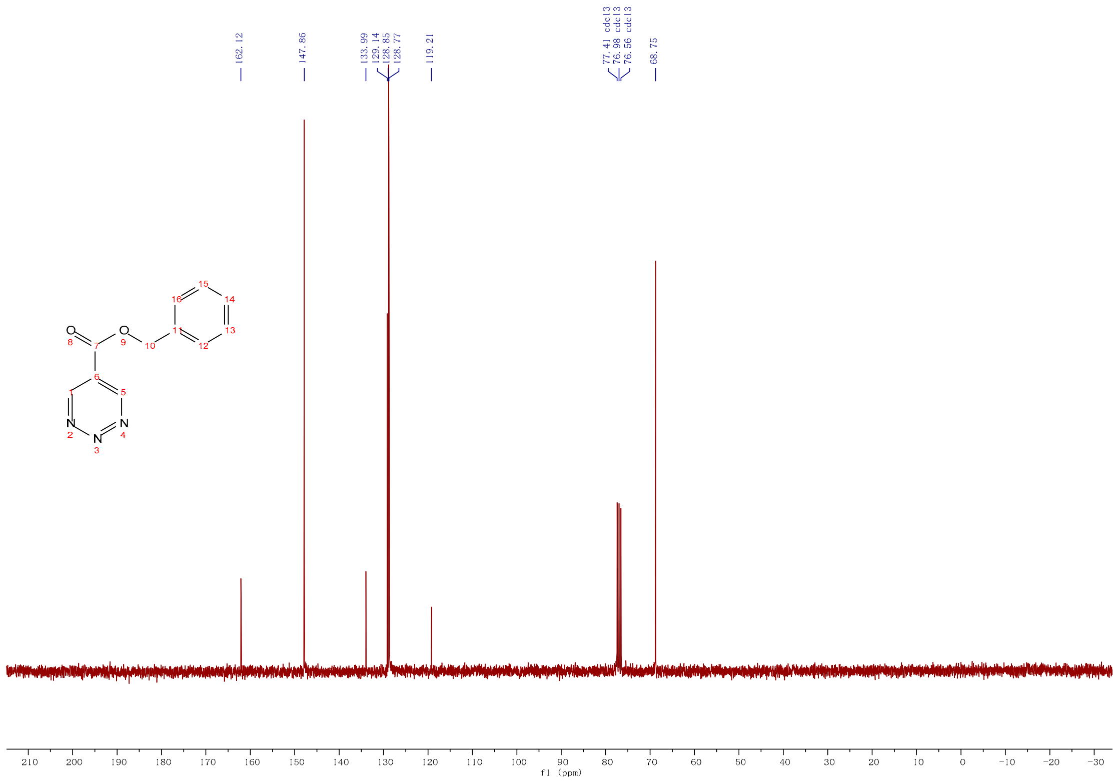

Figure S14: ^{13}C -NMR of spectrum compound 9

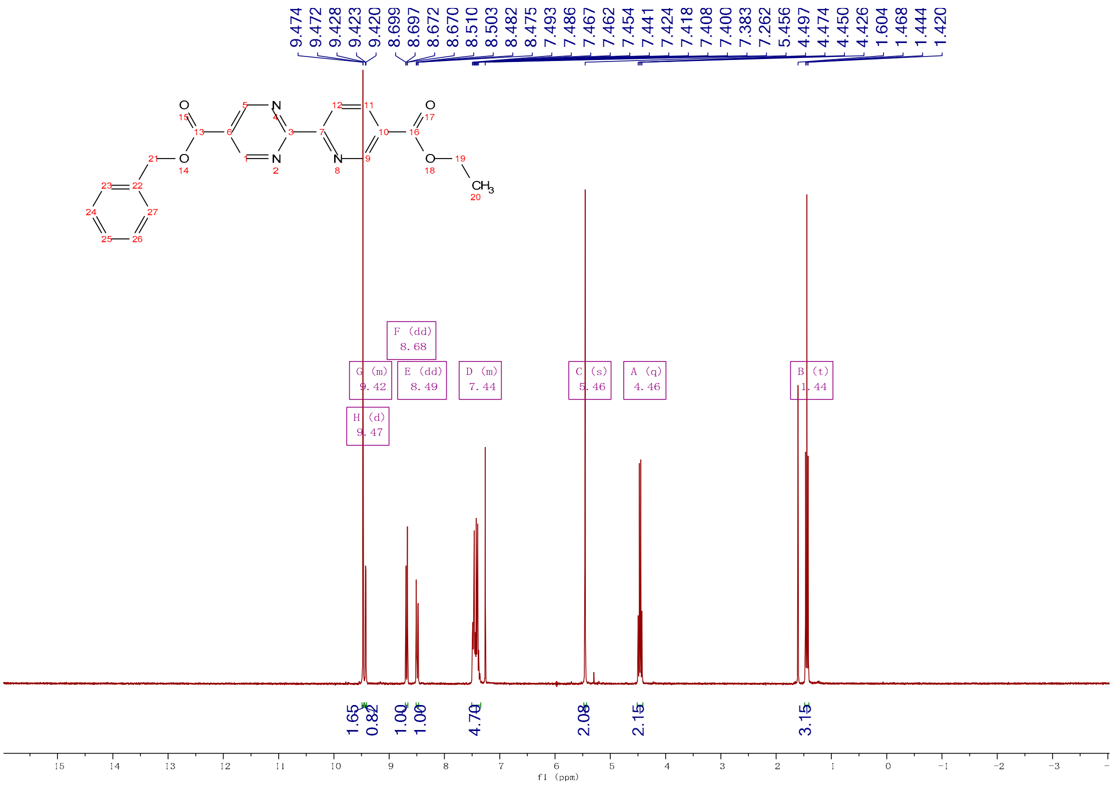

Figure S15: ^{1}H-NMR of spectrum compound 10

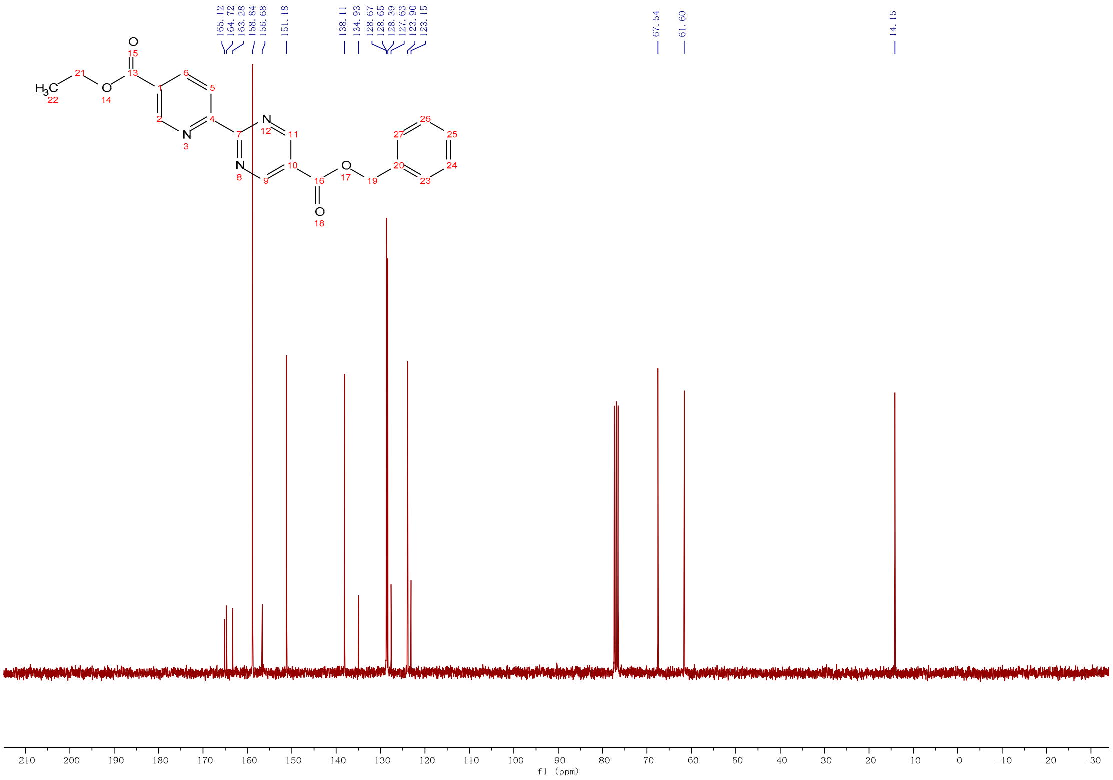

Figure S16: ^{13}C -NMR of spectrum compound 10

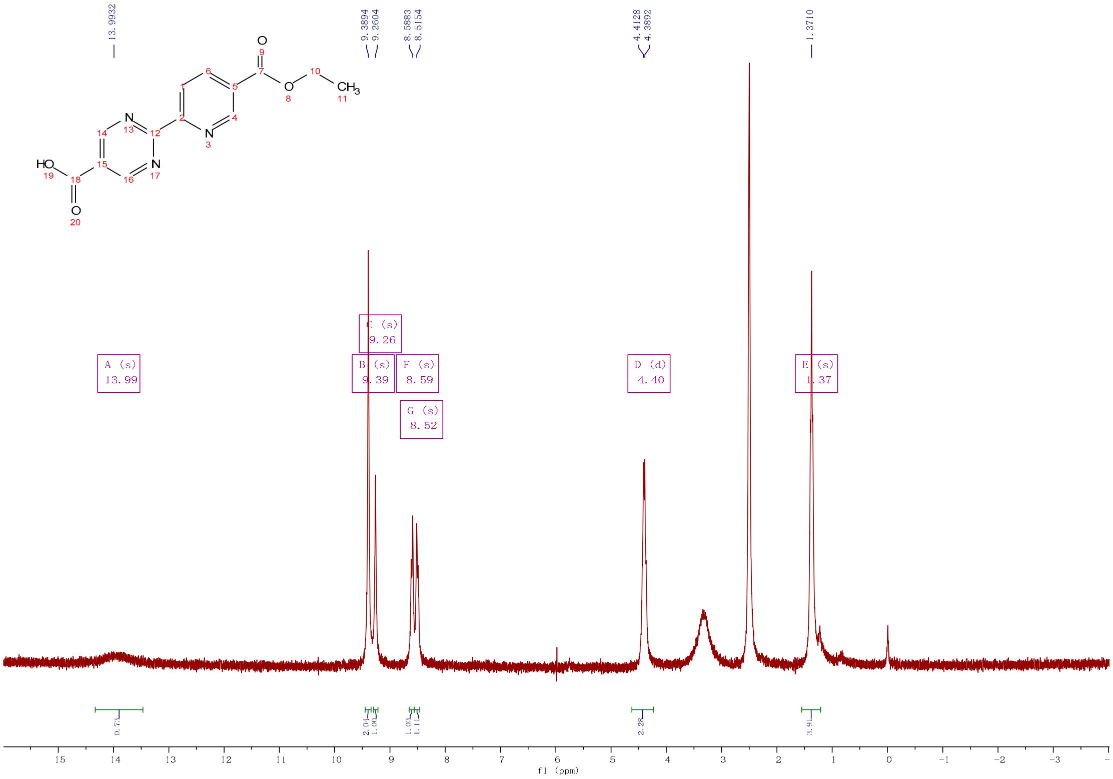

Figure S17: ^{1}H-NMR of spectrum compound 11

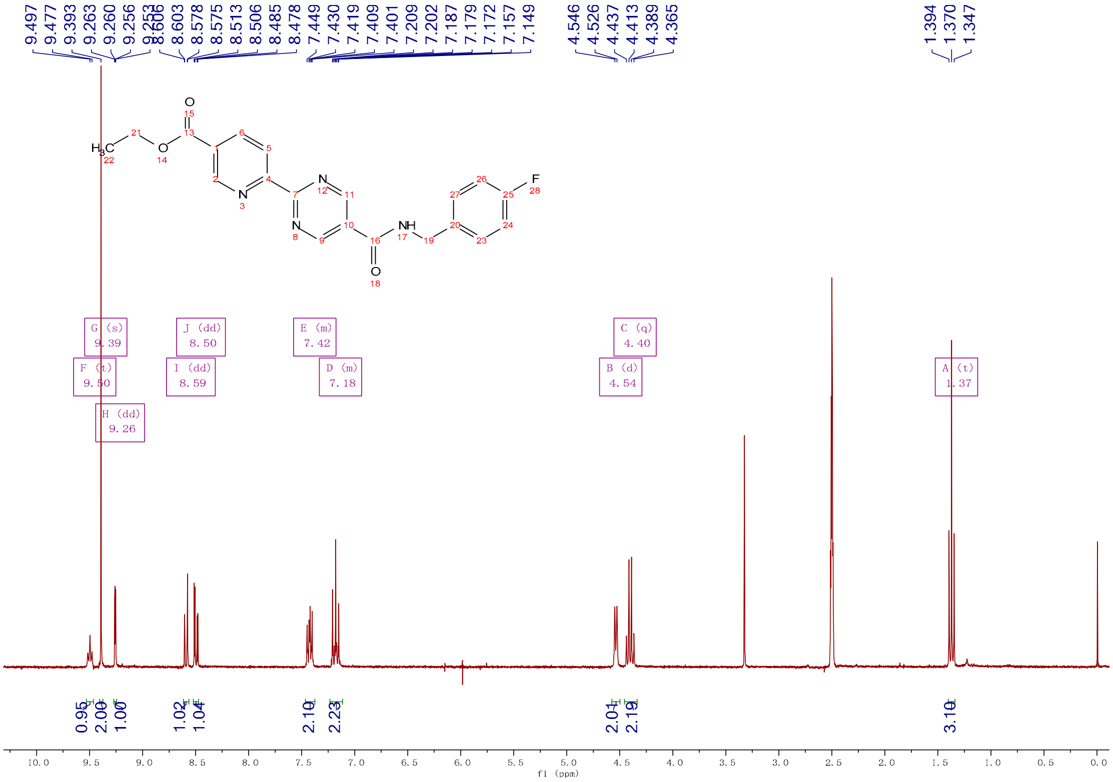

Figure S18: ^{1}H-NMR of spectrum compound 12a

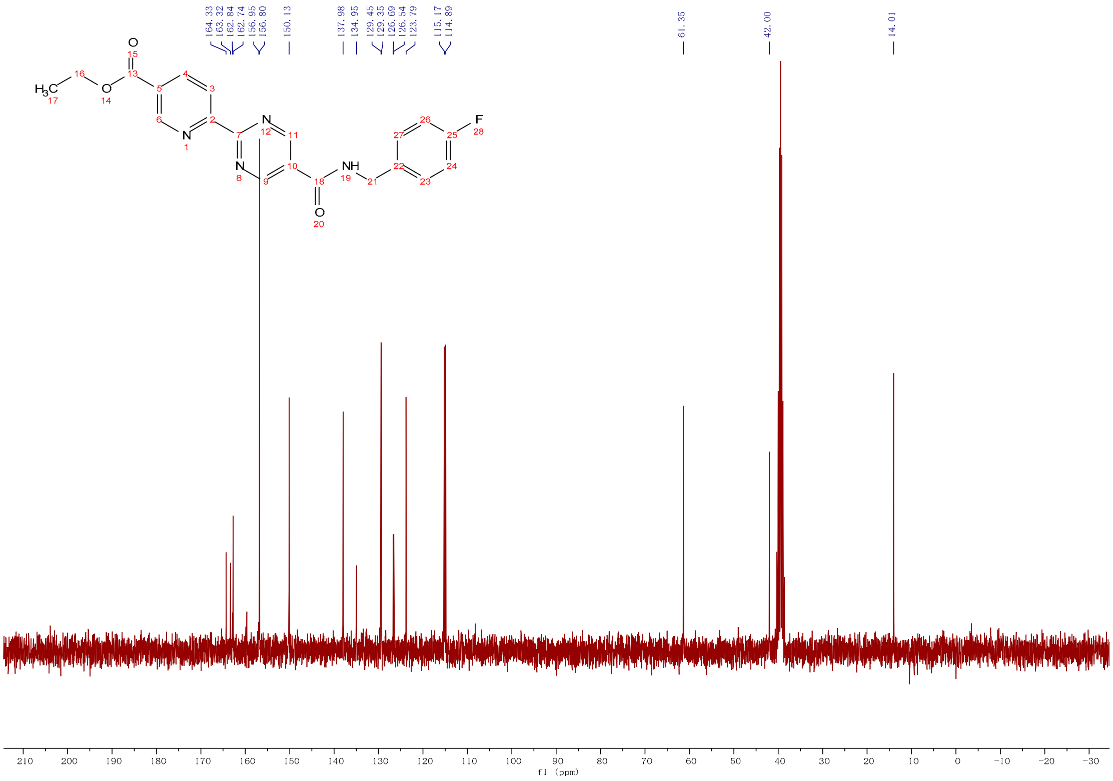

Figure S19: ^{13}C -NMR of spectrum compound 12a

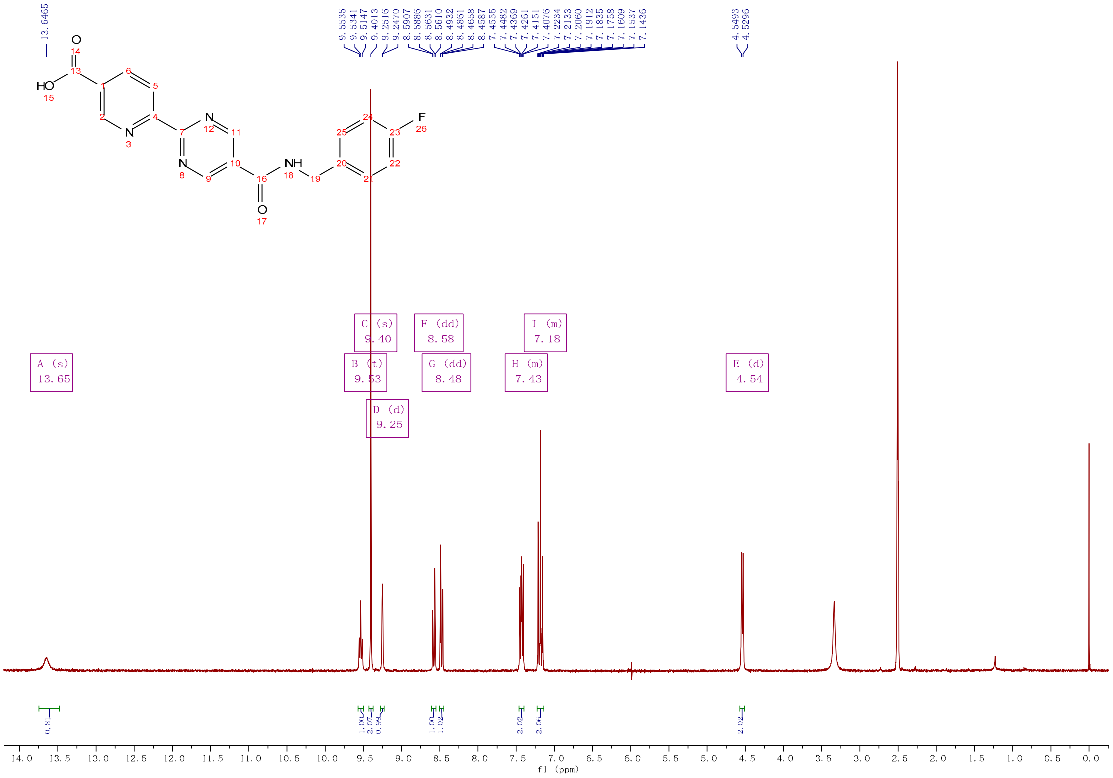

Figure S20: ^{1}H-NMR of spectrum compound 13a

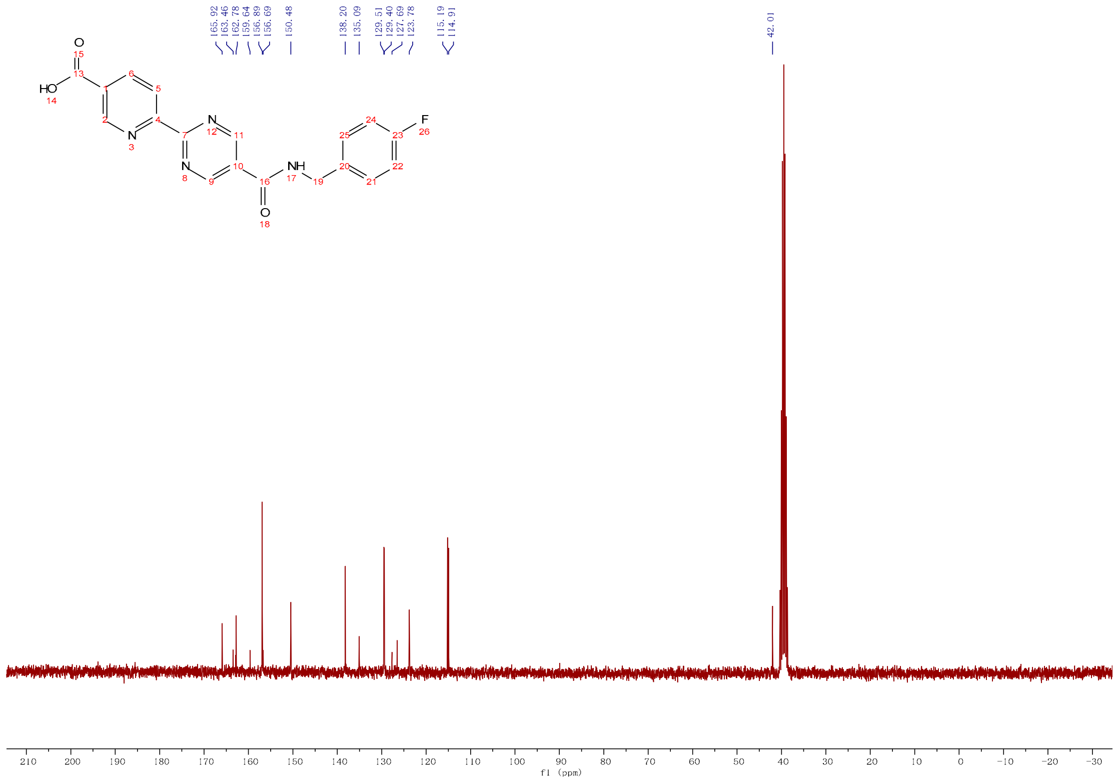

Figure S21: ^{13}C -NMR of spectrum compound 13a

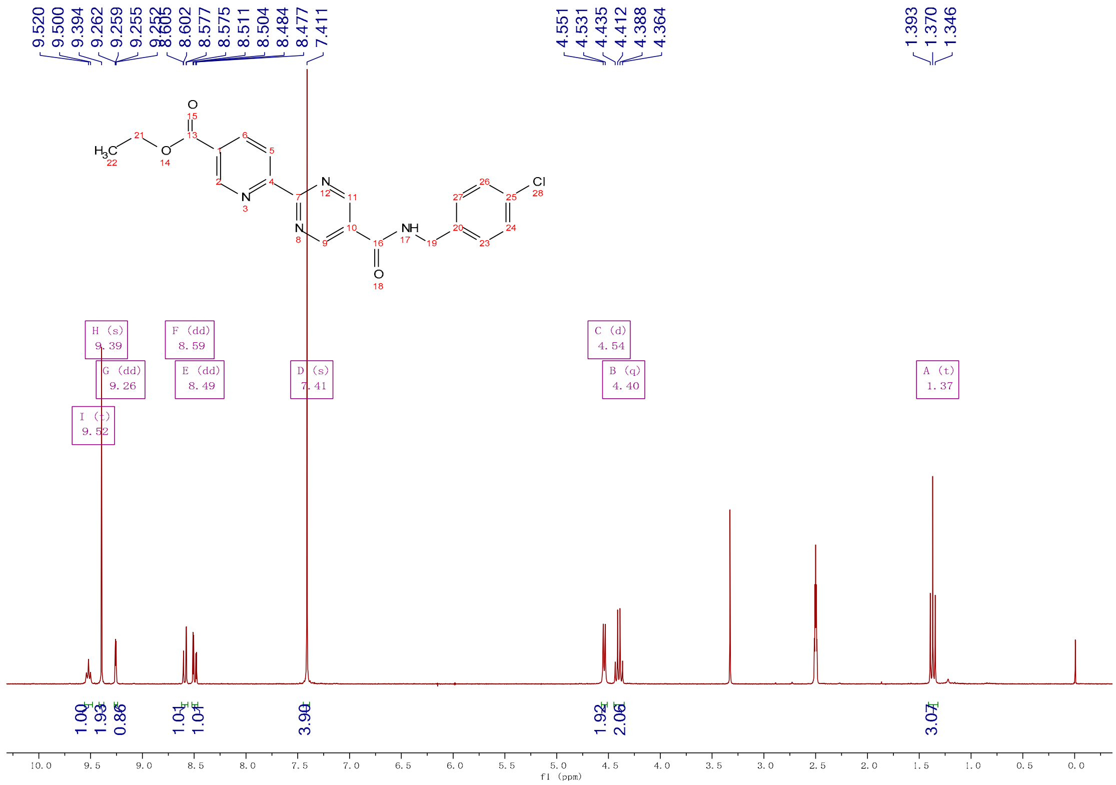

Figure S22: ^{1}H-NMR of spectrum compound 12b

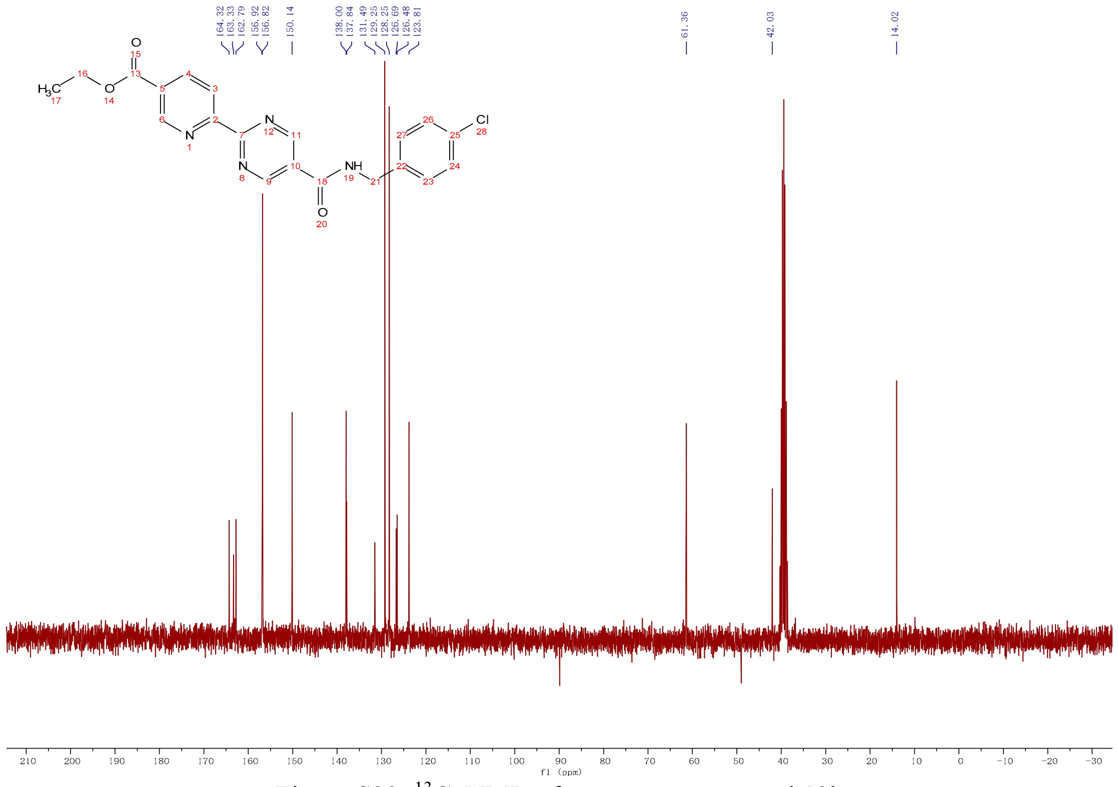

Figure S23: ^{13}C -NMR of spectrum compound 12b

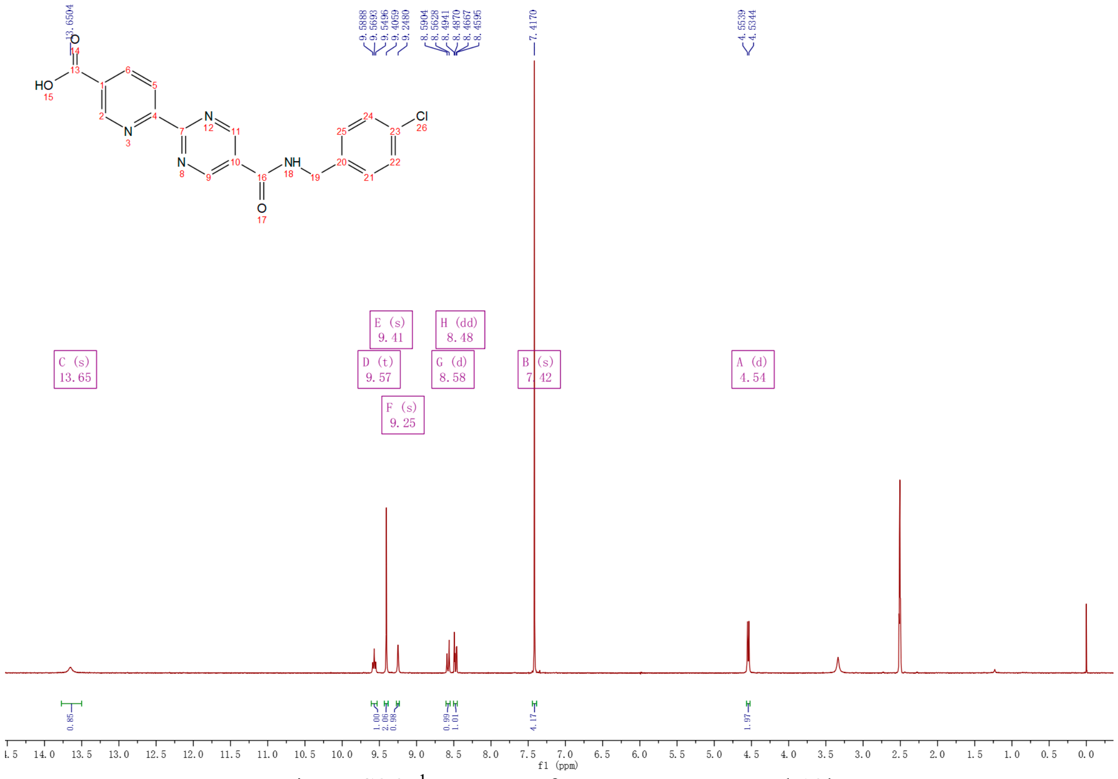

Figure S24: ^{1}H-NMR of spectrum compound 13b

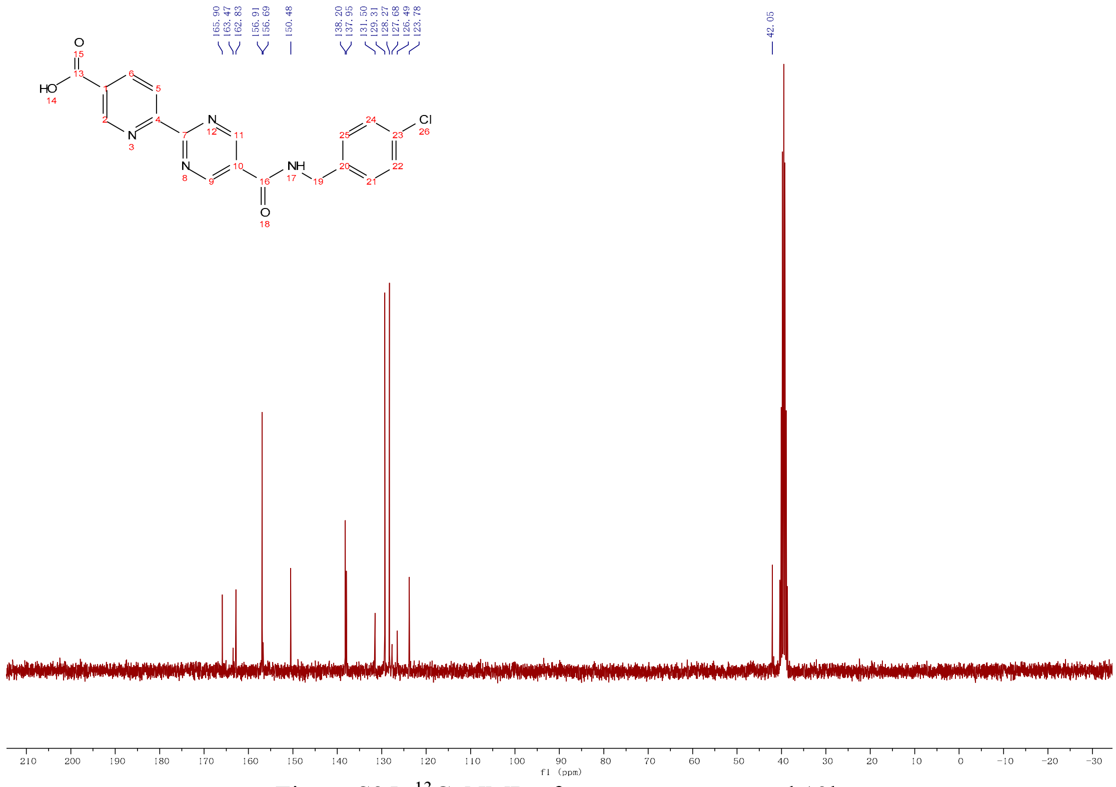

Figure S25: ^{13}C -NMR of spectrum compound 13b

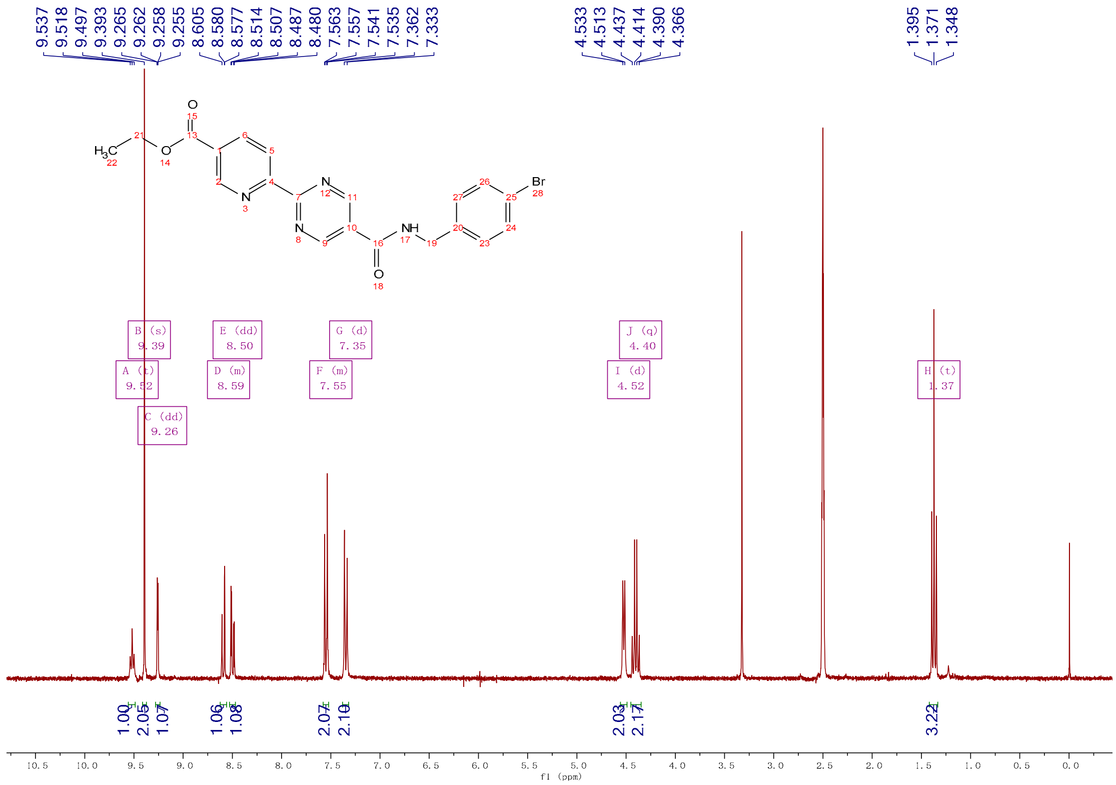

Figure S26: ^{1} H-NMR of spectrum compound 12c

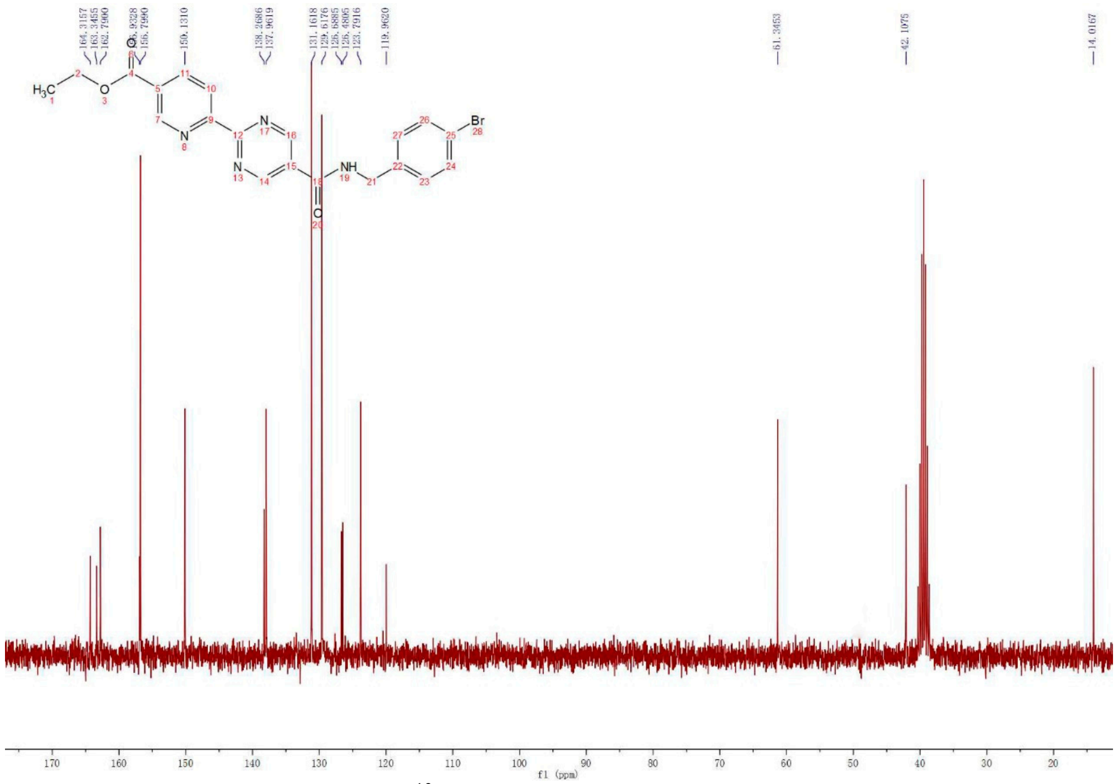

Figure S27: ^{13}C -NMR of spectrum compound 12c

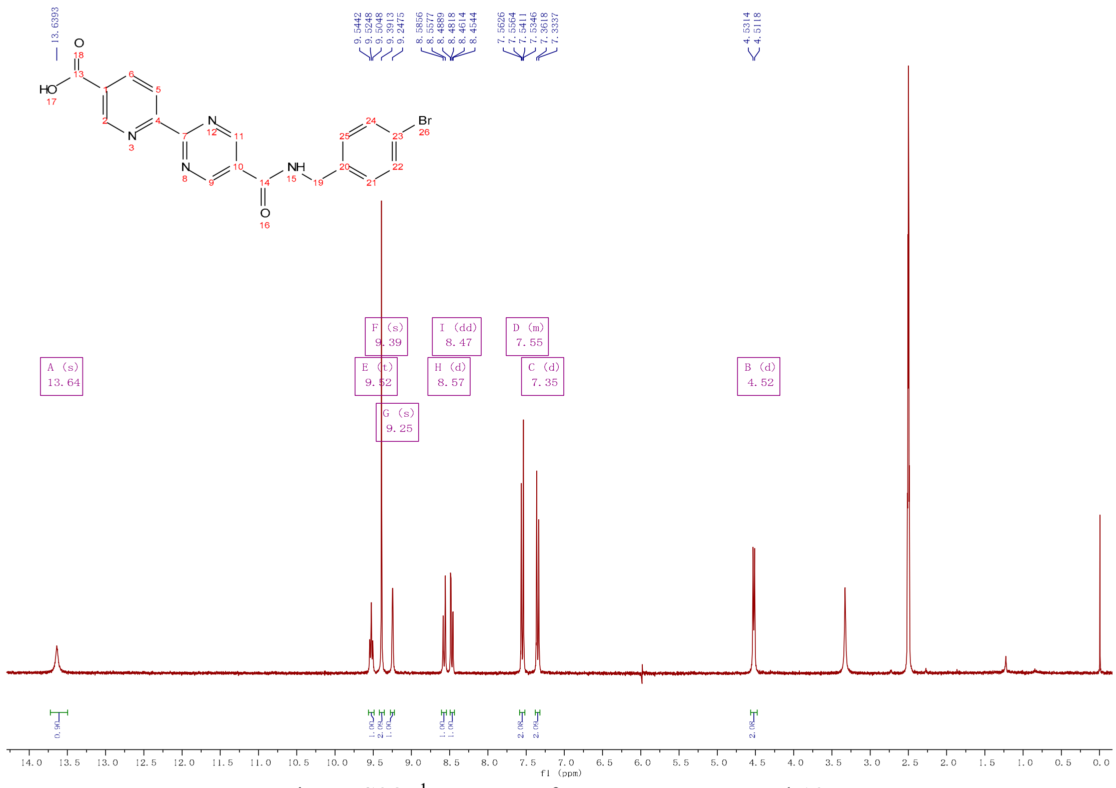

Figure S28: ^{1} H-NMR of spectrum compound 13c

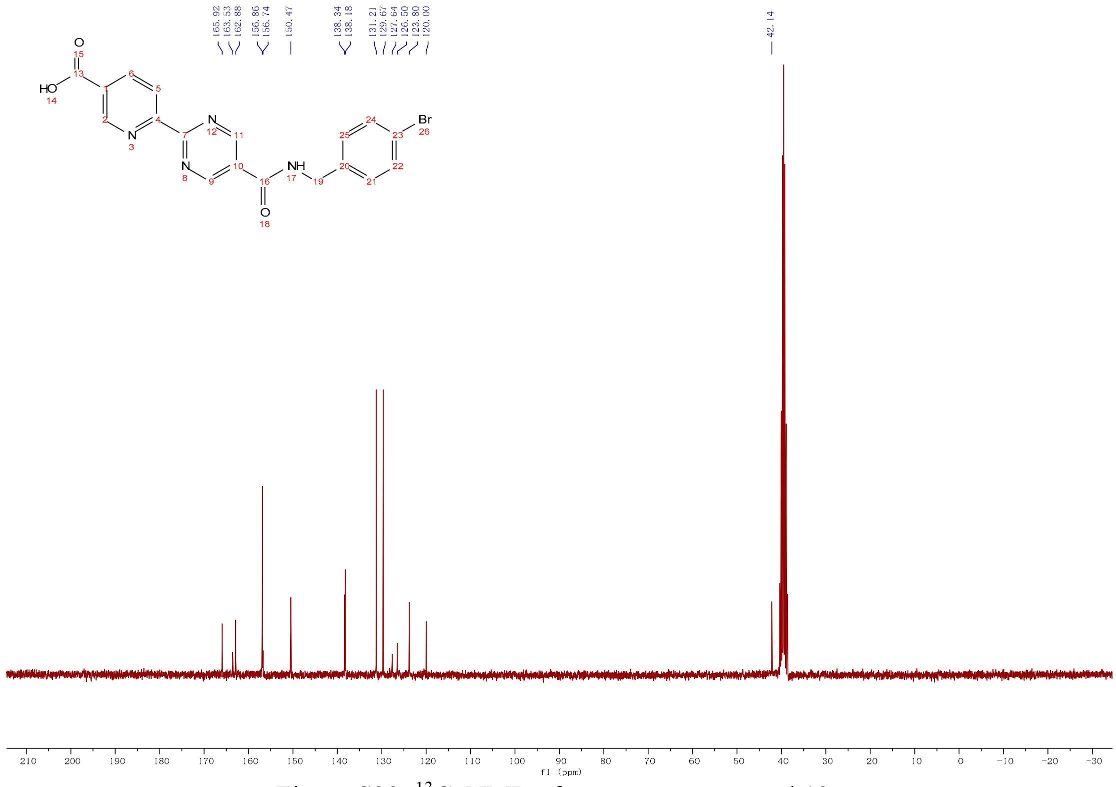

Figure S29: ^{13}C -NMR of spectrum compound 13c

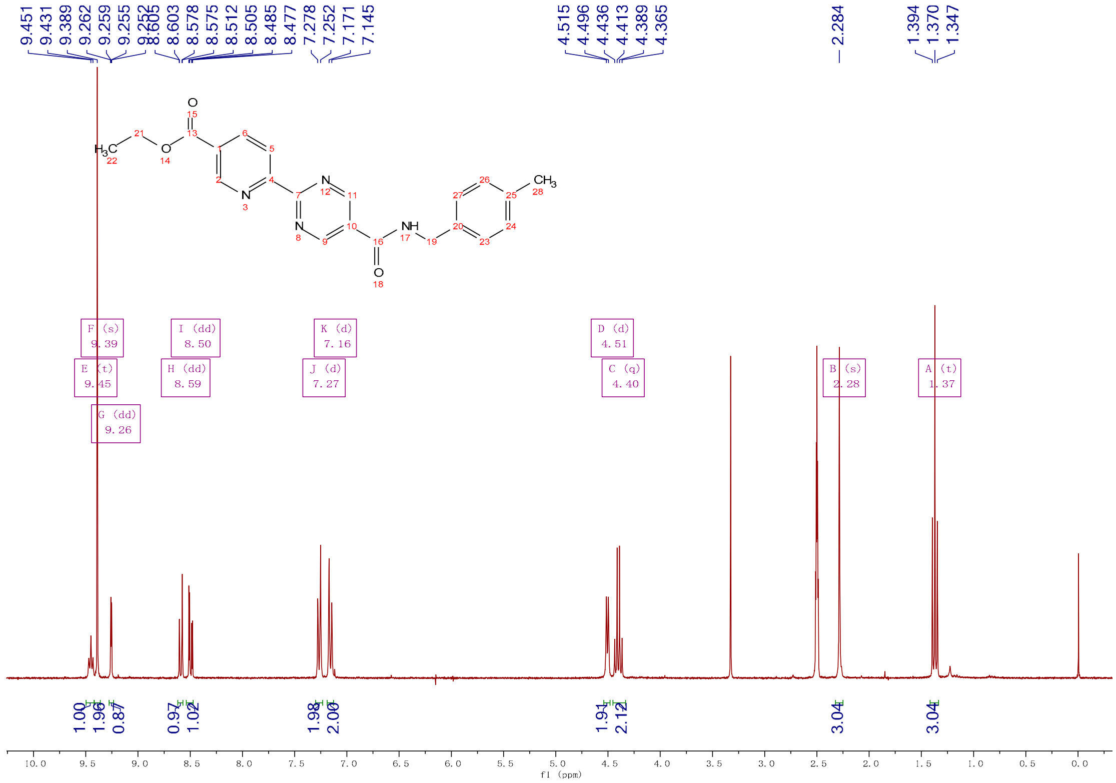

Figure S30: ^{1}H-NMR of spectrum compound 12d

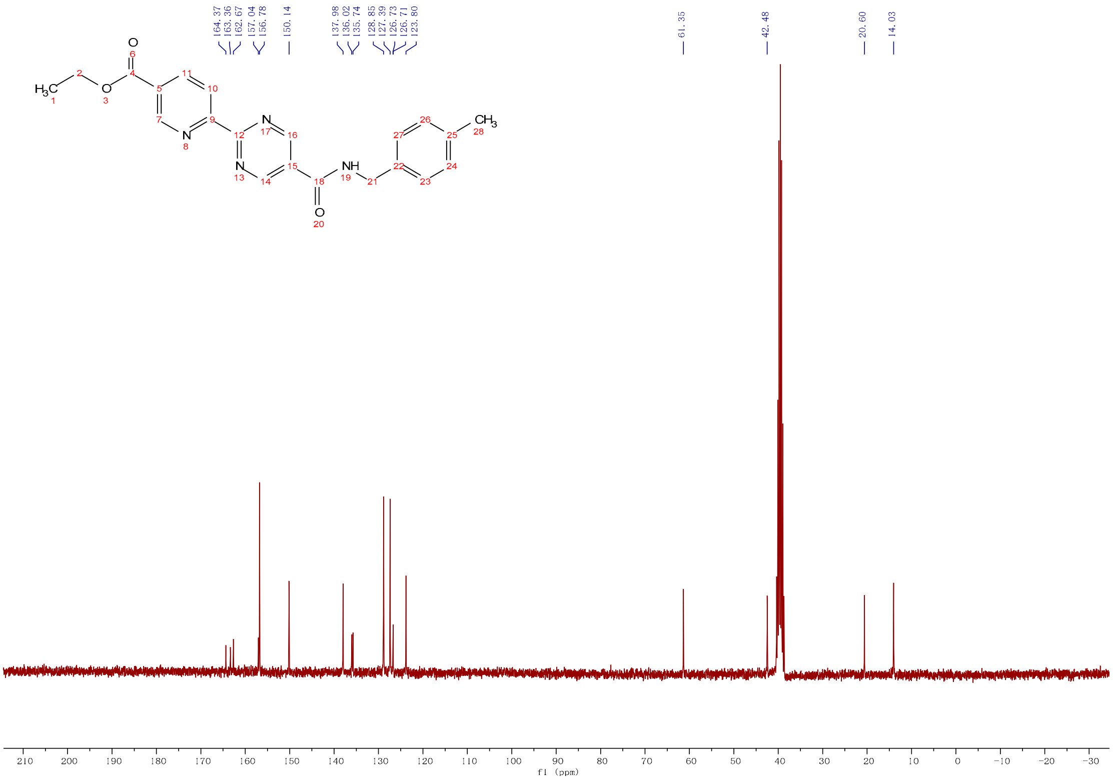

Figure S31: ^{13}C -NMR of spectrum compound 12d

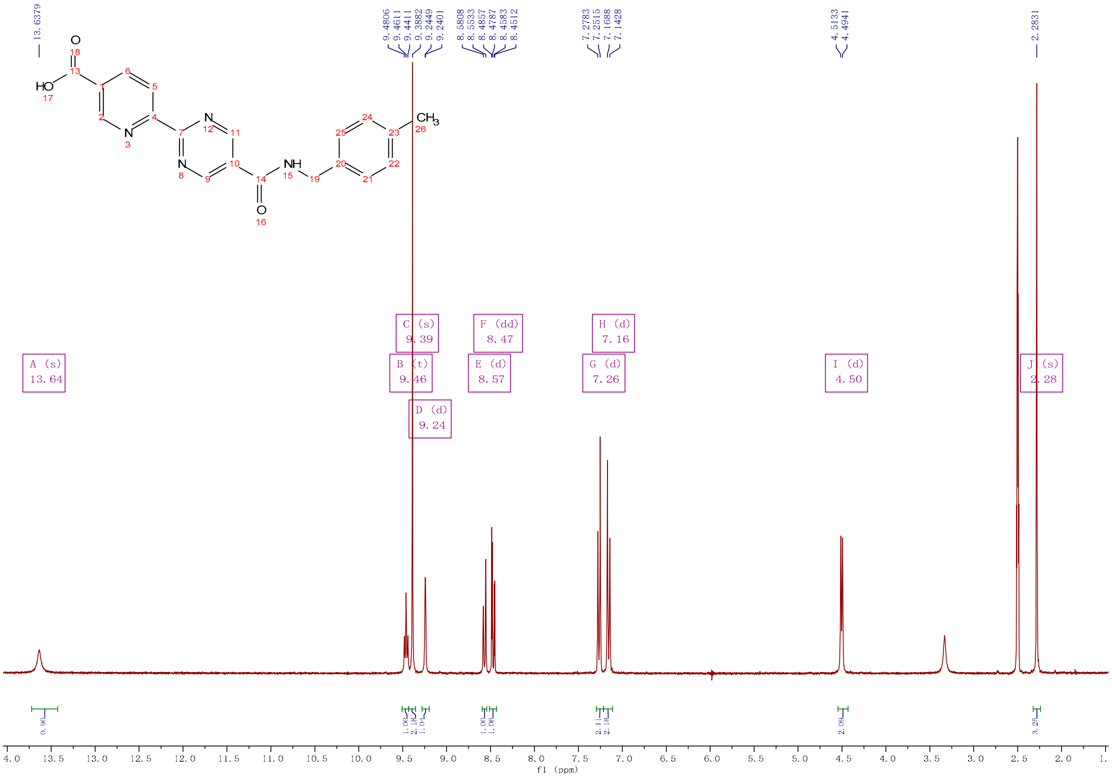

Figure S32: ^{1}H-NMR of spectrum compound 13d

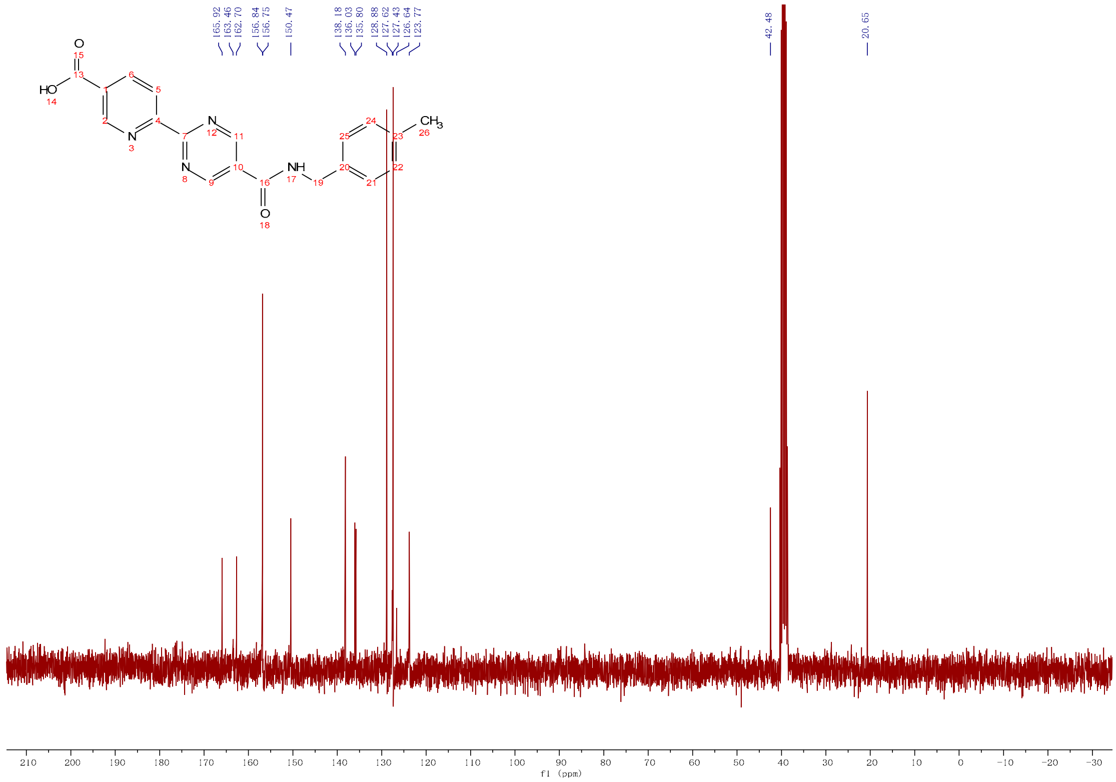

Figure S33: ^{13}C -NMR of spectrum compound 13d

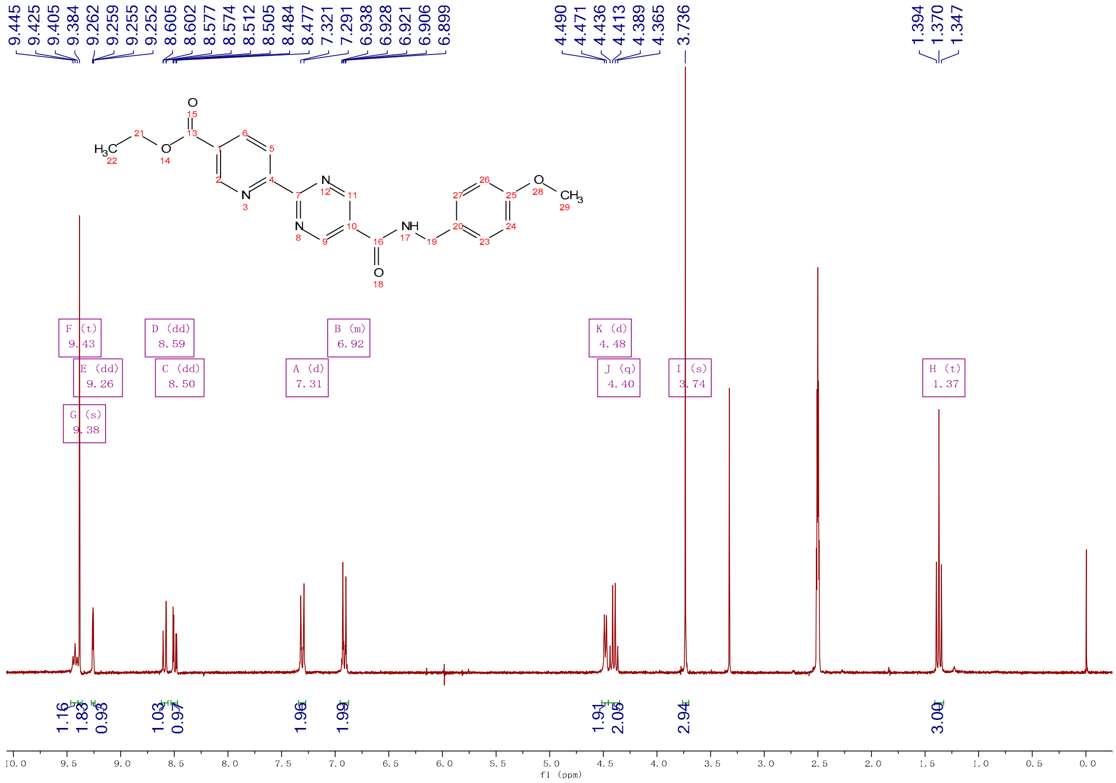

Figure S34: ^{1}H-NMR of spectrum compound 12e

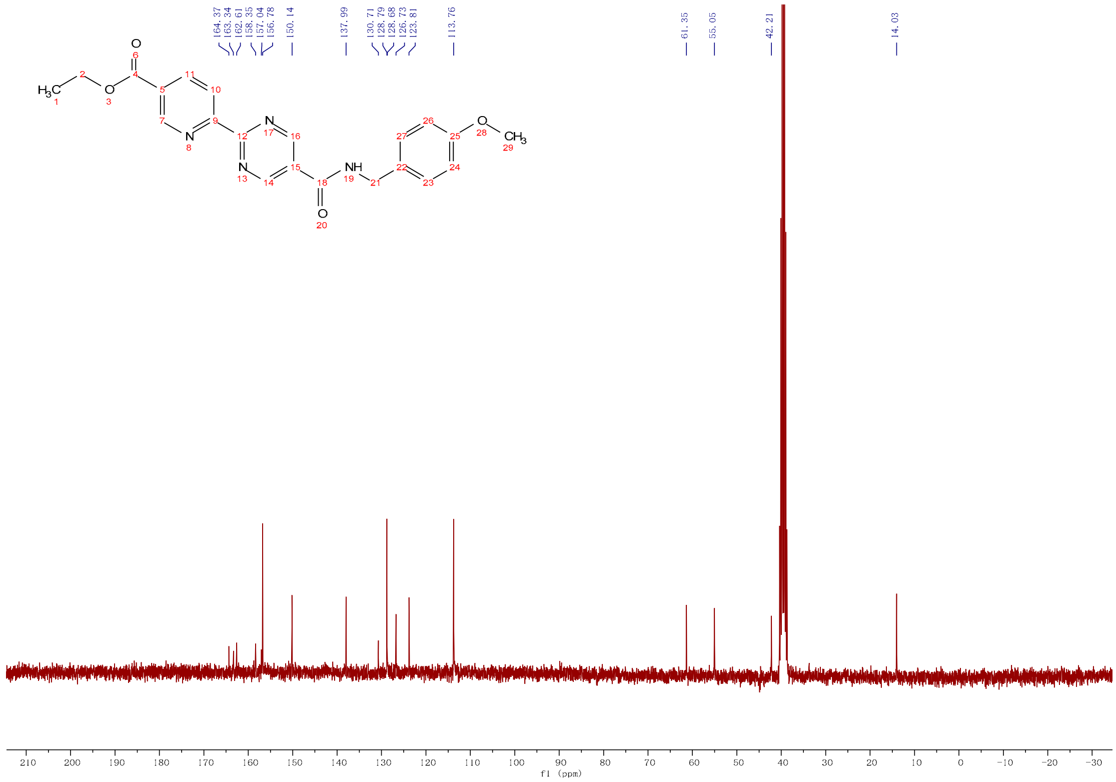

Figure S35: ^{13}C -NMR of spectrum compound 12e

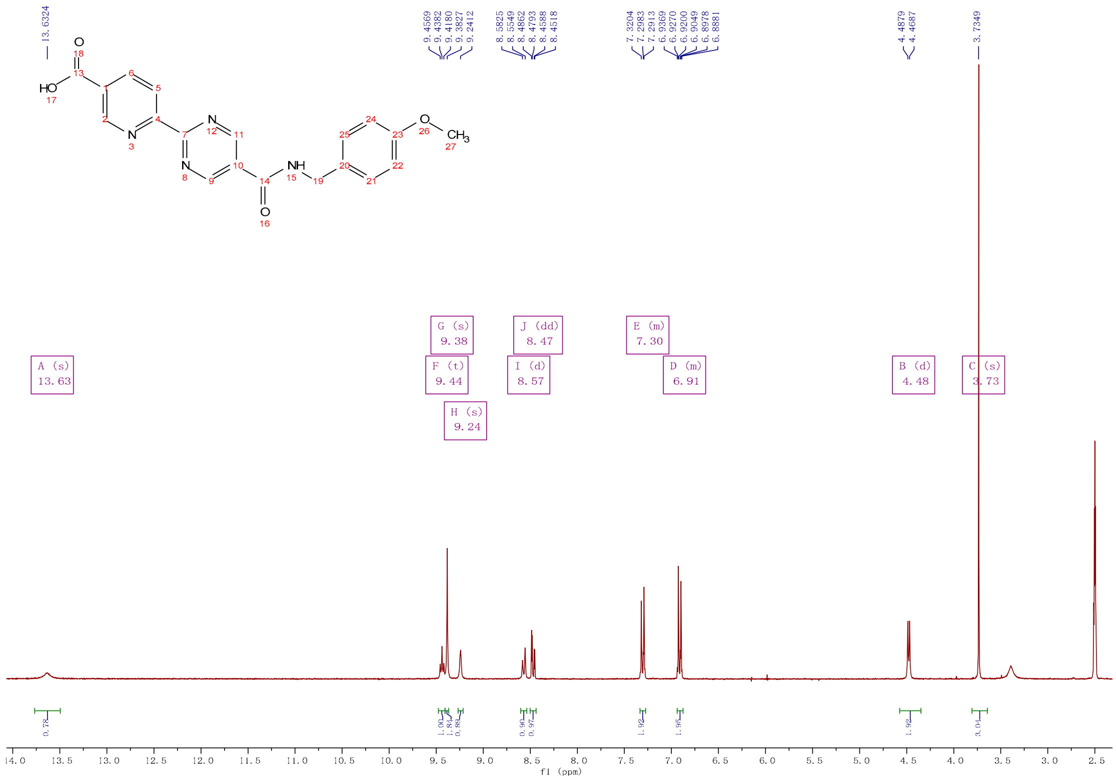

Figure S36: ^{1}H-NMR of spectrum compound 13e

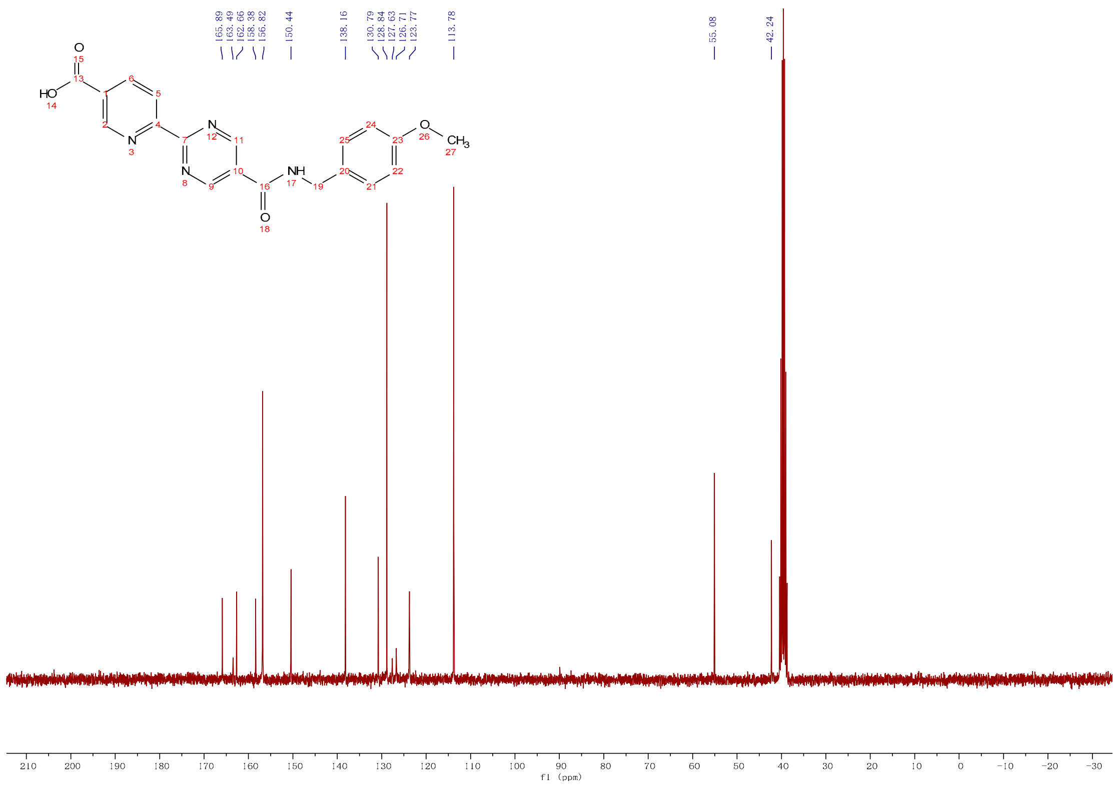

Figure S37: ^{13}C -NMR of spectrum compound 13e

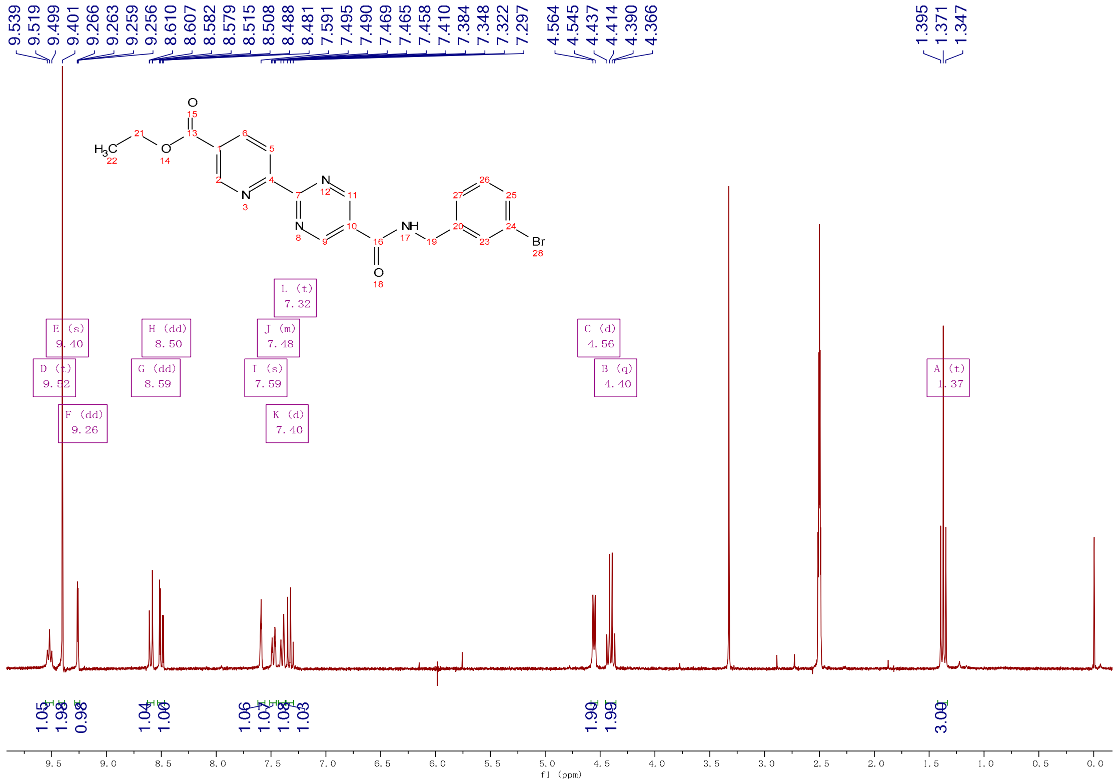

Figure S38: ^{1}H-NMR of spectrum compound 12f

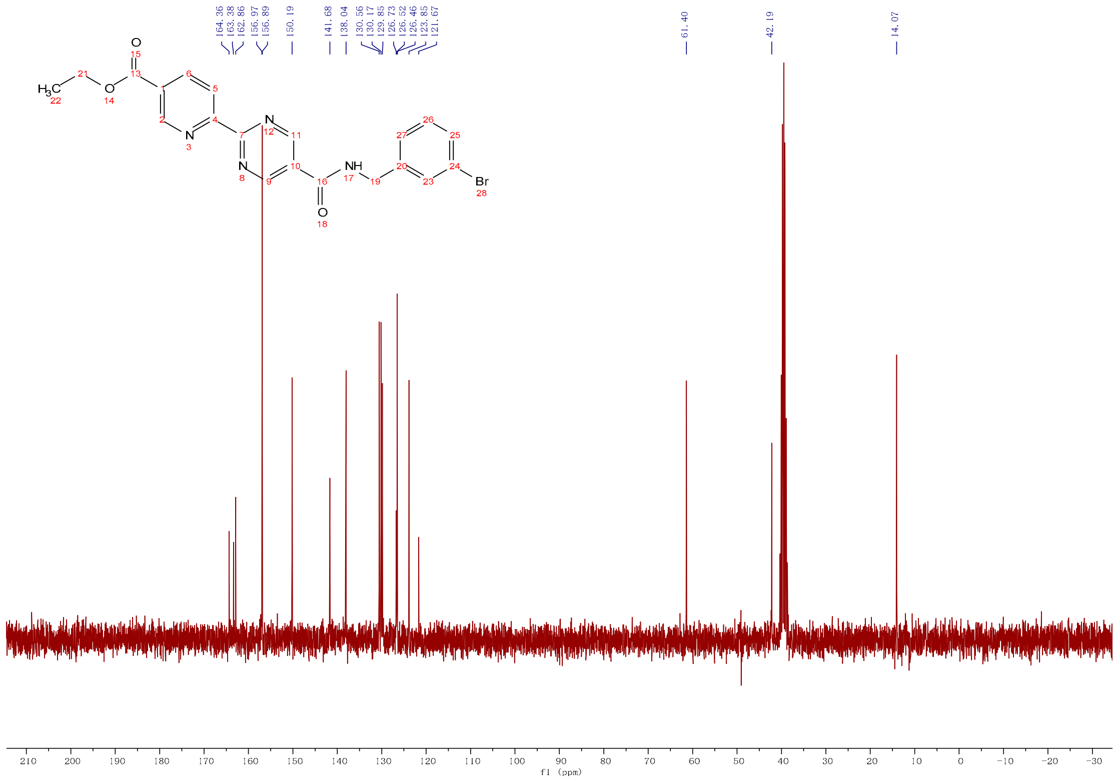

Figure S39: ^{13}C -NMR of spectrum compound 12f

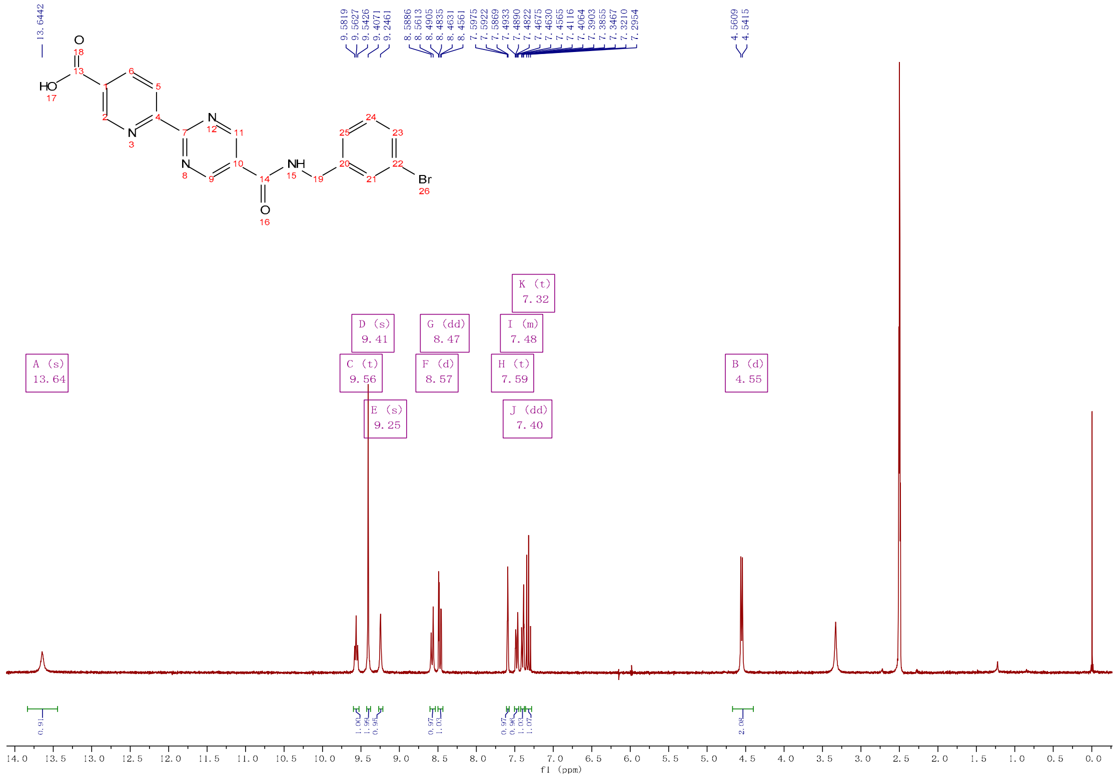

Figure S40: ^{1} H-NMR of spectrum compound 13f

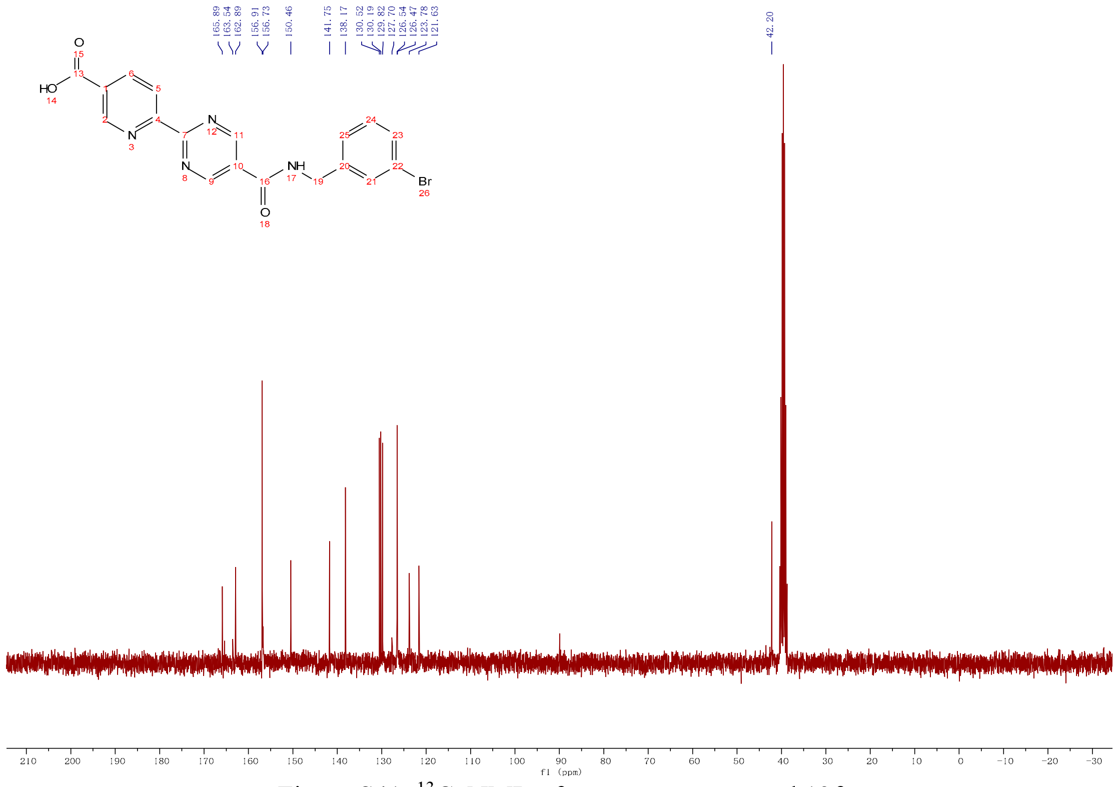

Figure S41: ^{13}C -NMR of spectrum compound 13f

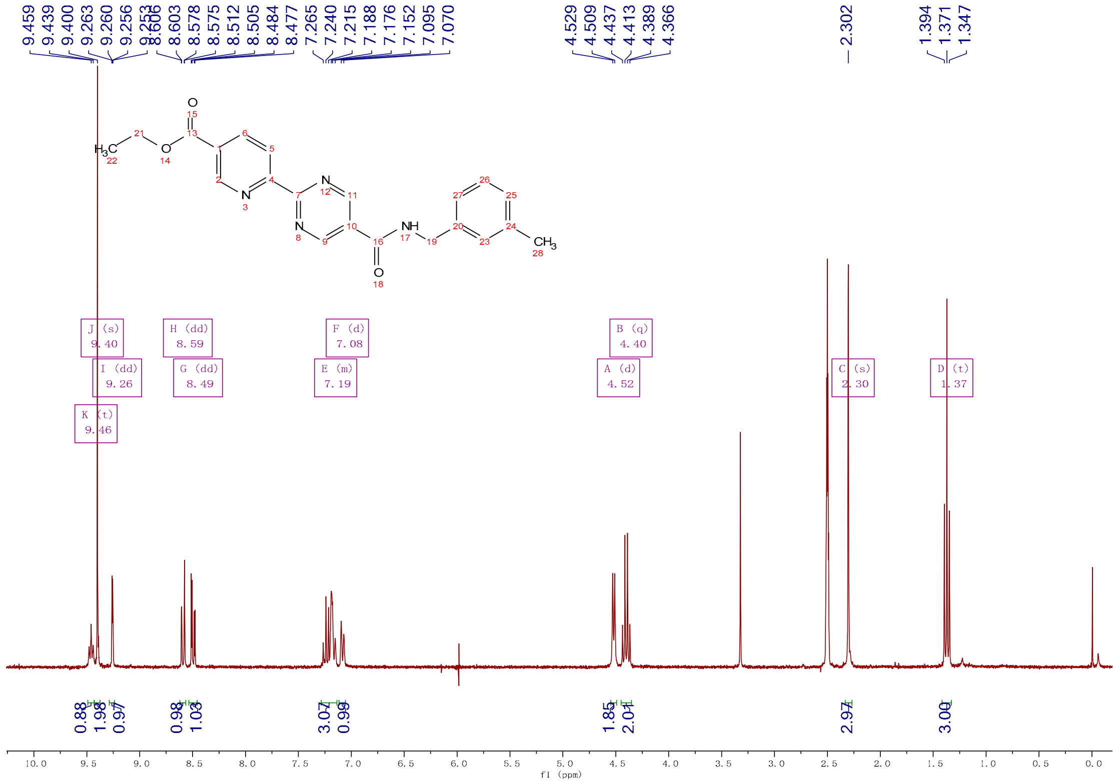

Figure S42: ^{1} H-NMR of spectrum compound 12g

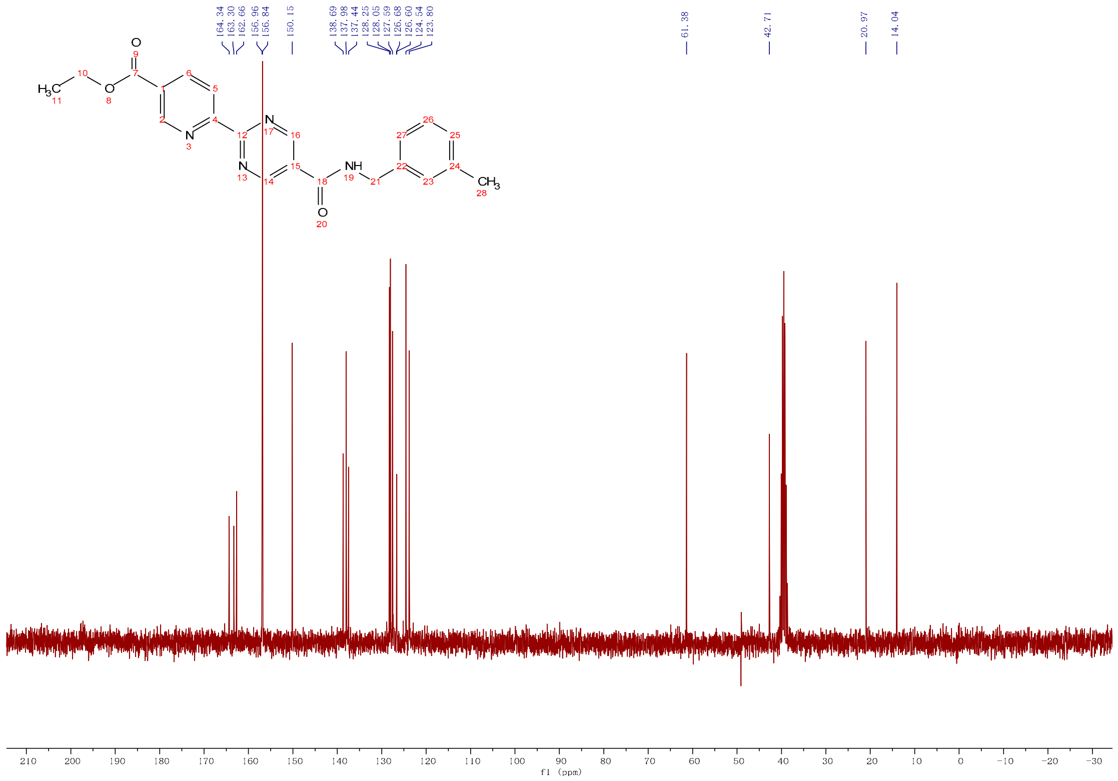

Figure S43: ^{13}C -NMR of spectrum compound 12g

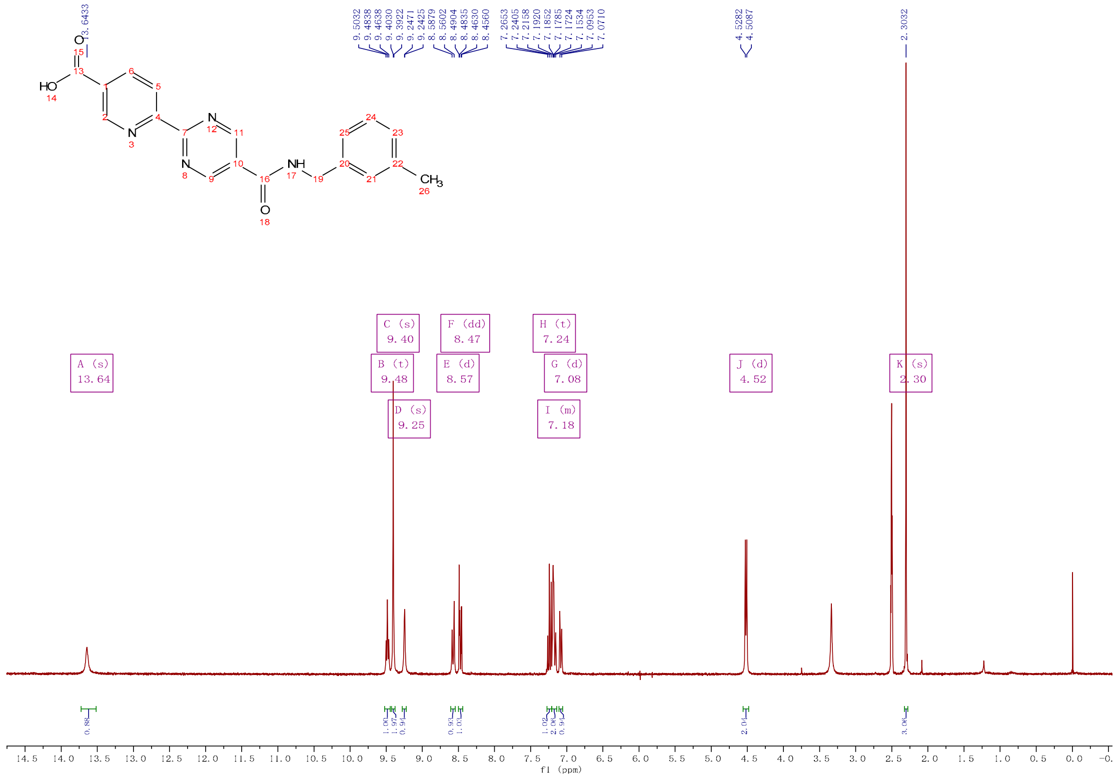

Figure S44: ^{1}H-NMR of spectrum compound 13g

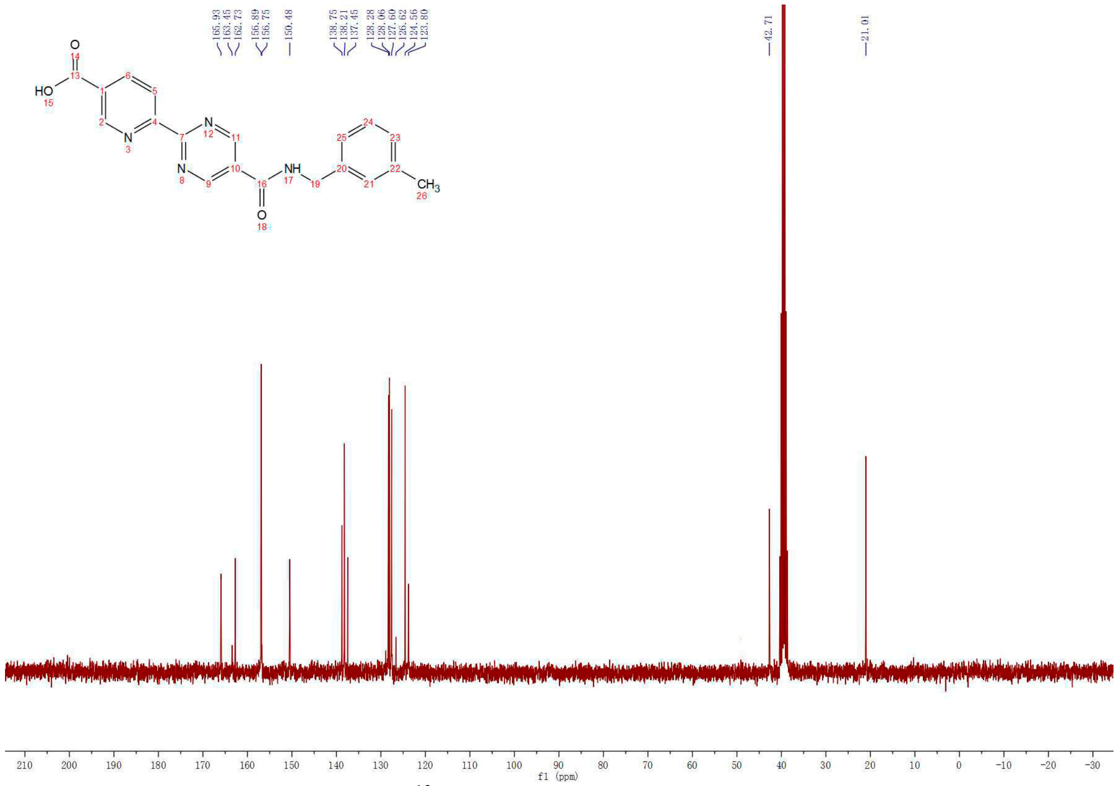

Figure S45: ^{13}C -NMR of spectrum compound 13g

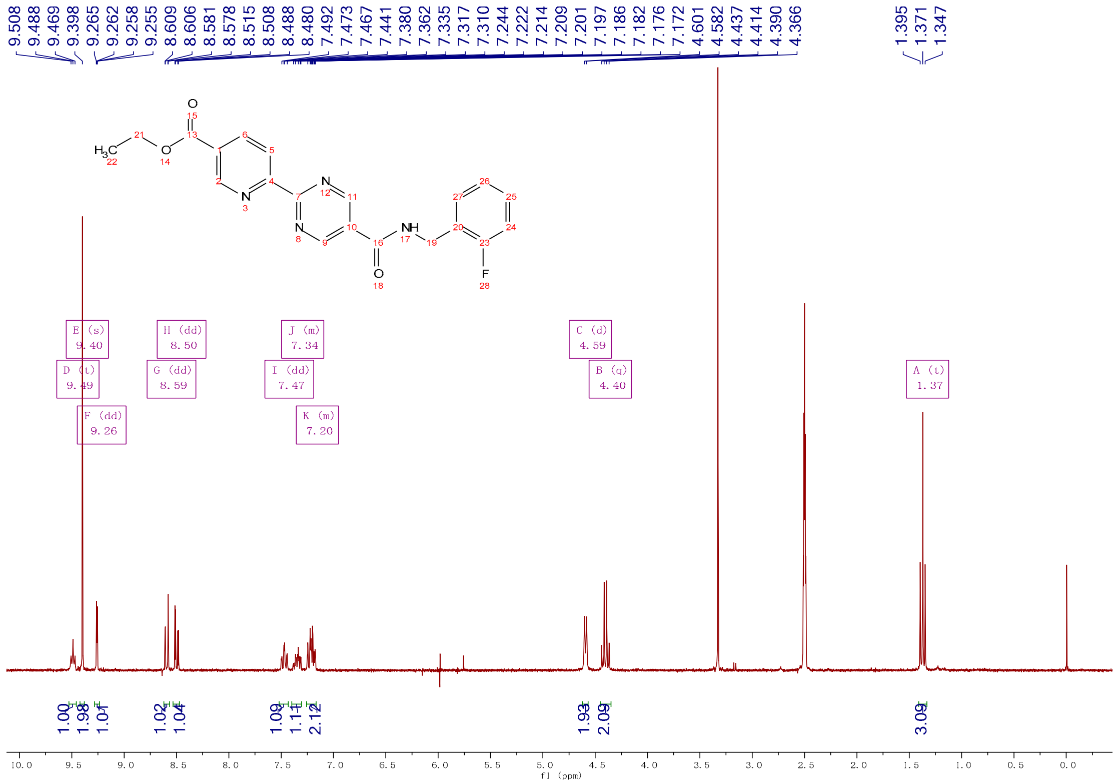

Figure S46: ^{1} H-NMR of spectrum compound 12h

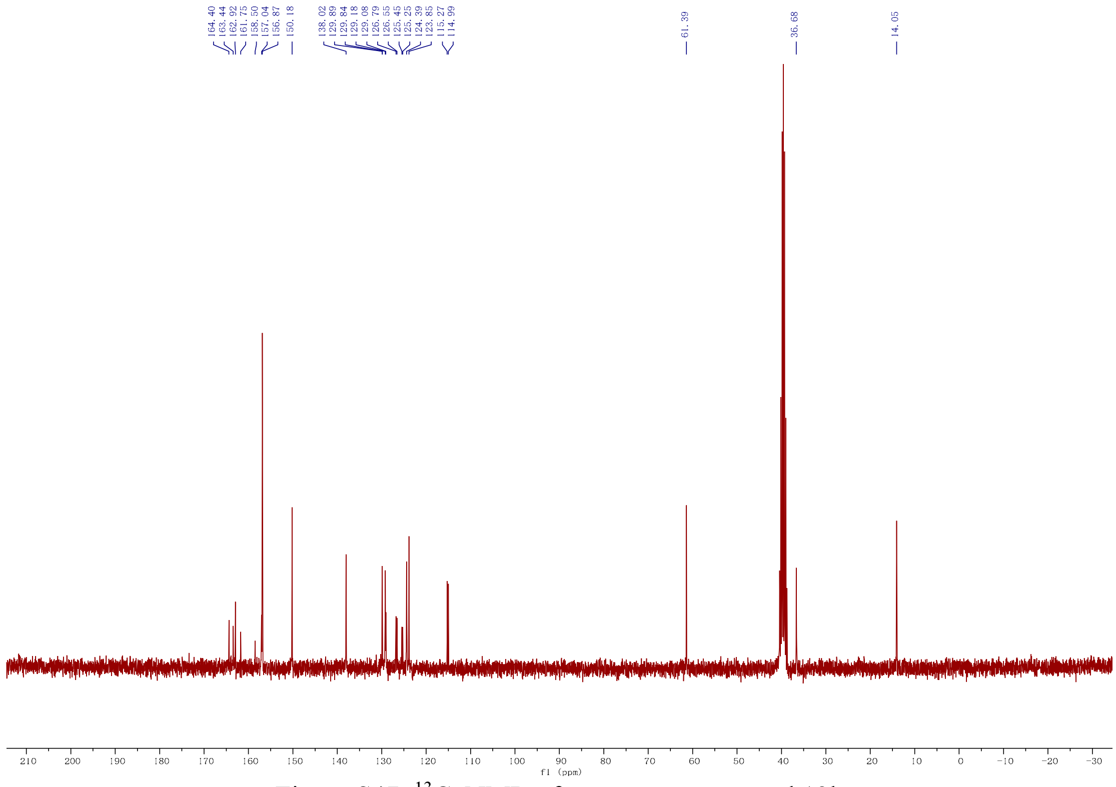

Figure S47: ^{13}C -NMR of spectrum compound 12h

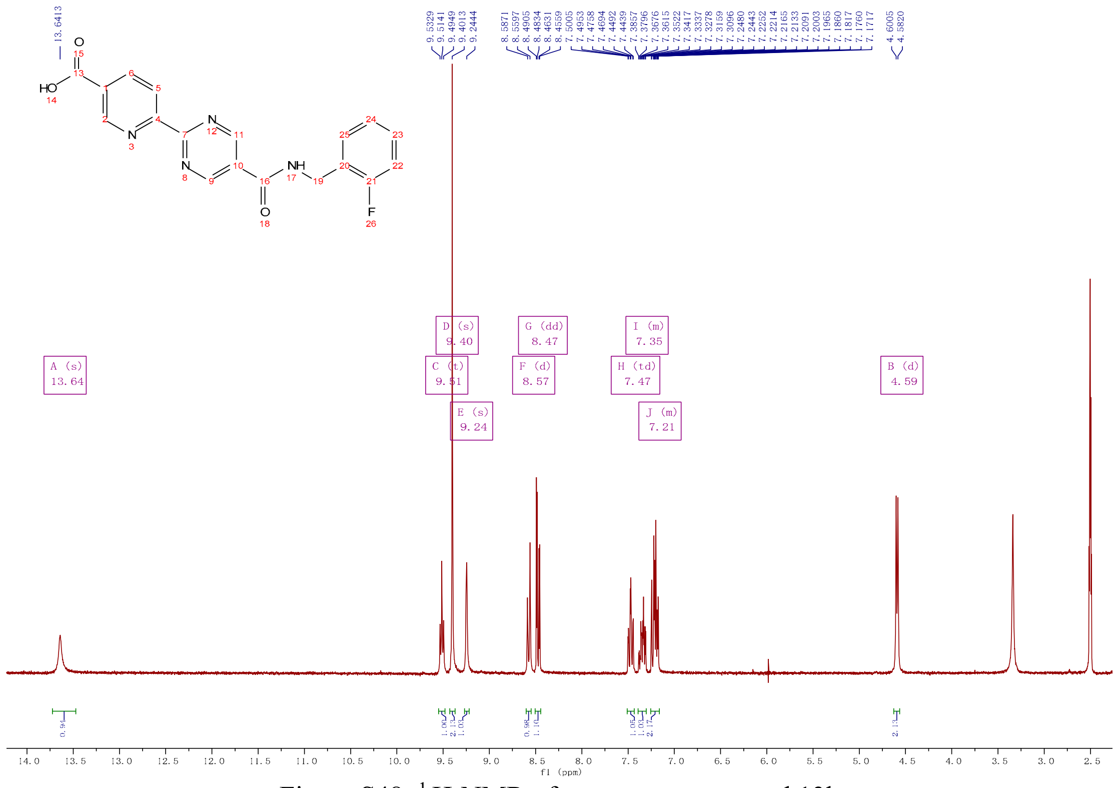

Figure S48: ^{1}H-NMR of spectrum compound 13h

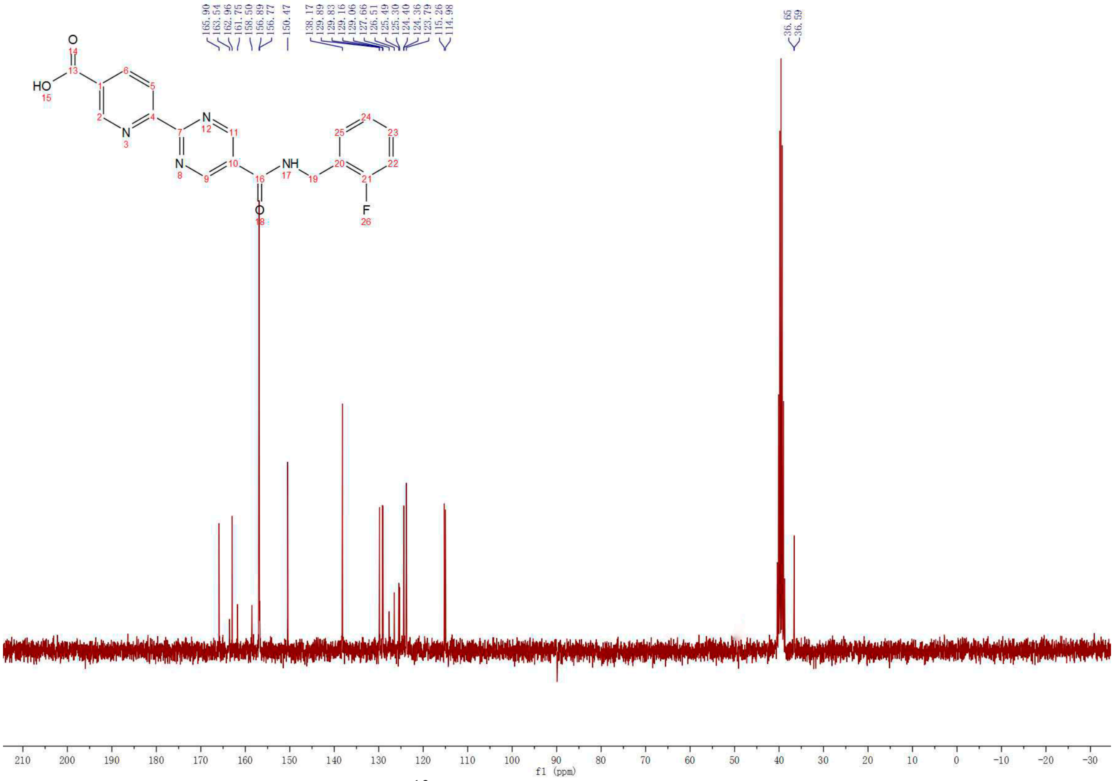

Figure S49: ^{13}C -NMR of spectrum compound 13h

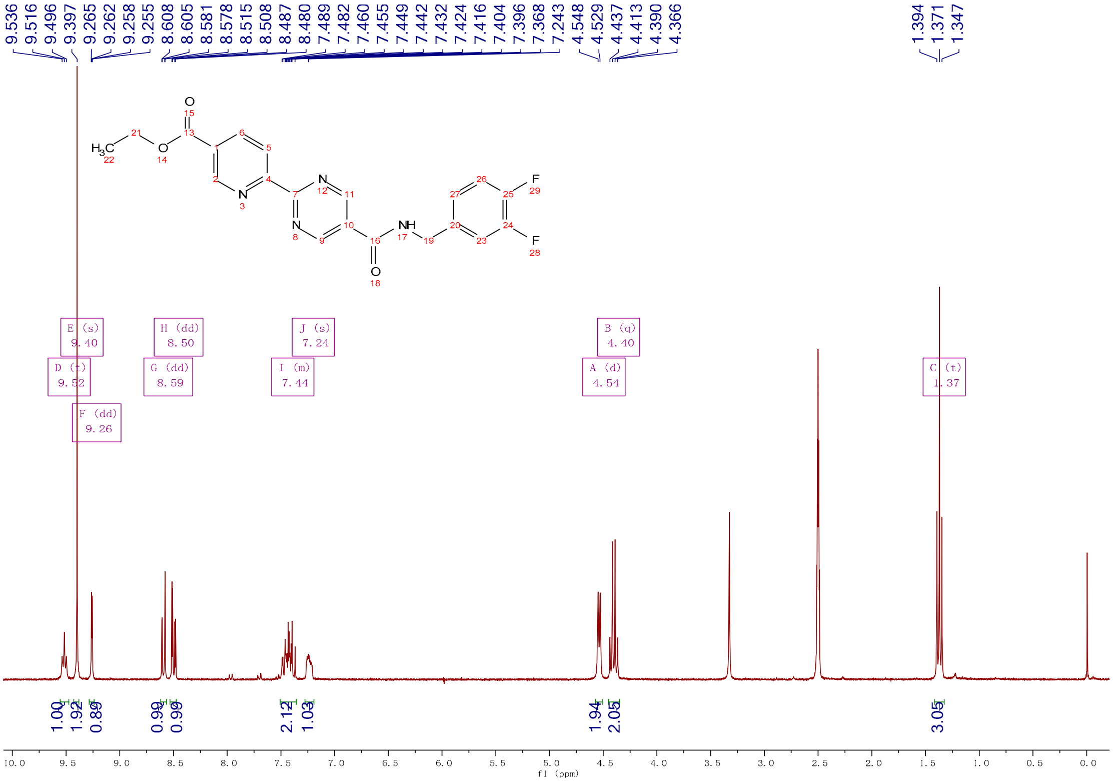

Figure S50: ^{1}H-NMR of spectrum compound 12i

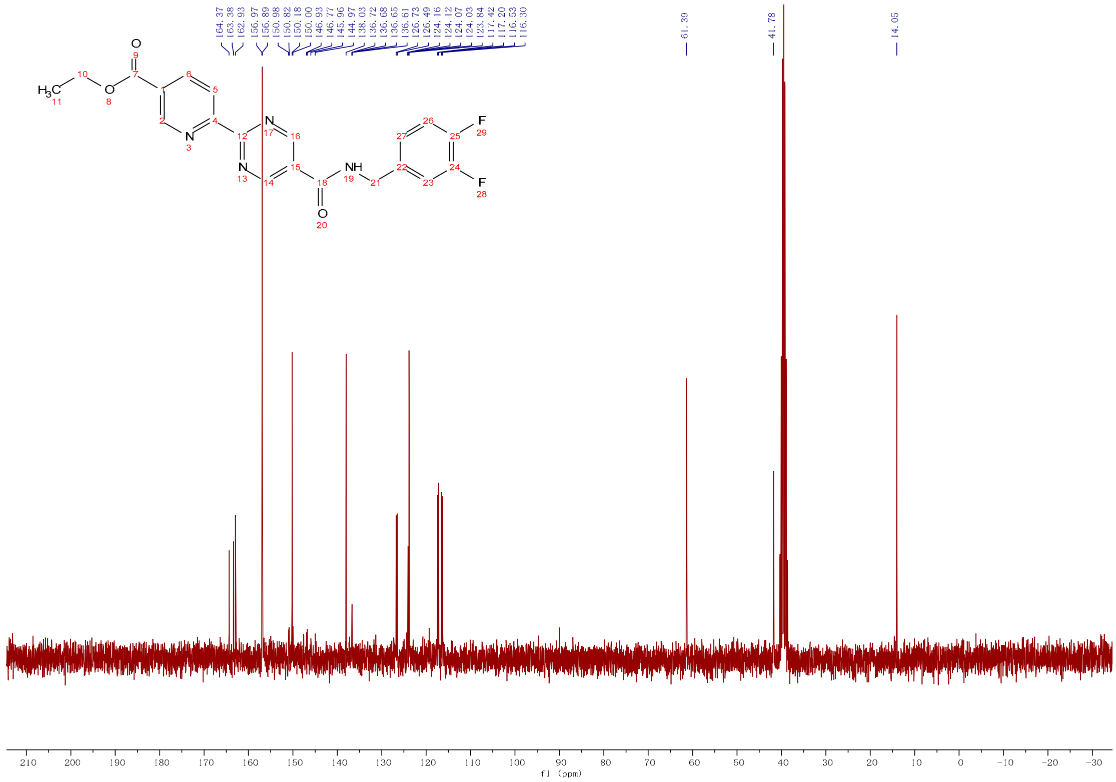

Figure S51: ^{13}C -NMR of spectrum compound 12i

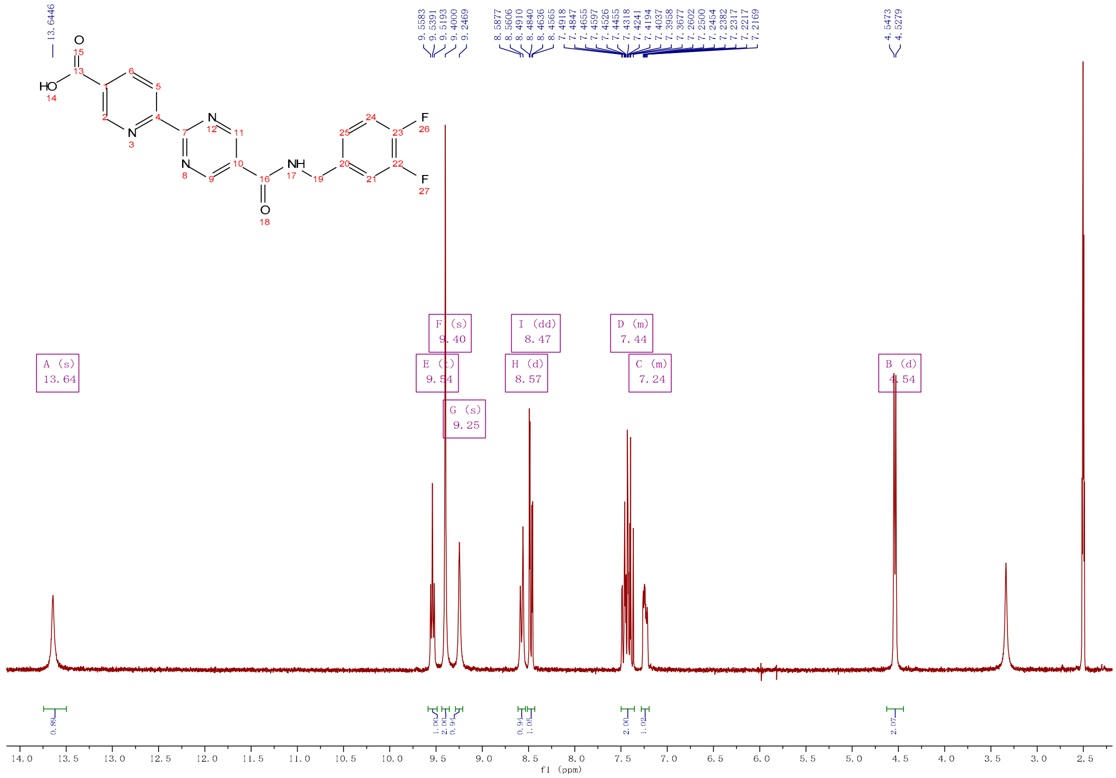

Figure S52: ^{1} H-NMR of spectrum compound 13i

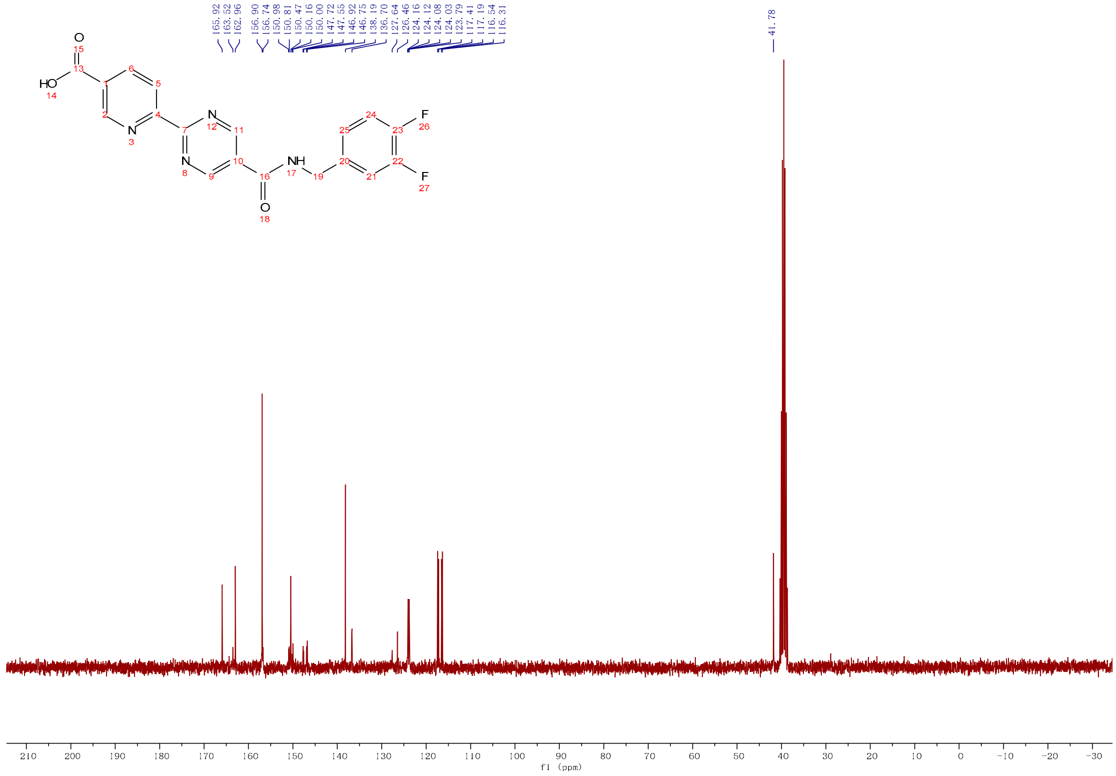

Figure S53: ^{13}C -NMR of spectrum compound 13i

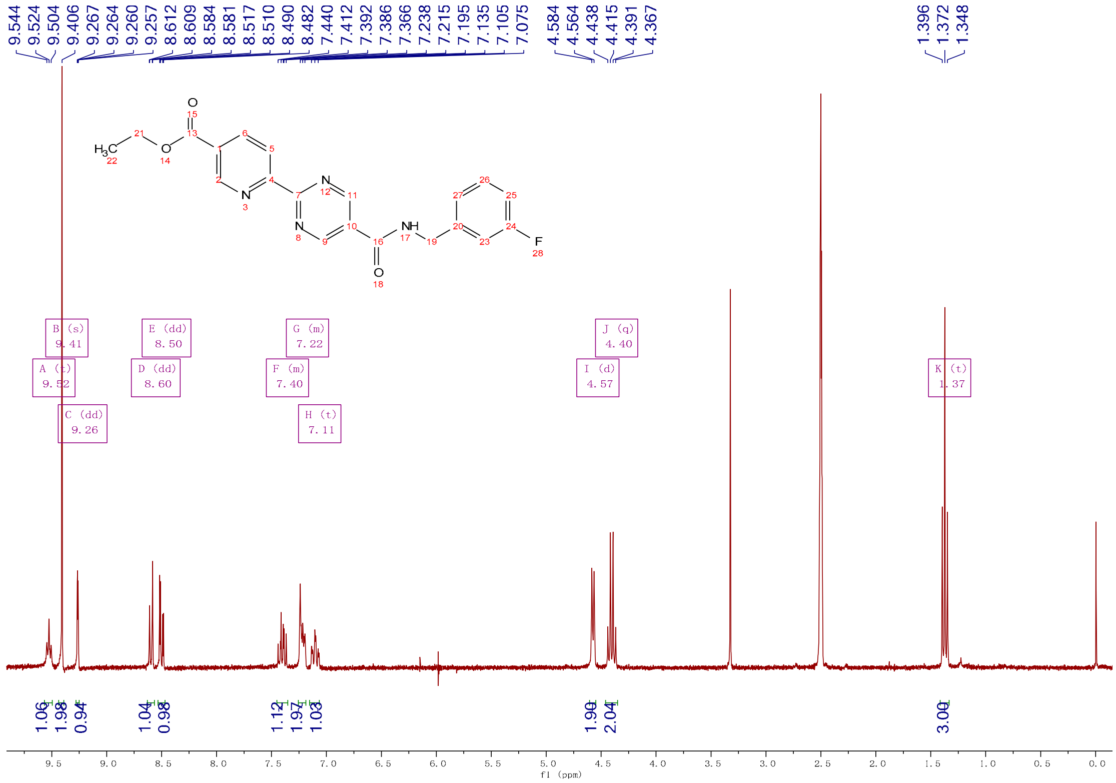

Figure S54: ^{1} H-NMR of spectrum compound 12j

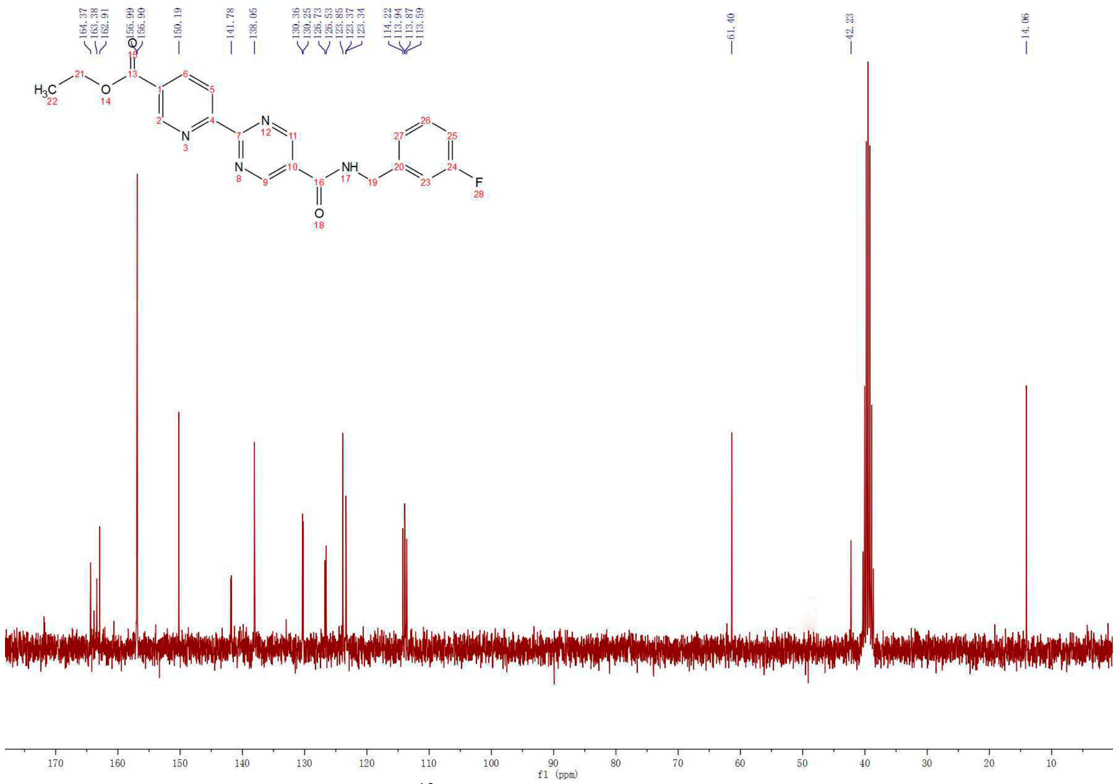

Figure S55: ^{13}C-NMR of spectrum compound 12j

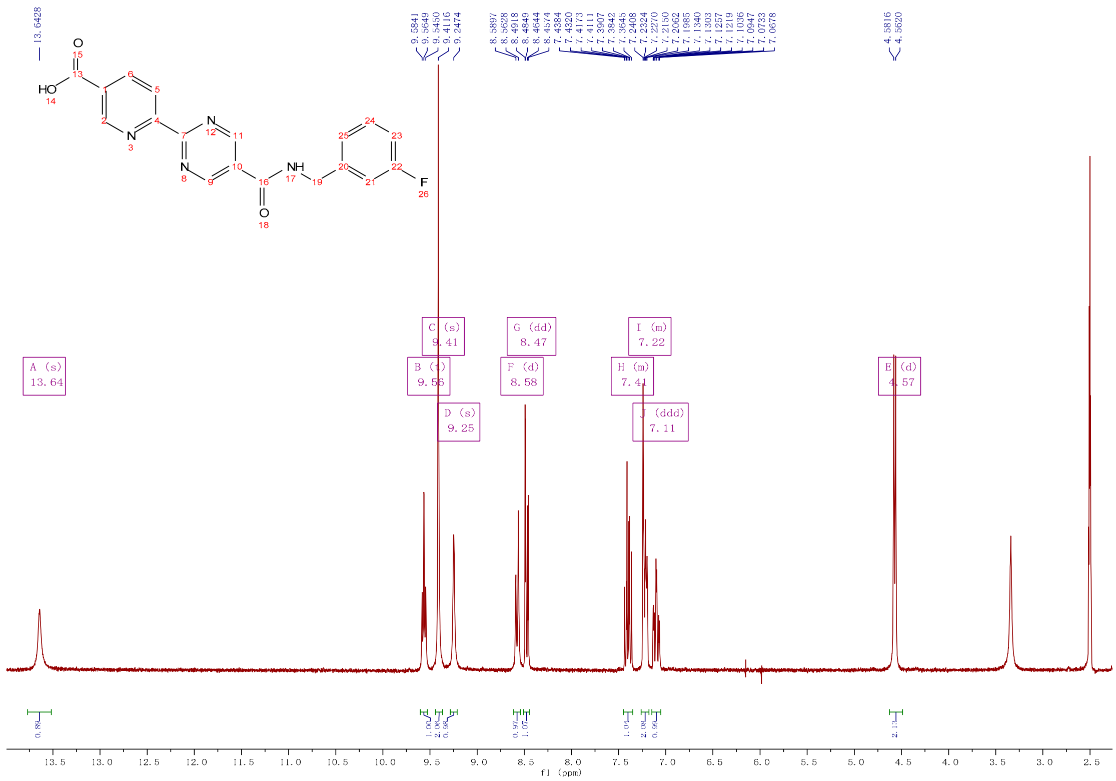

Figure S56: ^{1}H-NMR of spectrum compound 13j

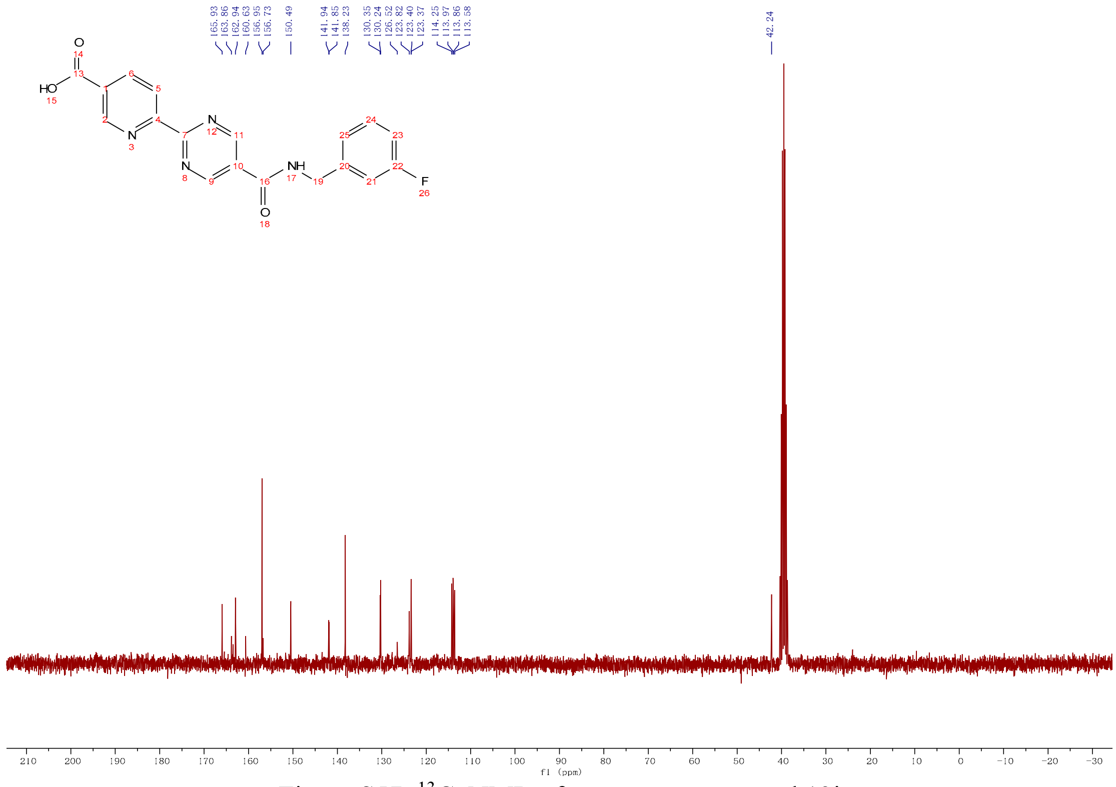

Figure S57: ^{13}C -NMR of spectrum compound 13j

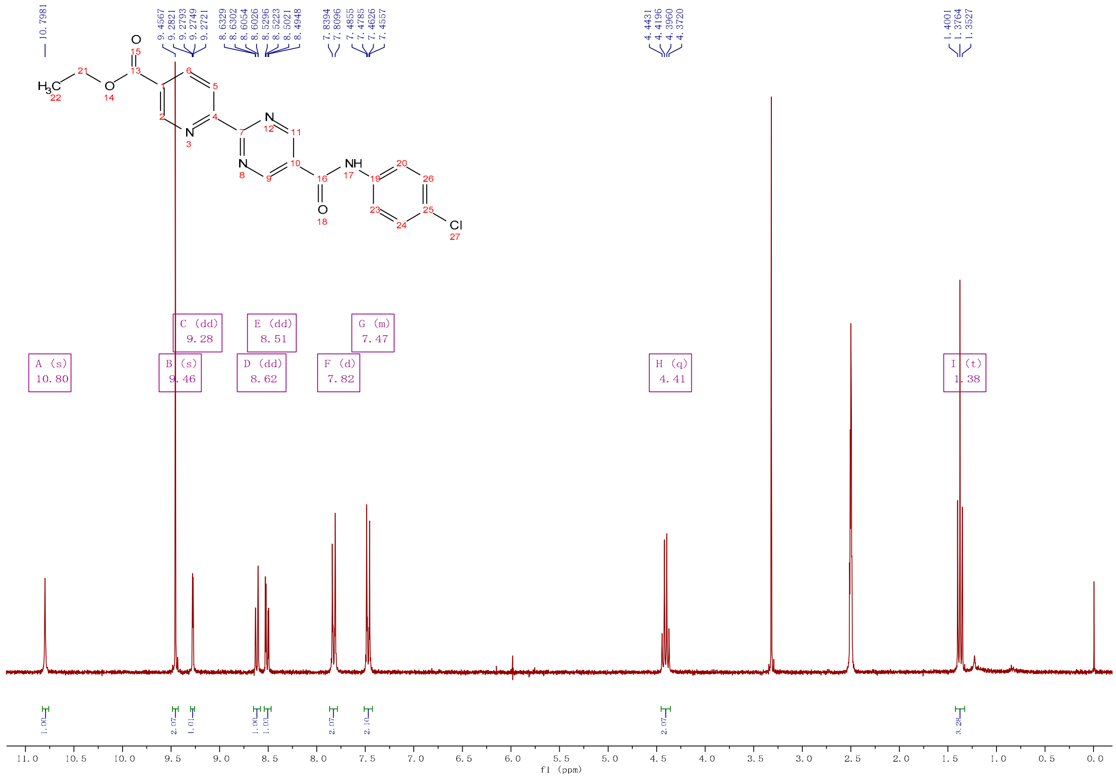

Figure S58: ^{1} H-NMR of spectrum compound 12k

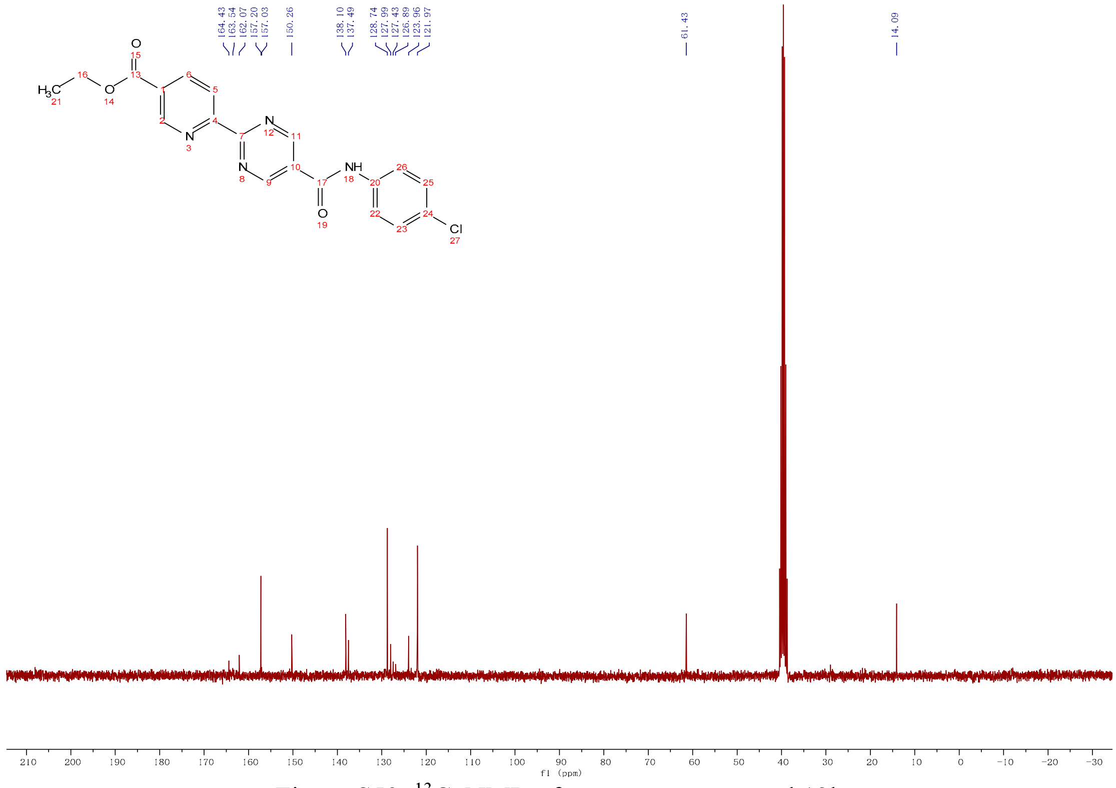

Figure S59: ^{13}C -NMR of spectrum compound 12k

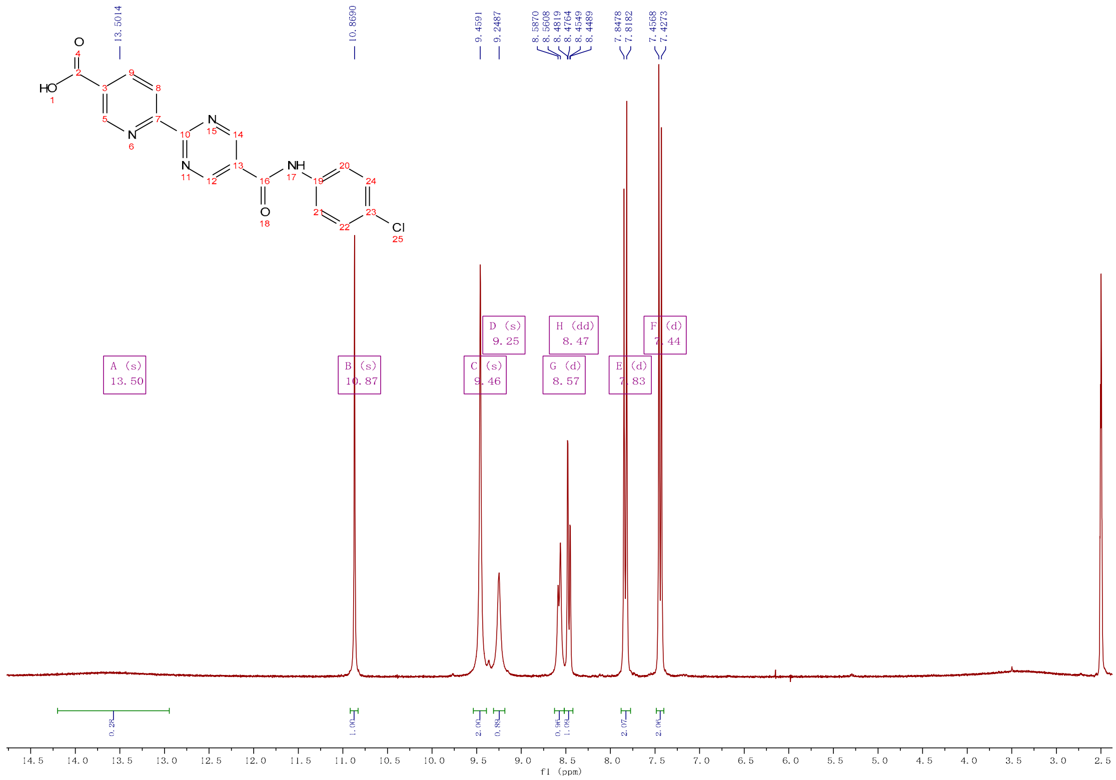

Figure S60: ^{1}H-NMR of spectrum compound 13k

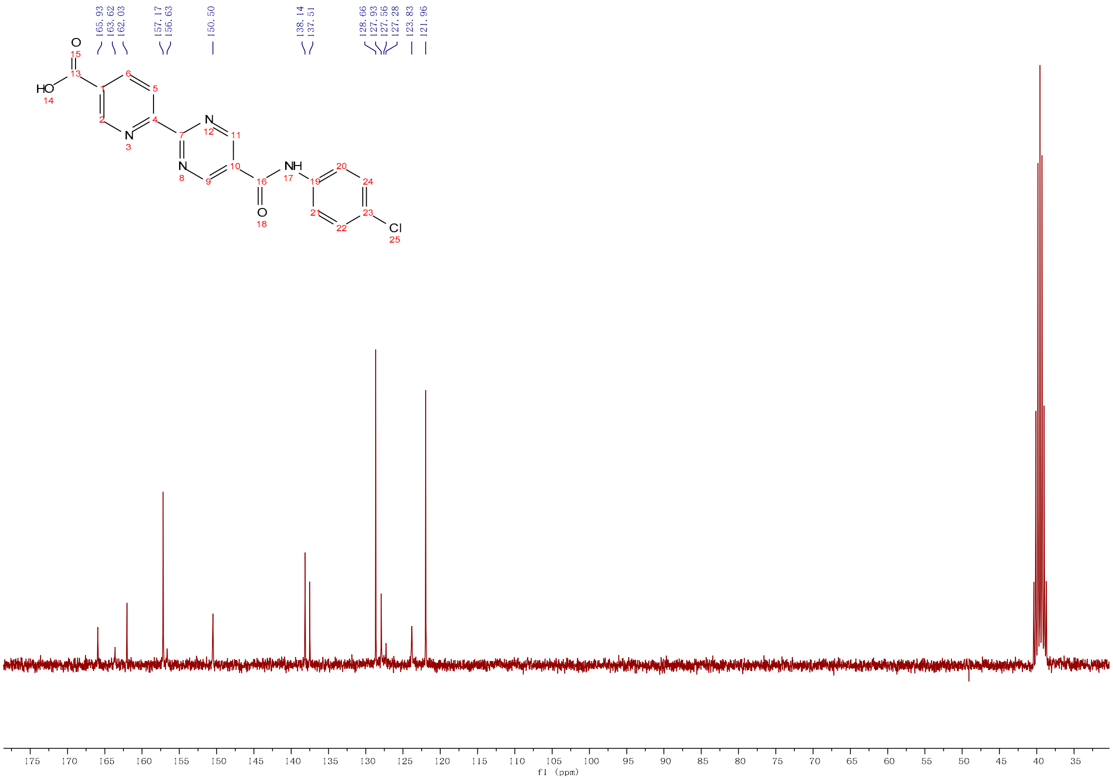

Figure S61: ^{13}C -NMR of spectrum compound 13k

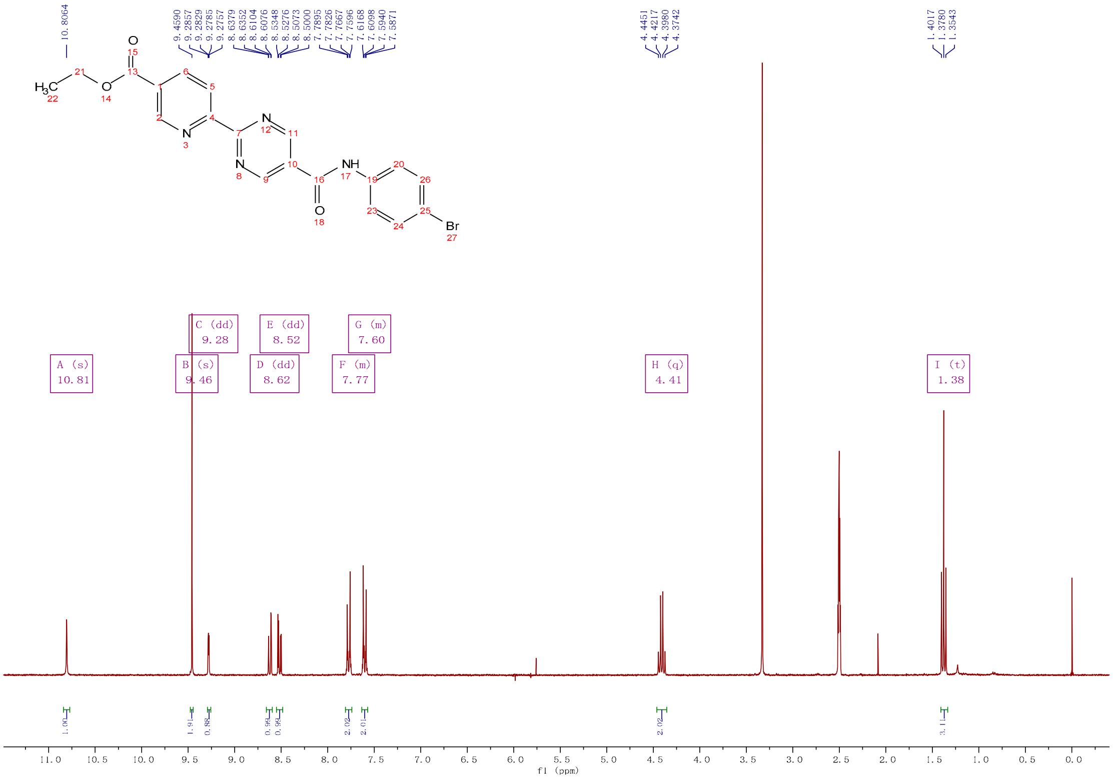

Figure S62: ^{1}H-NMR of spectrum compound 12l

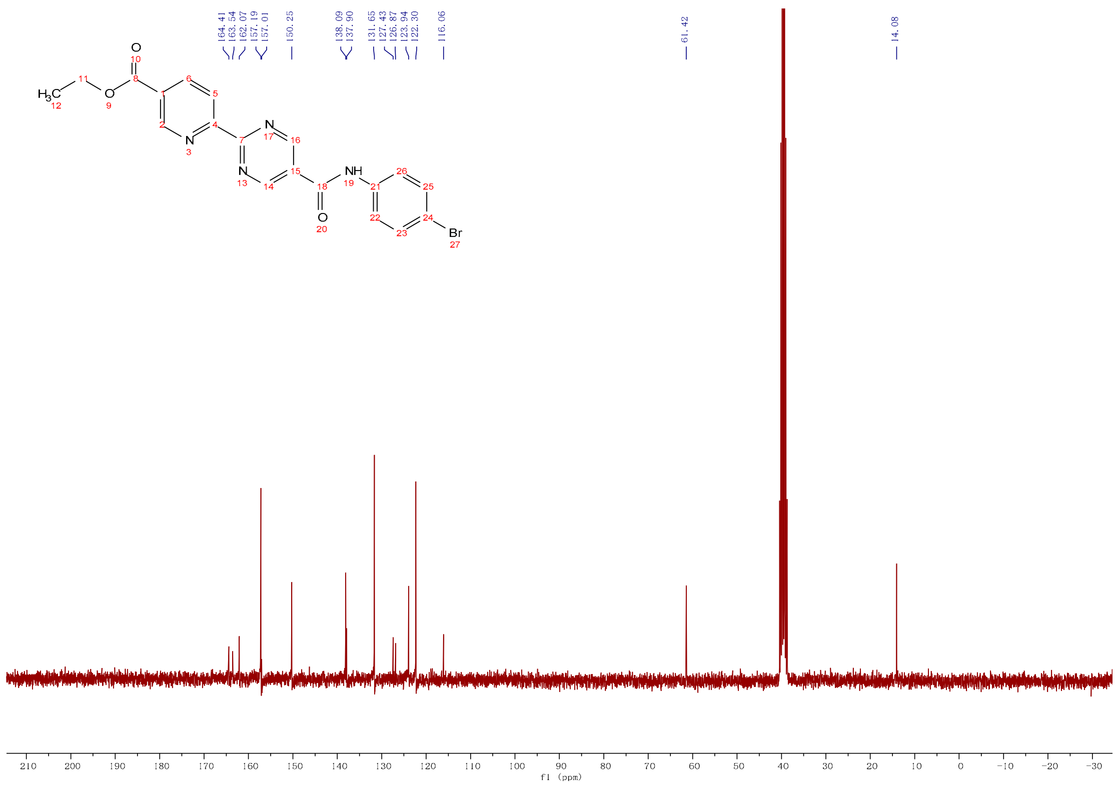

Figure S63: ^{13}C -NMR of spectrum compound 12l

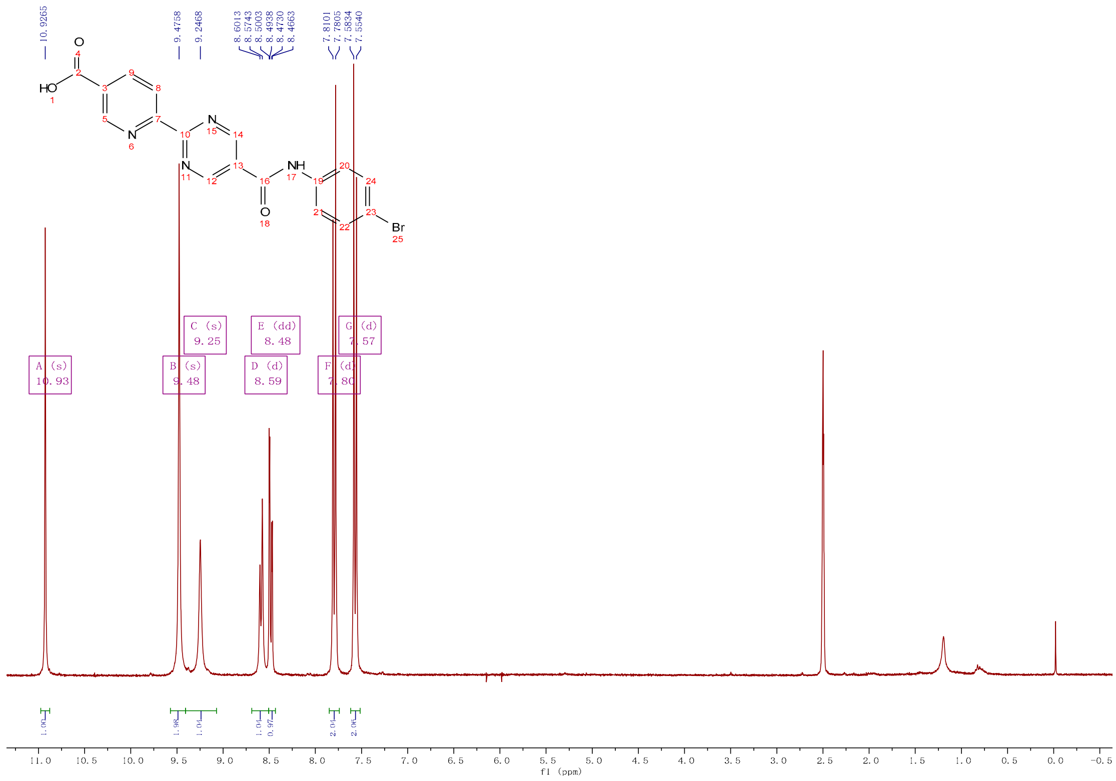

Figure S64: ^{1}H-NMR of spectrum compound 13l

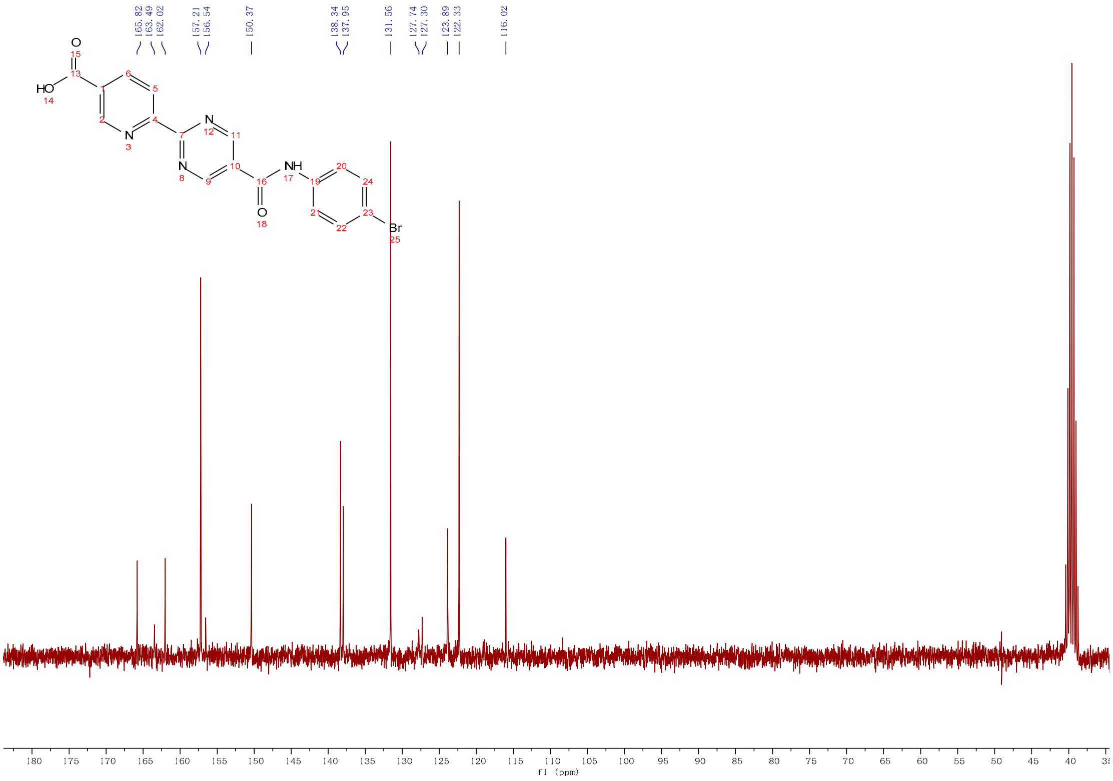

Figure S65: ^{13}C -NMR of spectrum compound 13l

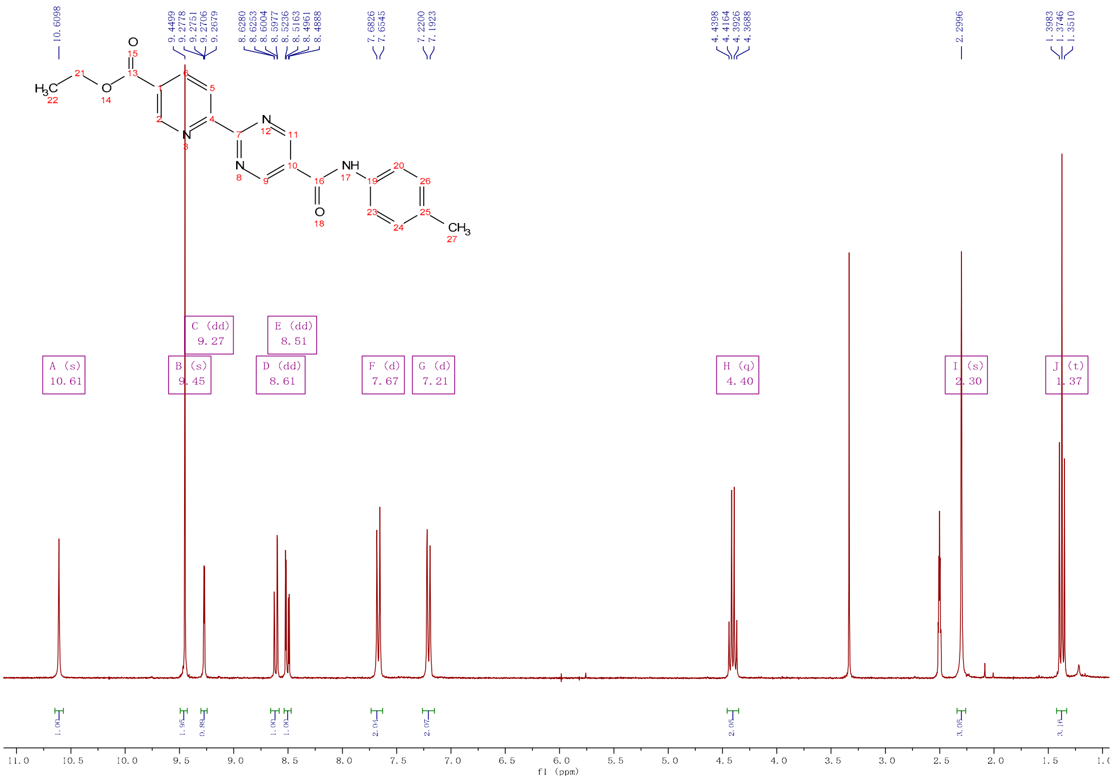

Figure S66: ^{1} H-NMR of spectrum compound 12m

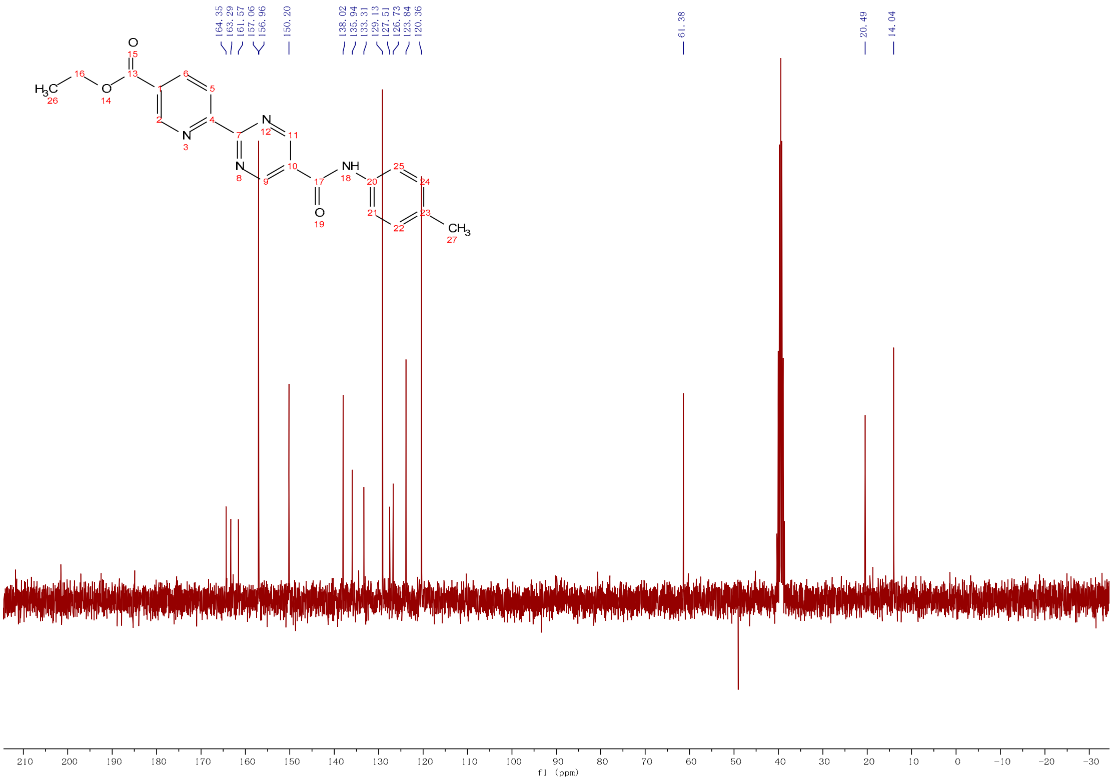

Figure S67: ^{13}C -NMR of spectrum compound 12m

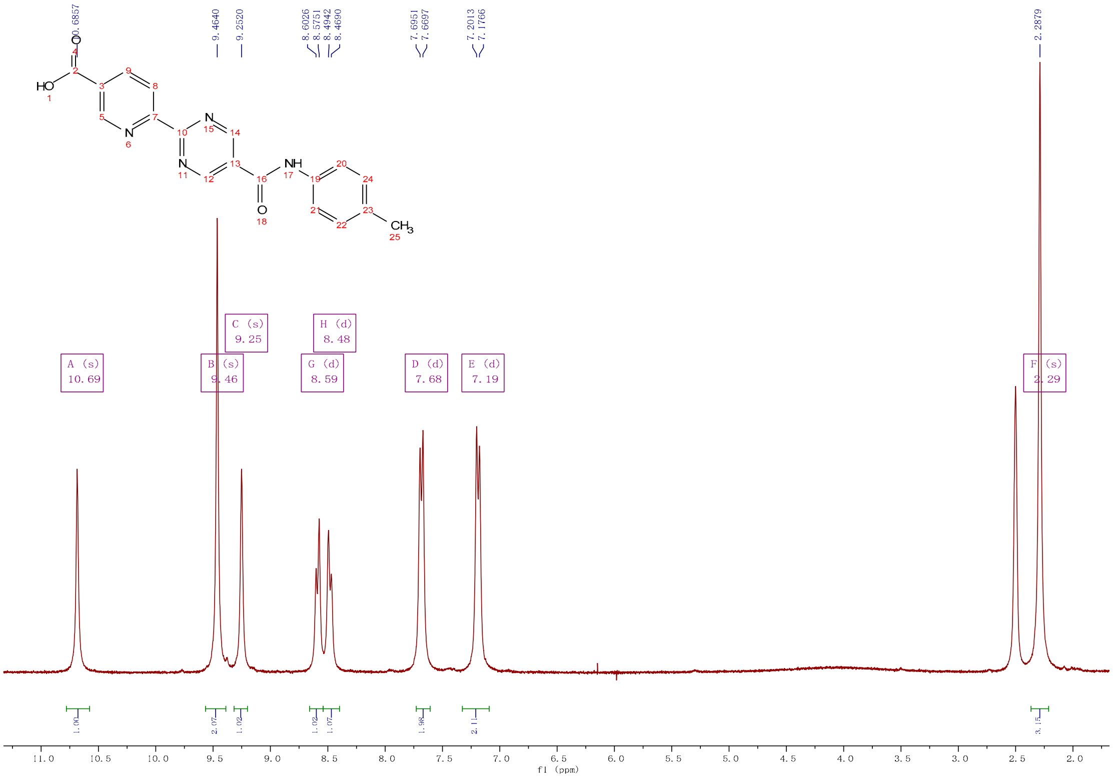

Figure S68: ^{1}H-NMR of spectrum compound 13m

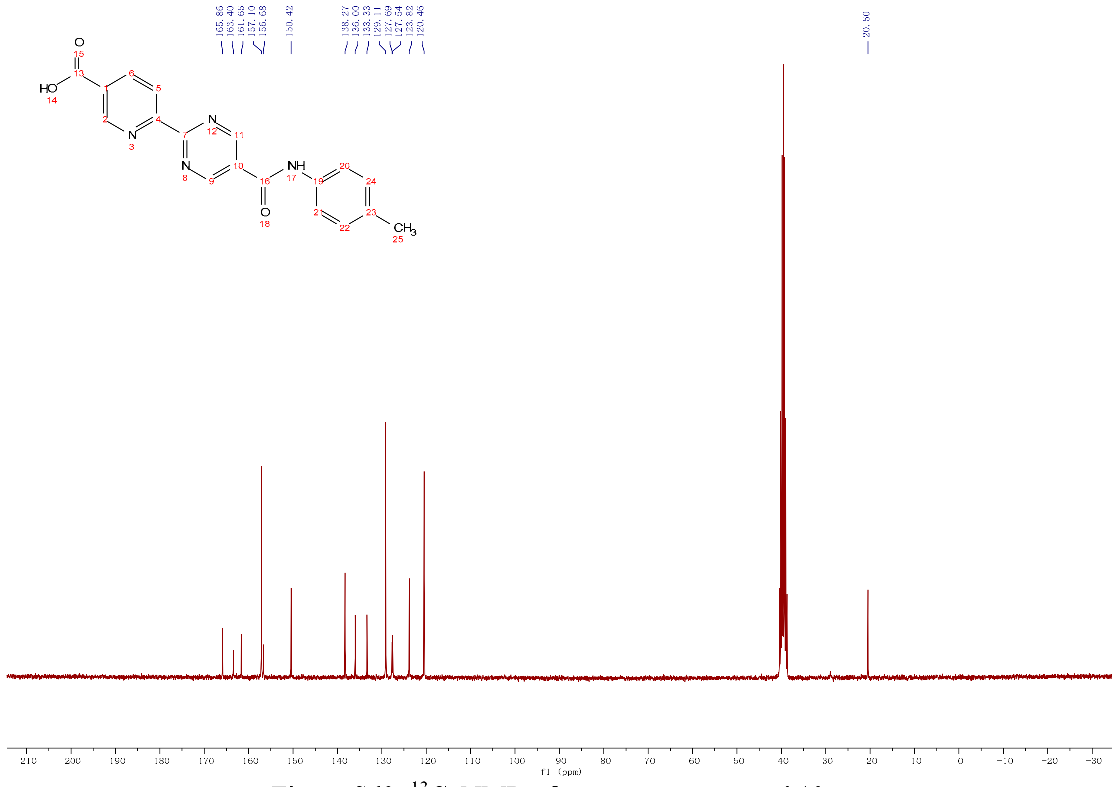

Figure S69: ^{13}C -NMR of spectrum compound 13m

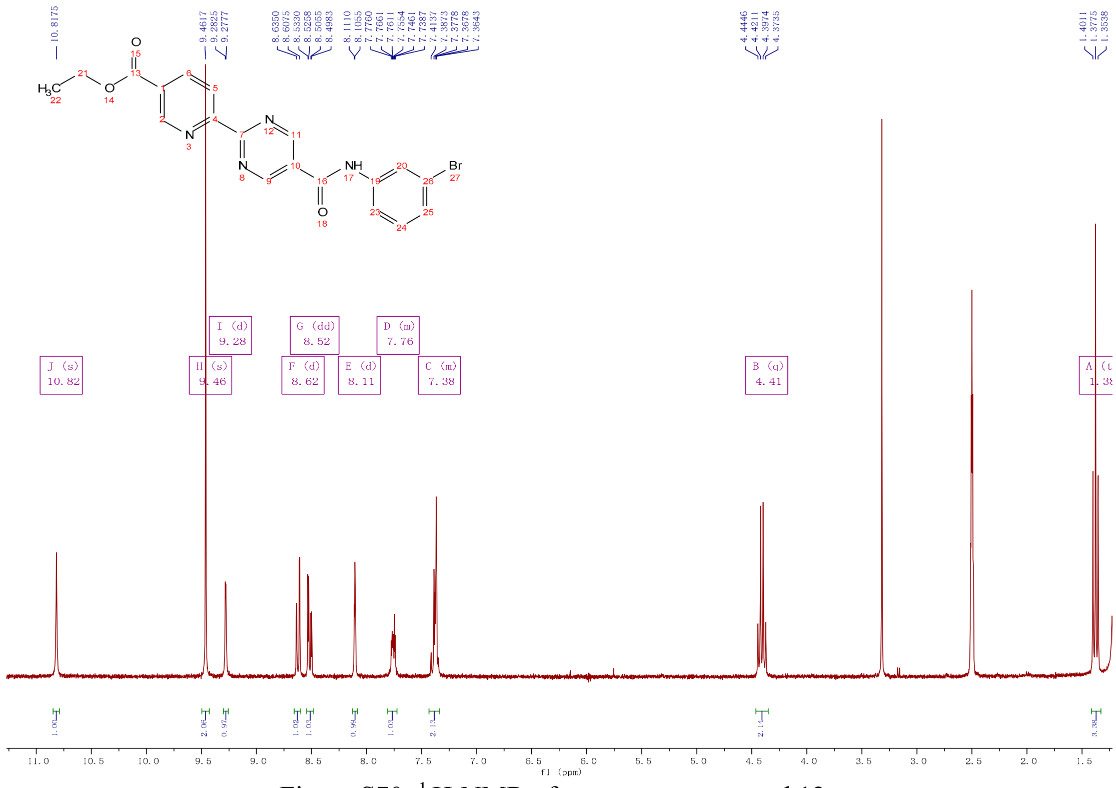

Figure S70: ^{1}H-NMR of spectrum compound 12n

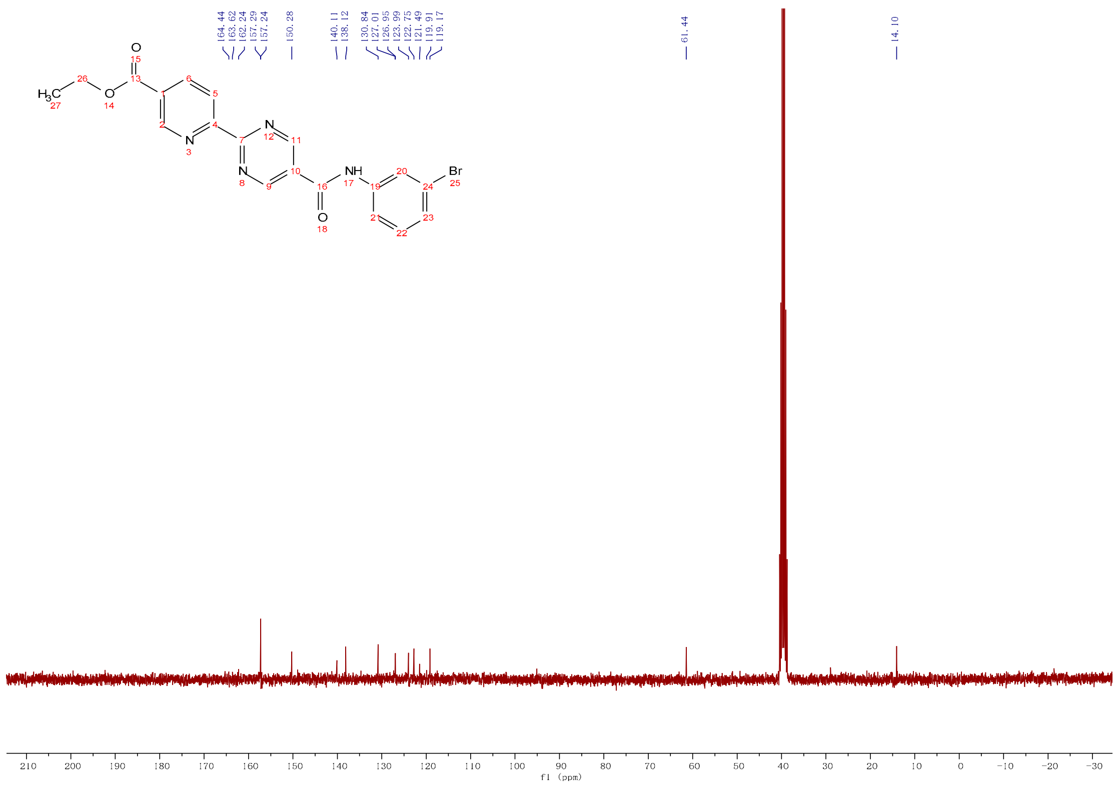

Figure S71: ^{13}C -NMR of spectrum compound 12n

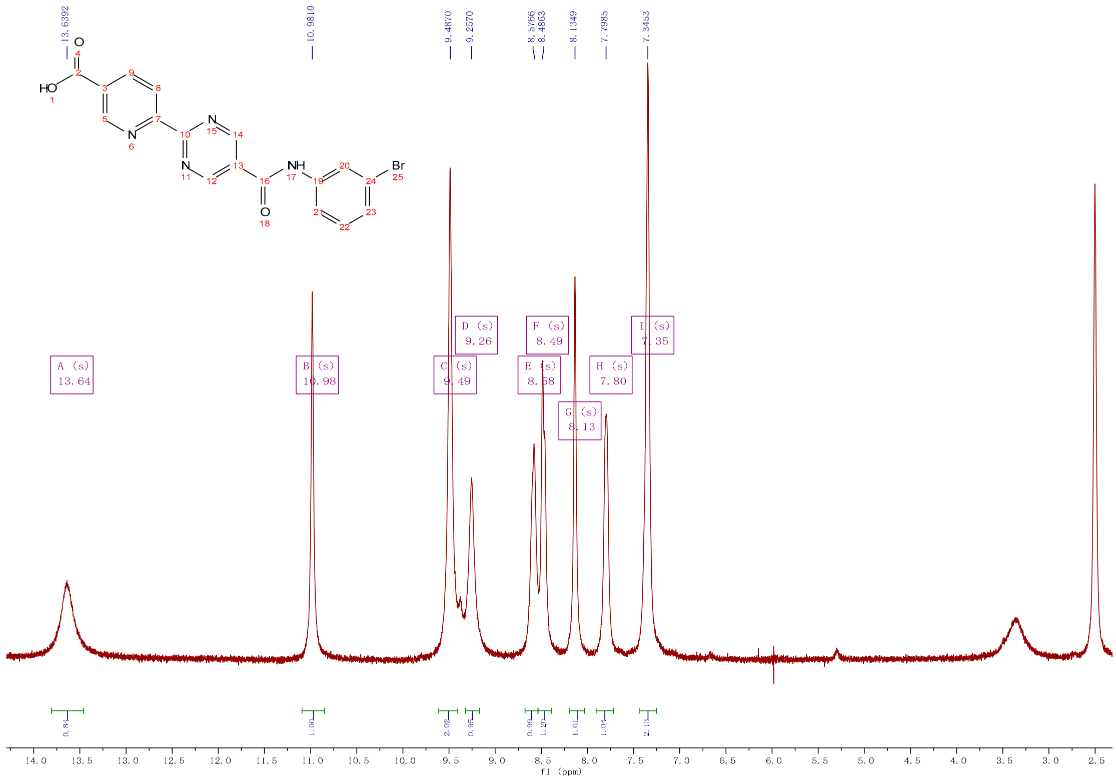

Figure S72: ^{1}H-NMR of spectrum compound 13n

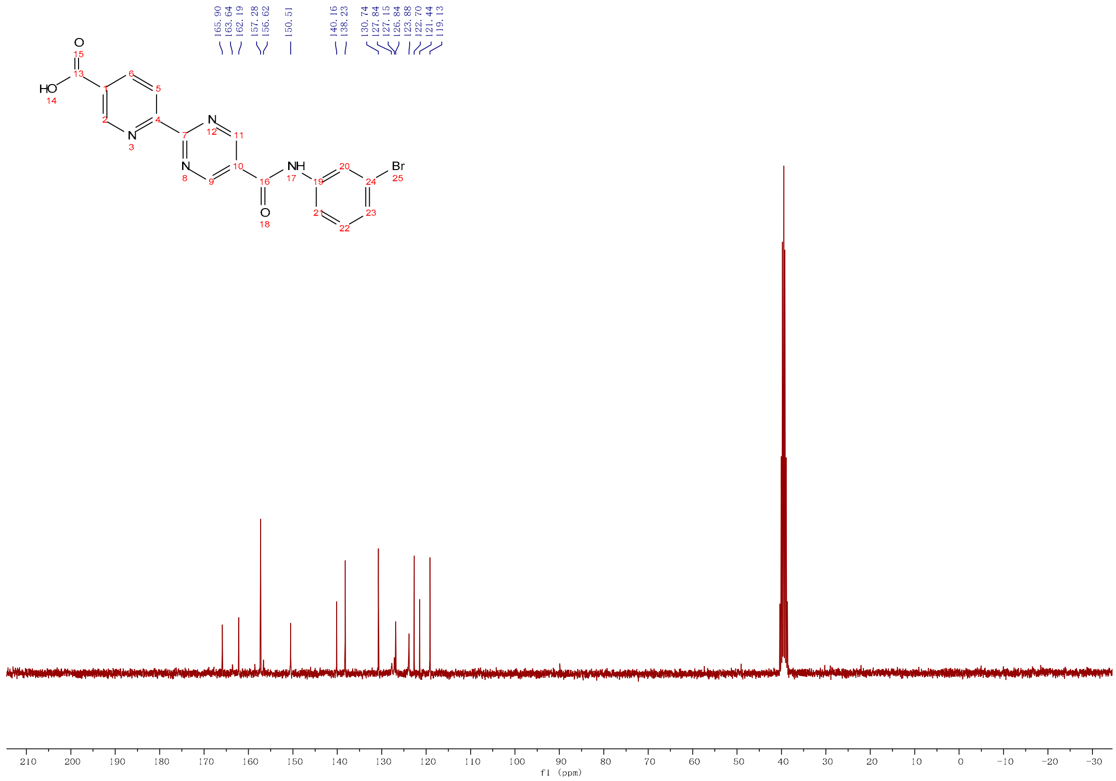

Figure S73: ^{13}C -NMR of spectrum compound 13n

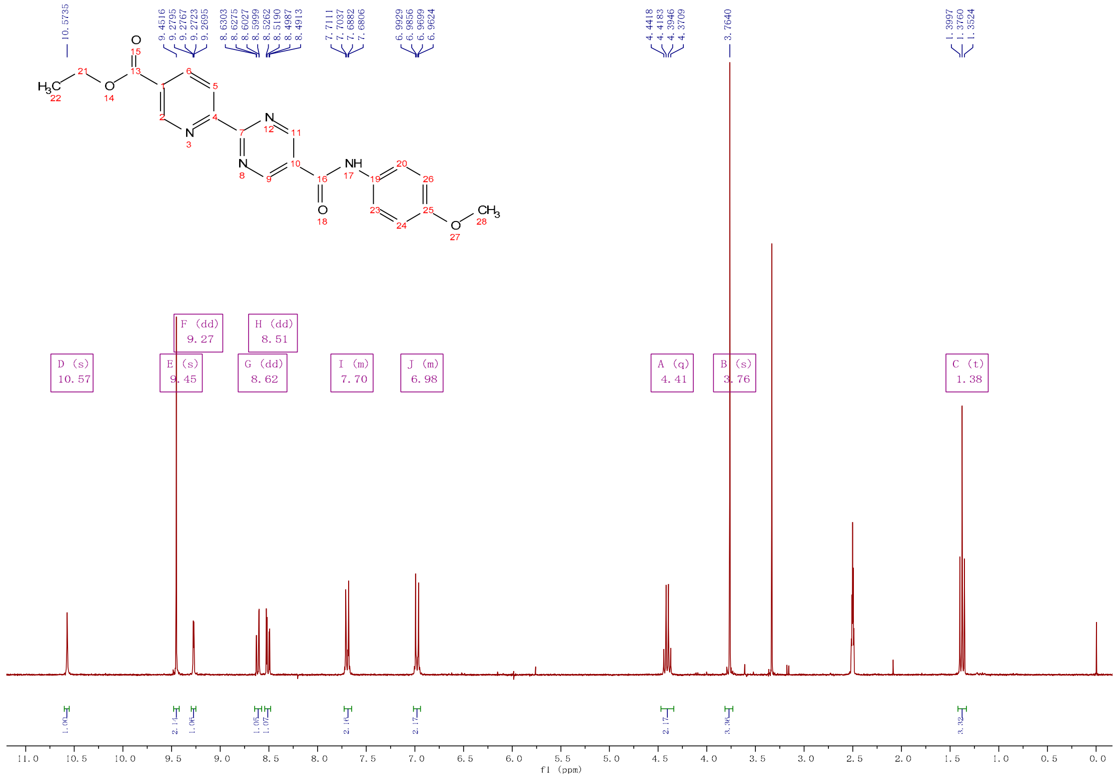

Figure S74: ^{1}H-NMR of spectrum compound 12o

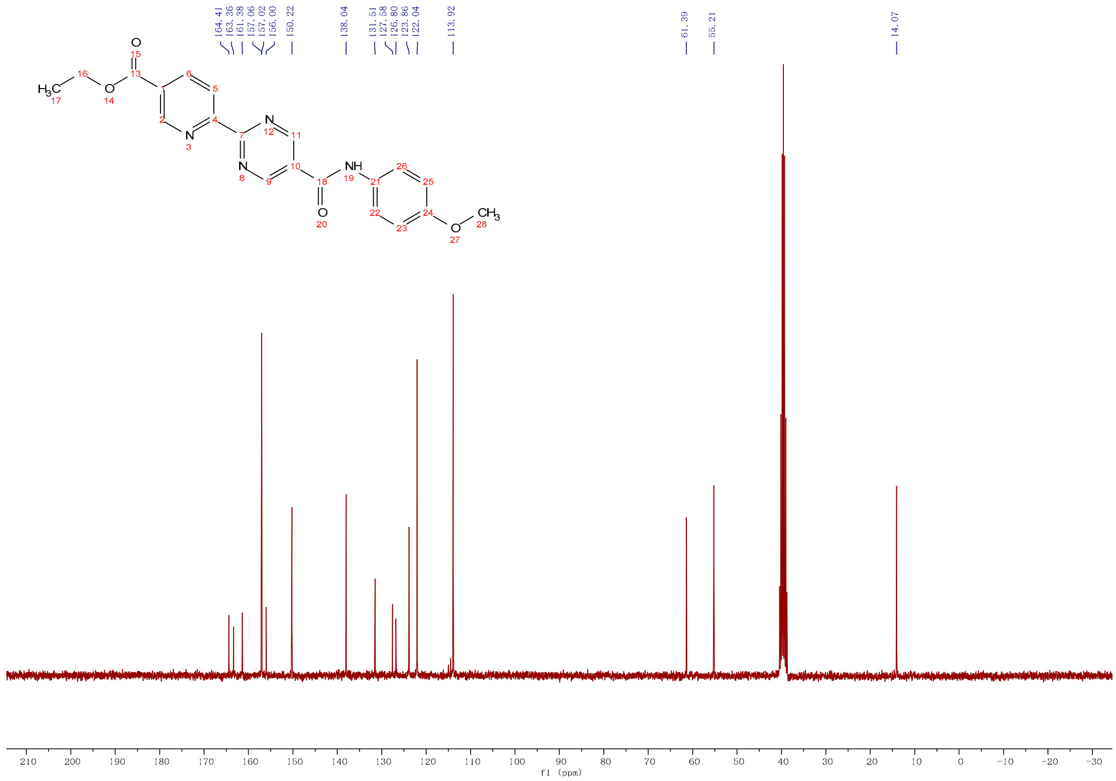

Figure S75: ^{13}C -NMR of spectrum compound 12o

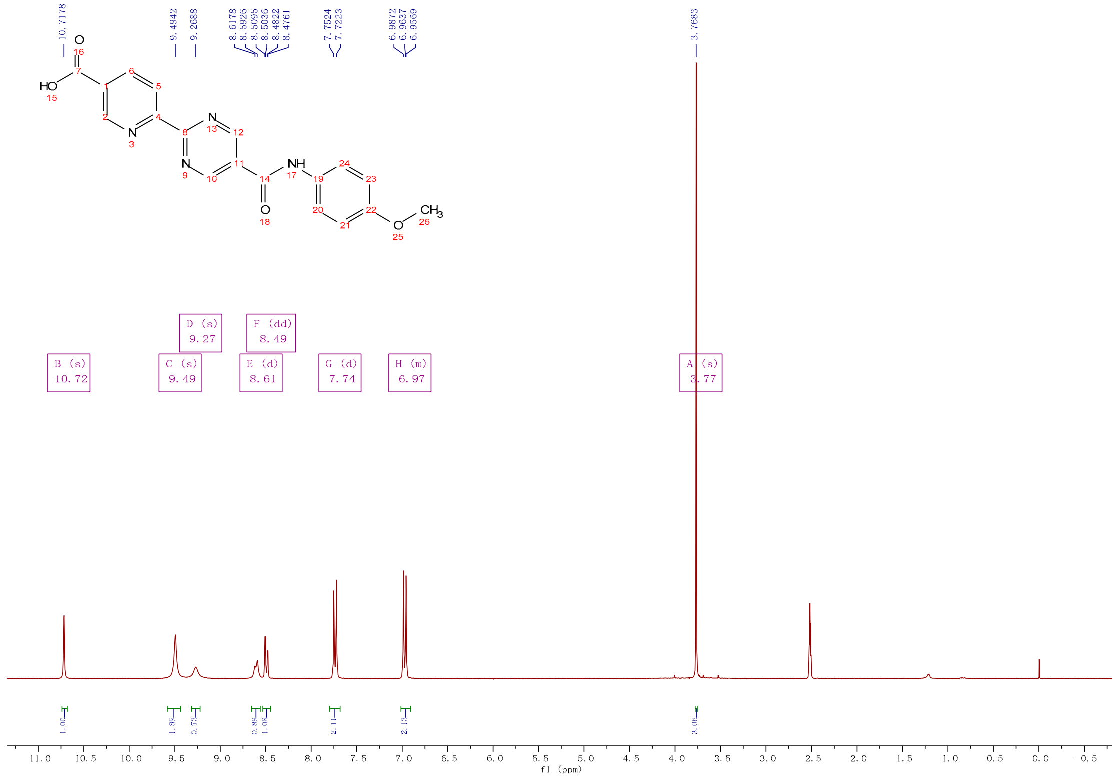

Figure S76: ^{1}H-NMR of spectrum compound 13o

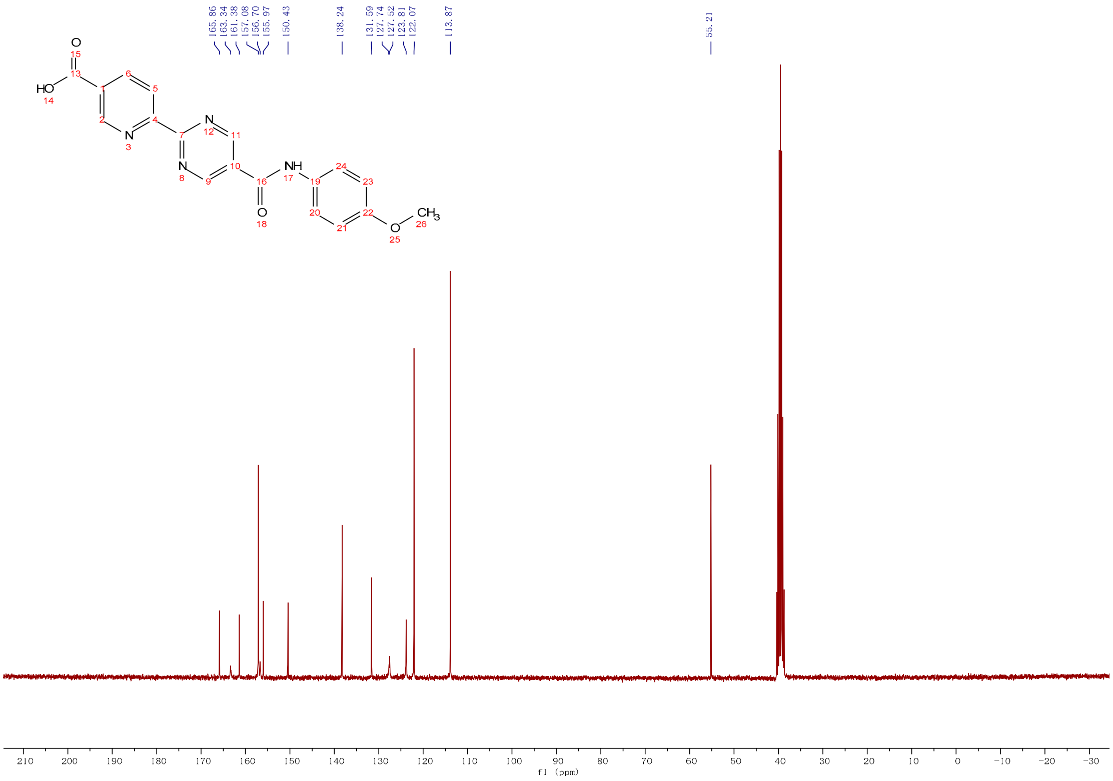

Figure S77: ^{13}C -NMR of spectrum compound 13o

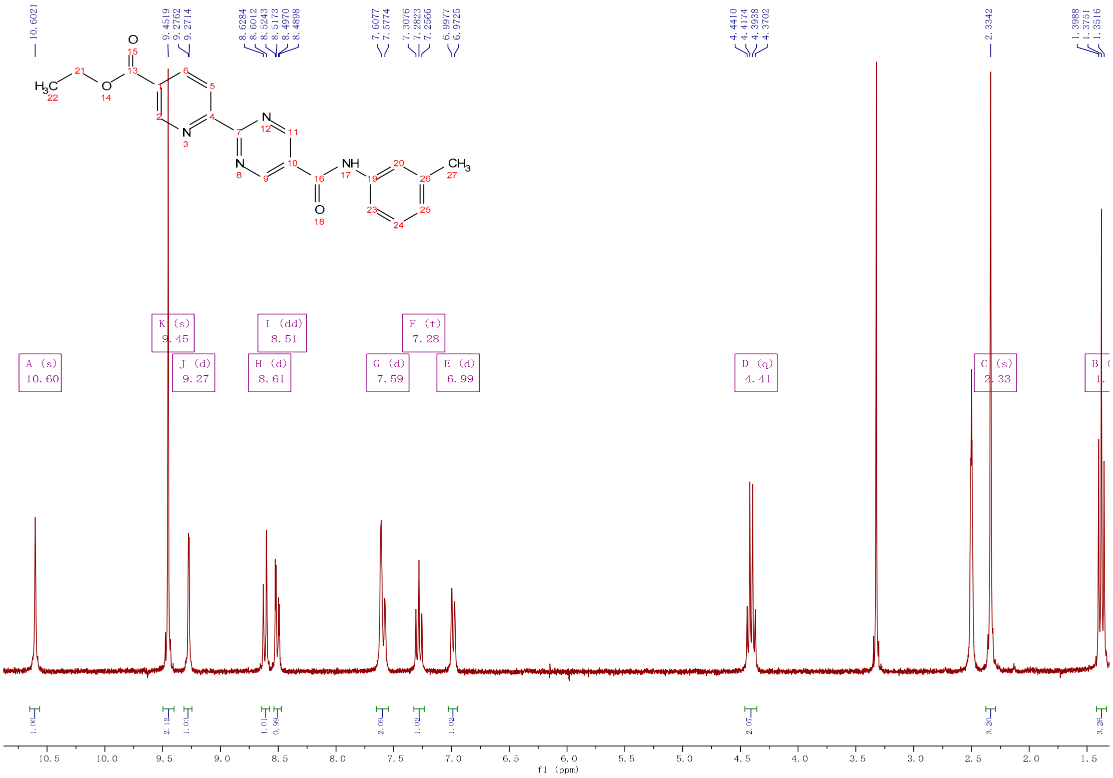

Figure S78: ^{1}H-NMR of spectrum compound 12p

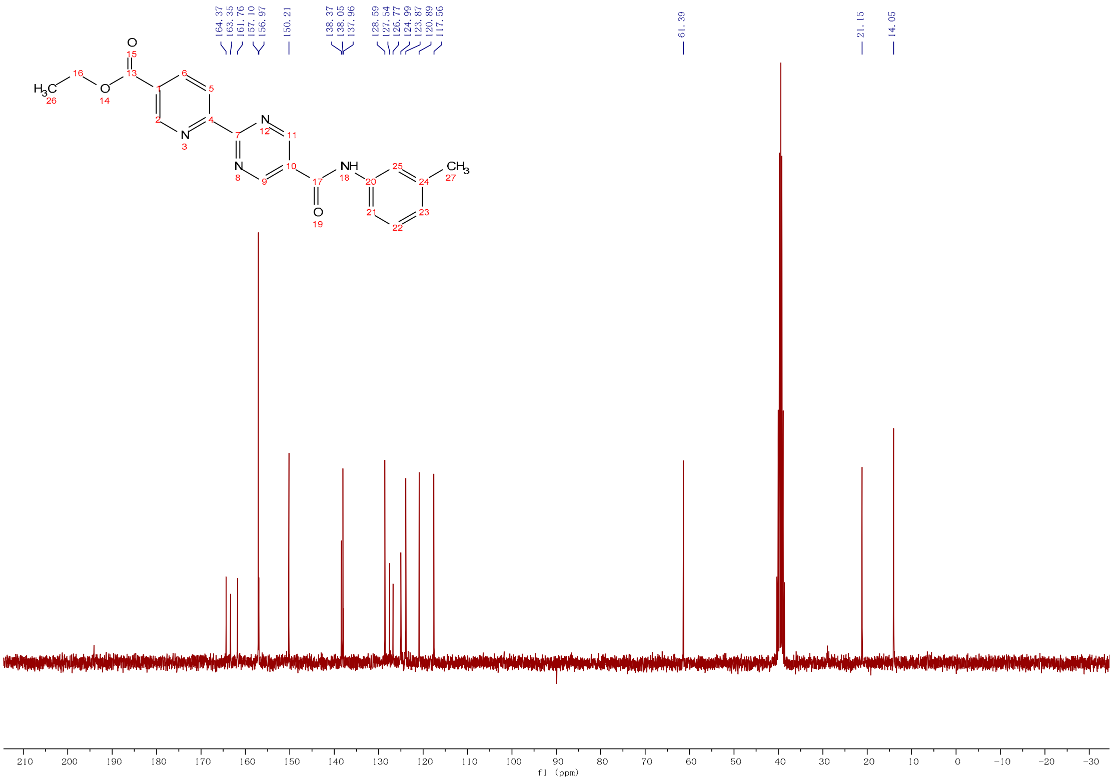

Figure S79: ^{13}C -NMR of spectrum compound 12p

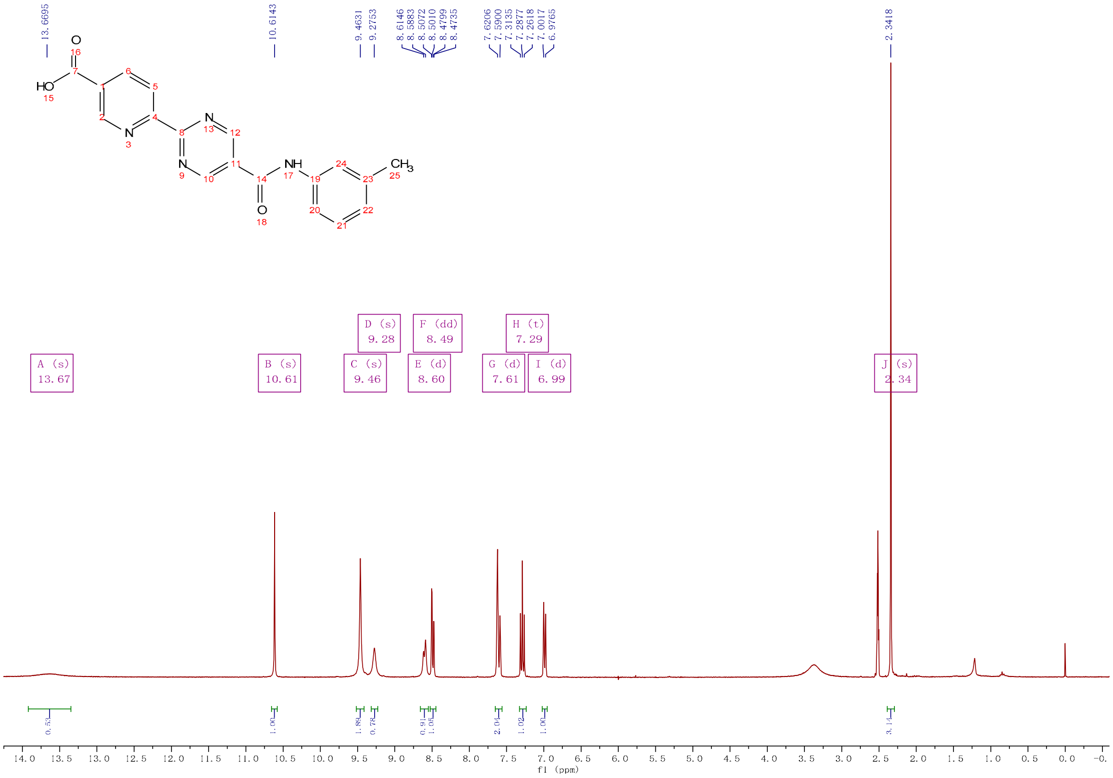

Figure S80: ^{1} H-NMR of spectrum compound 13p

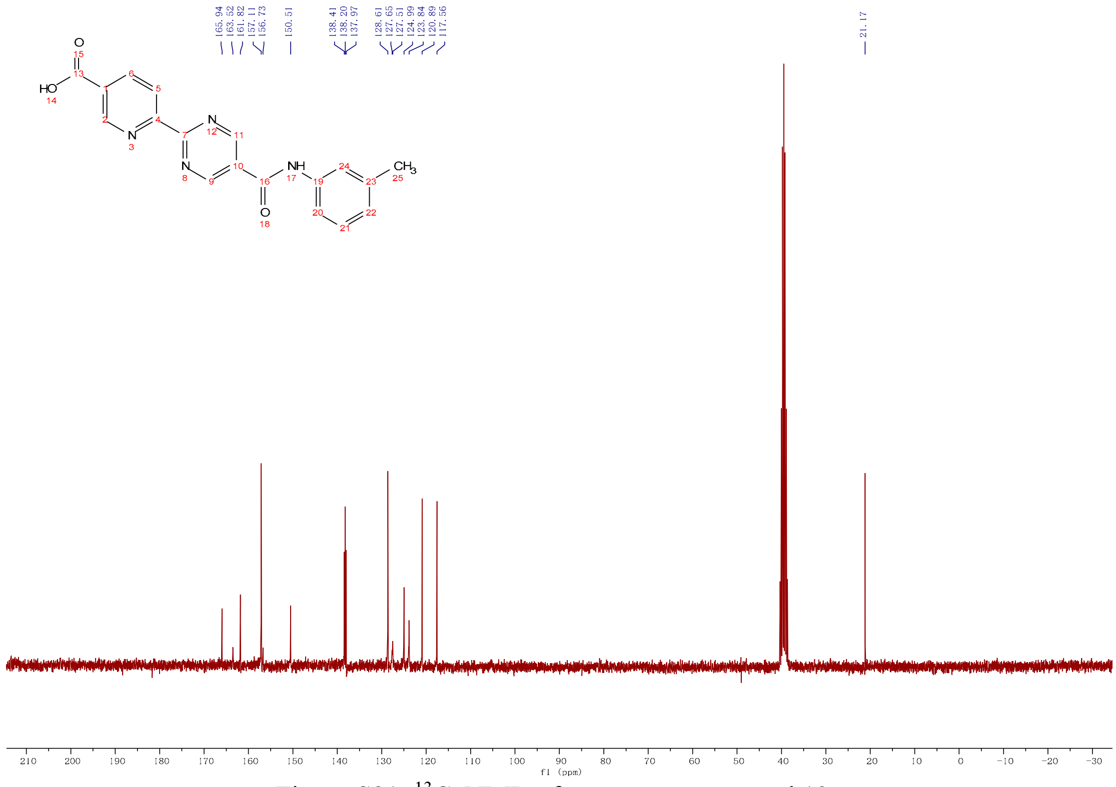

Figure S81: ^{13}C -NMR of spectrum compound 13p

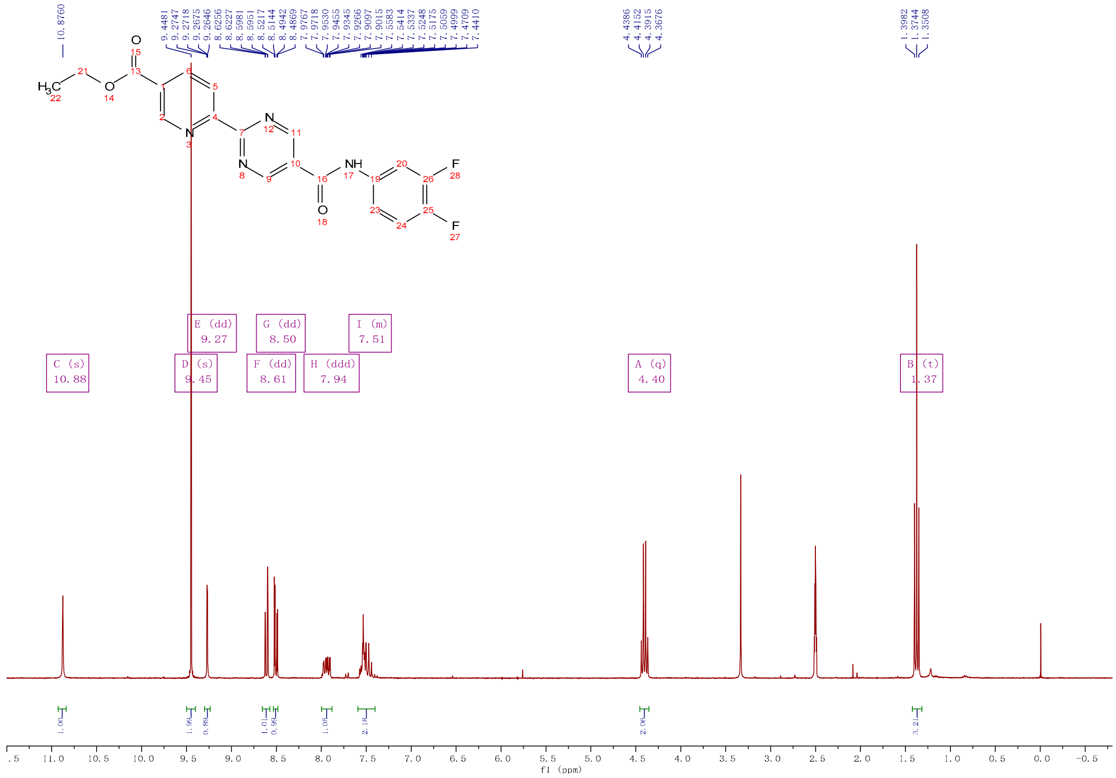

Figure S82: ^{1} H-NMR of spectrum compound 12q

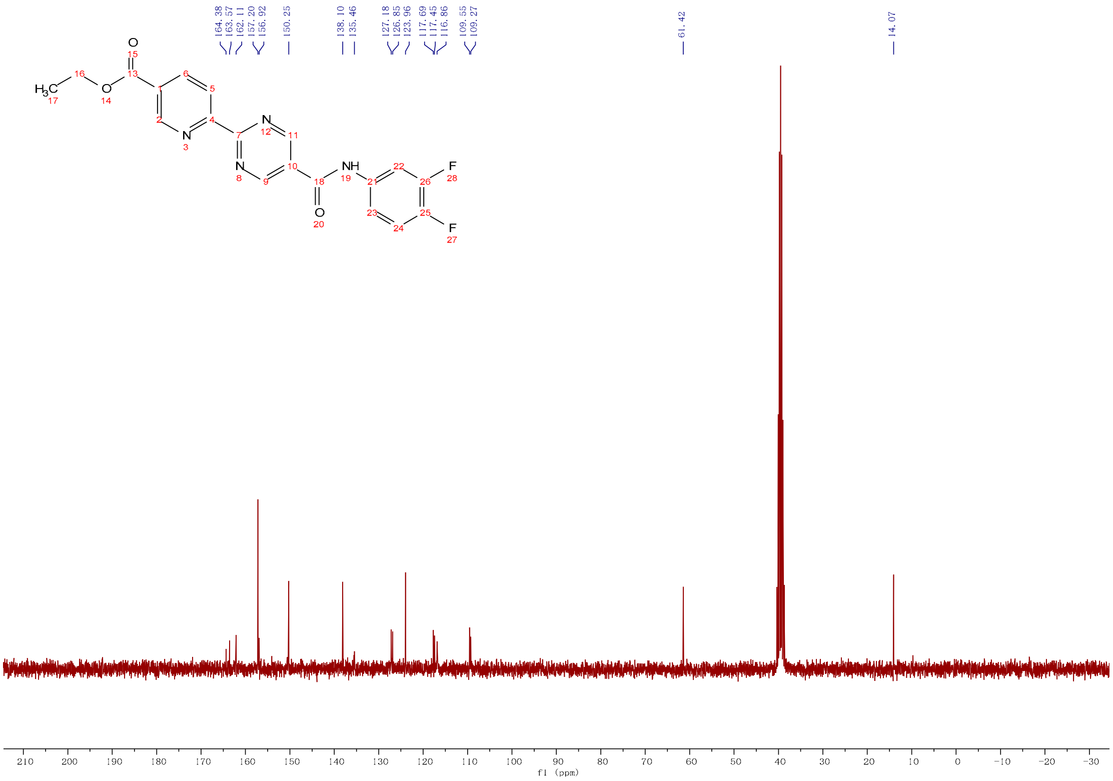

Figure S83: ^{13}C -NMR of spectrum compound 12q

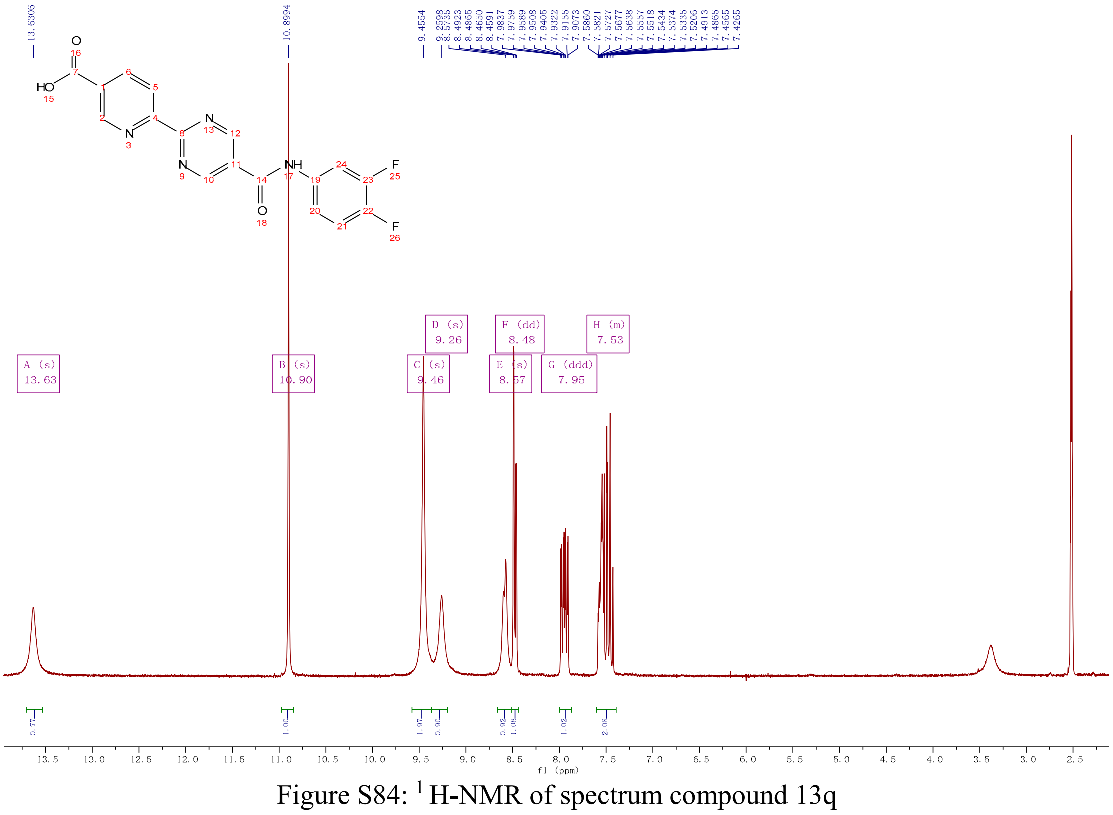

Figure S84: ^{1}H-NMR of spectrum compound 13q

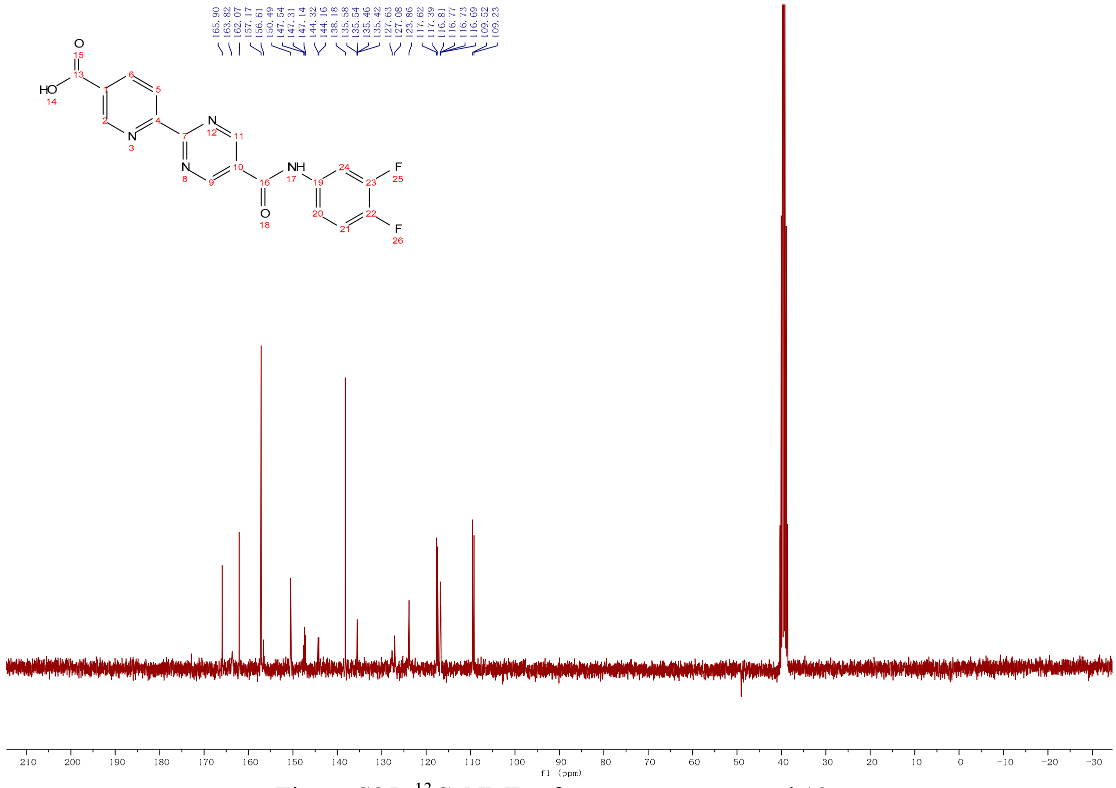

Figure S85: ^{13}C -NMR of spectrum compound 13q

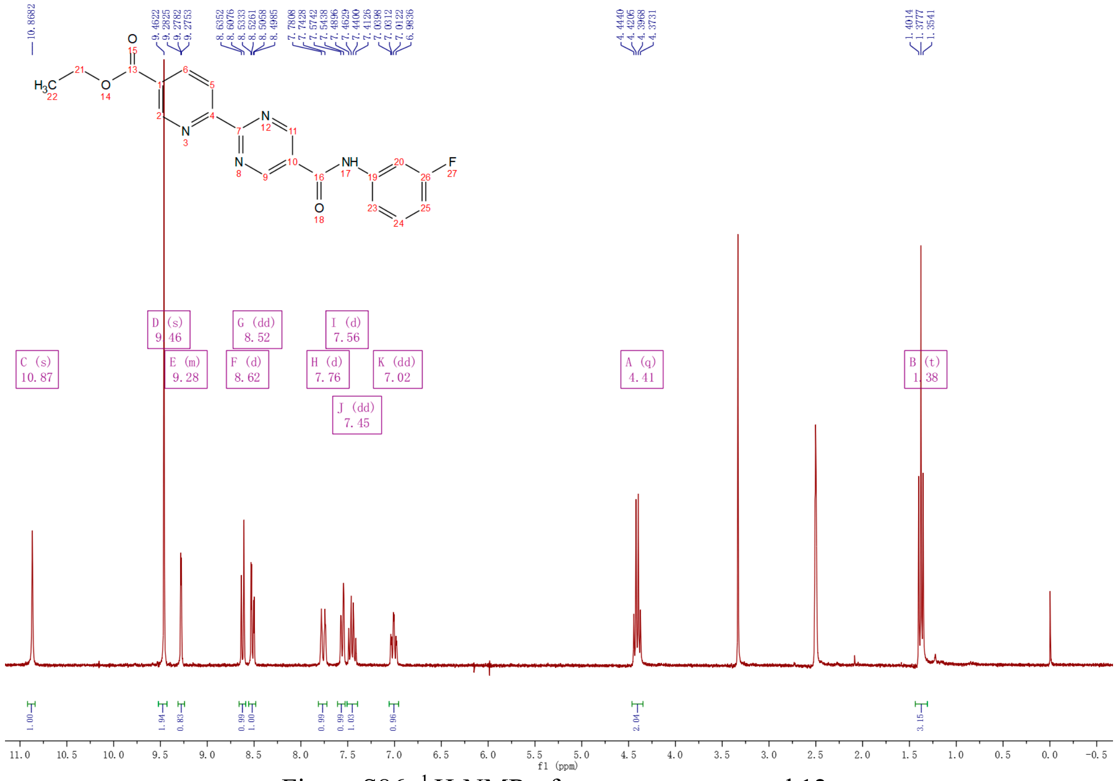

Figure S86: ^{1}H-NMR of spectrum compound 12r

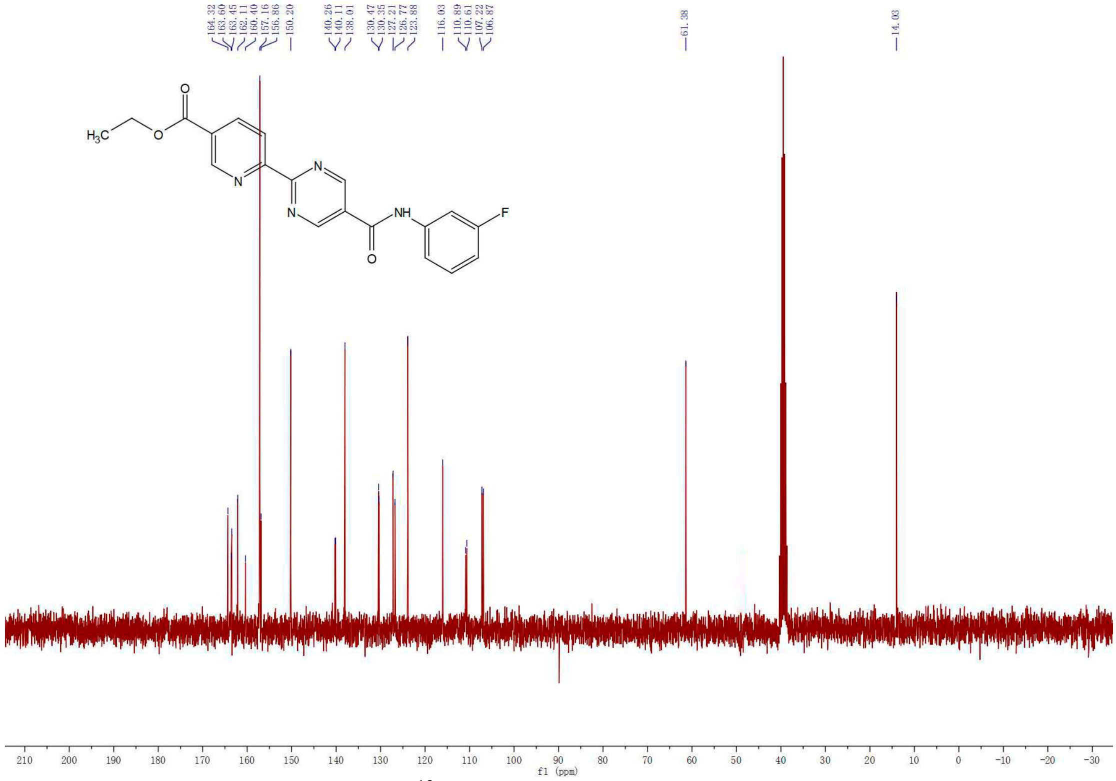

Figure S87: ^{13}C -NMR of spectrum compound 12r

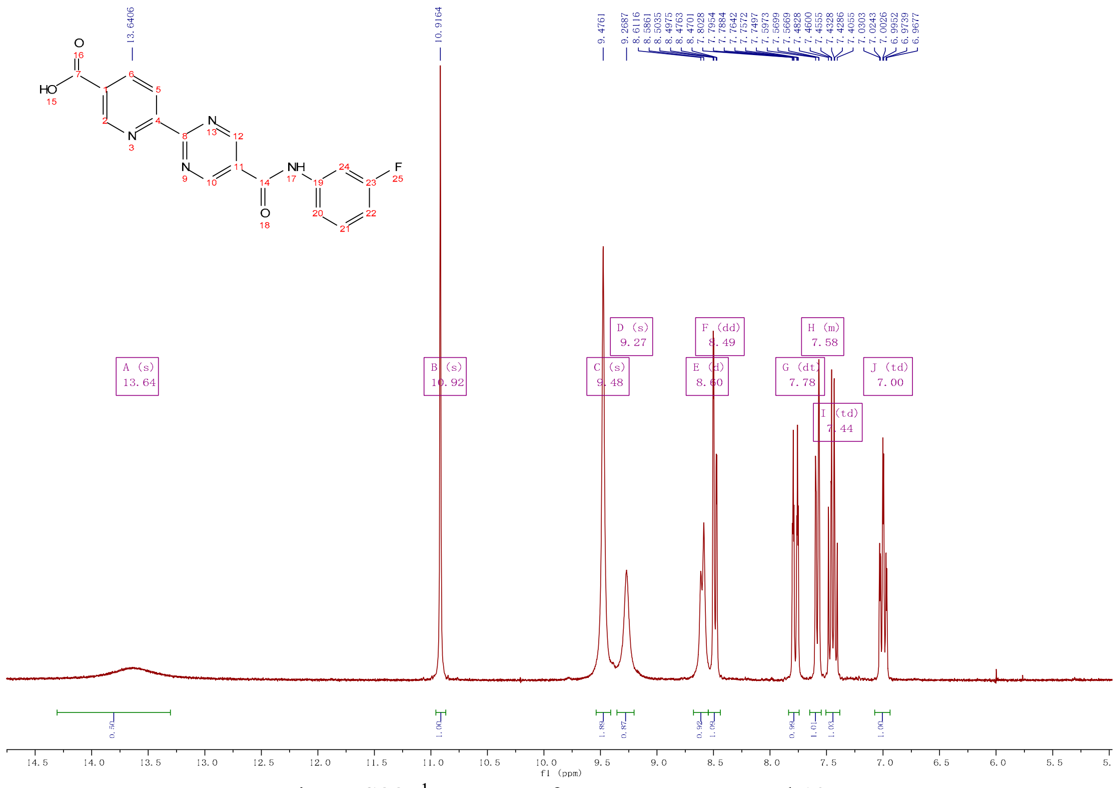

Figure S88: ^{1} H-NMR of spectrum compound 13r

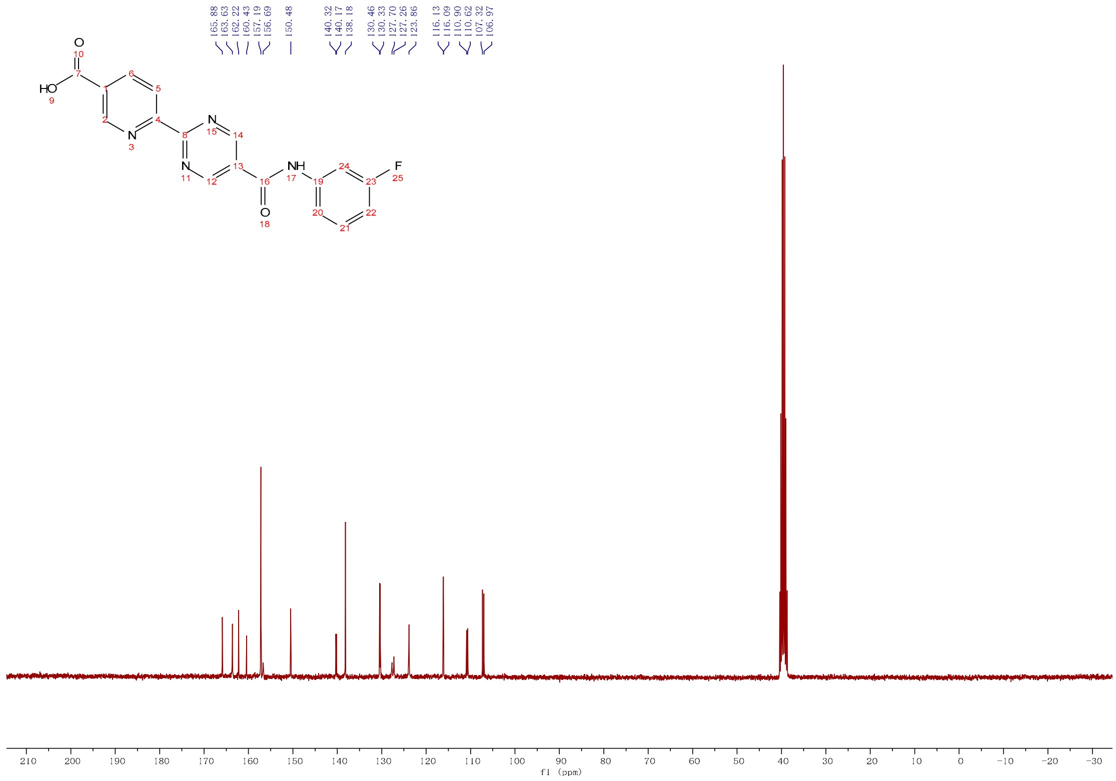

Figure S89: ^{13}C -NMR of spectrum compound 13r

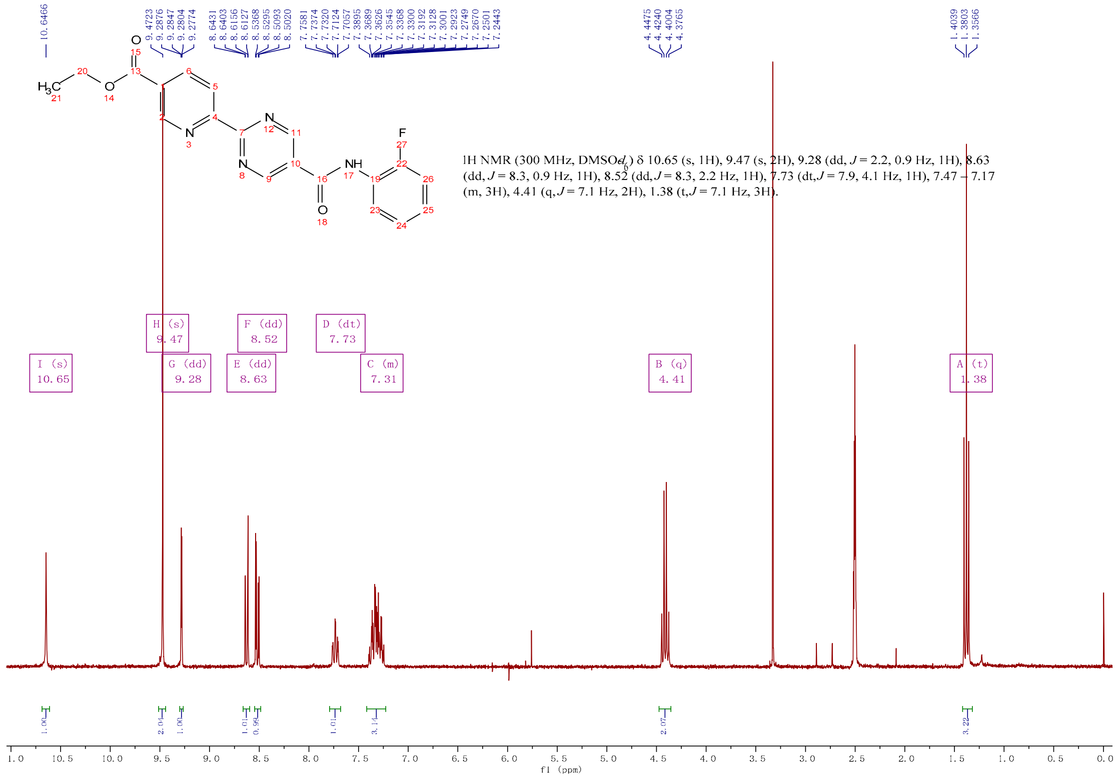

Figure S90: ^{1} H-NMR of spectrum compound 12s

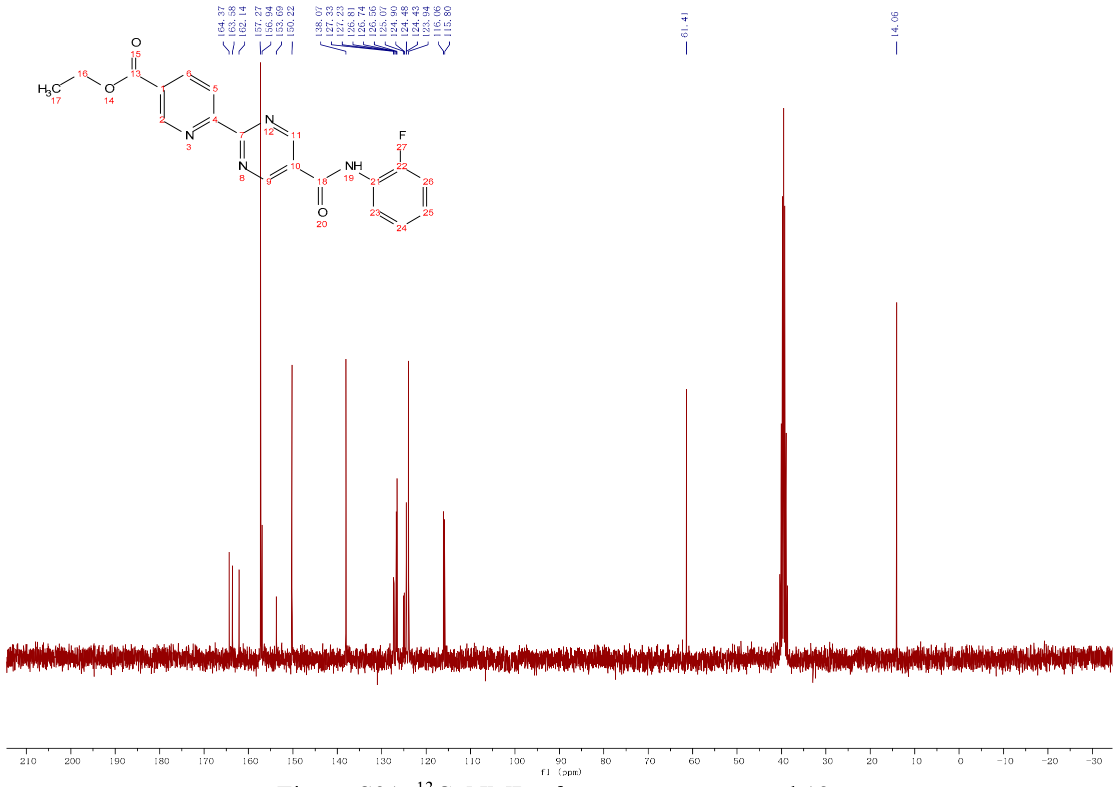

Figure S91: ^{13}C -NMR of spectrum compound 12s

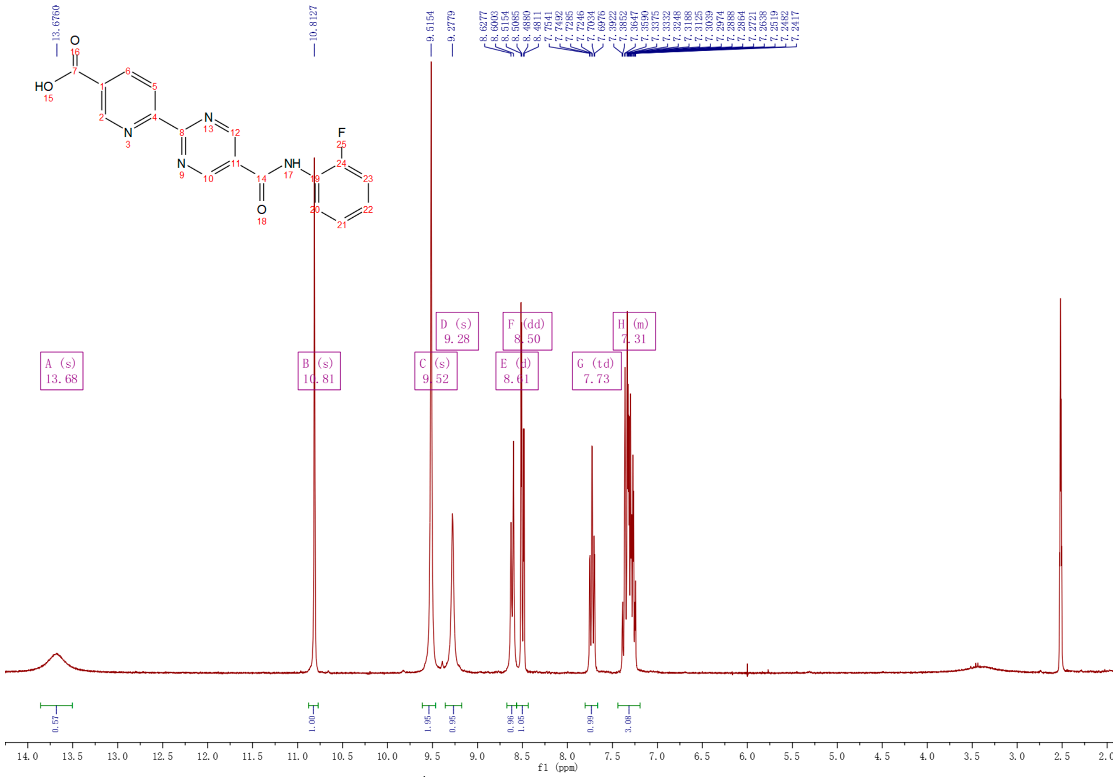

Figure S92: ^{1}H-NMR of spectrum compound 13s

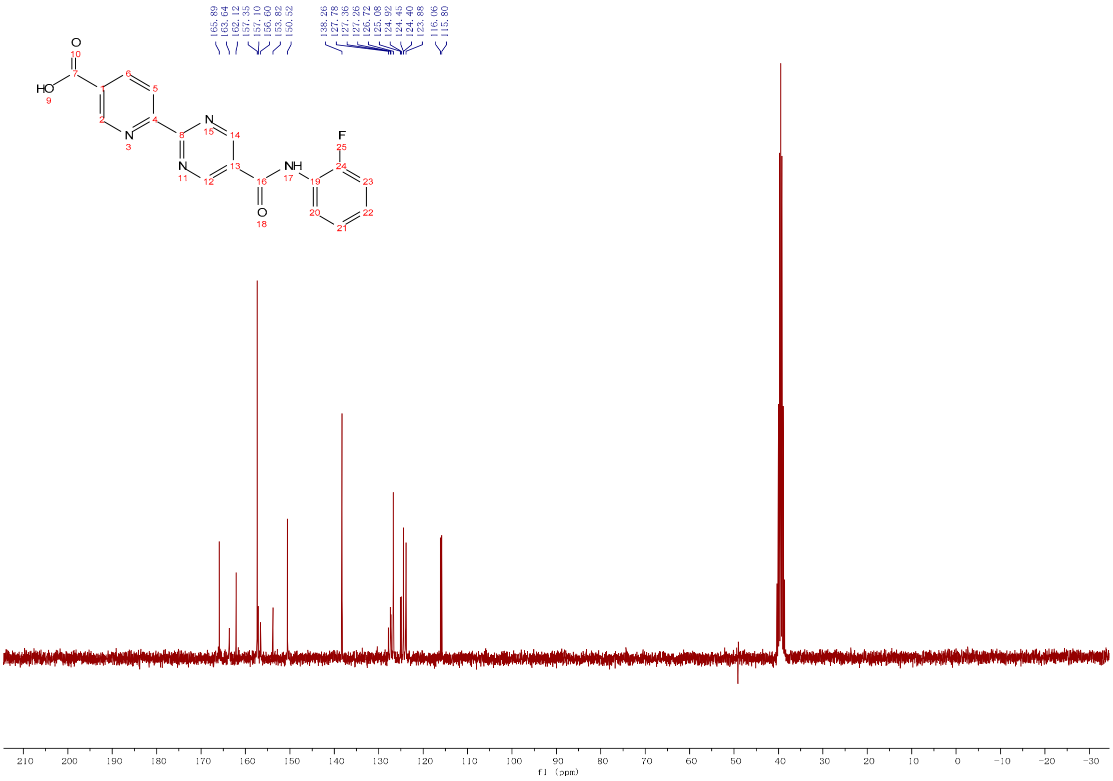

Figure S93: ^{13}C -NMR of spectrum compound 13s

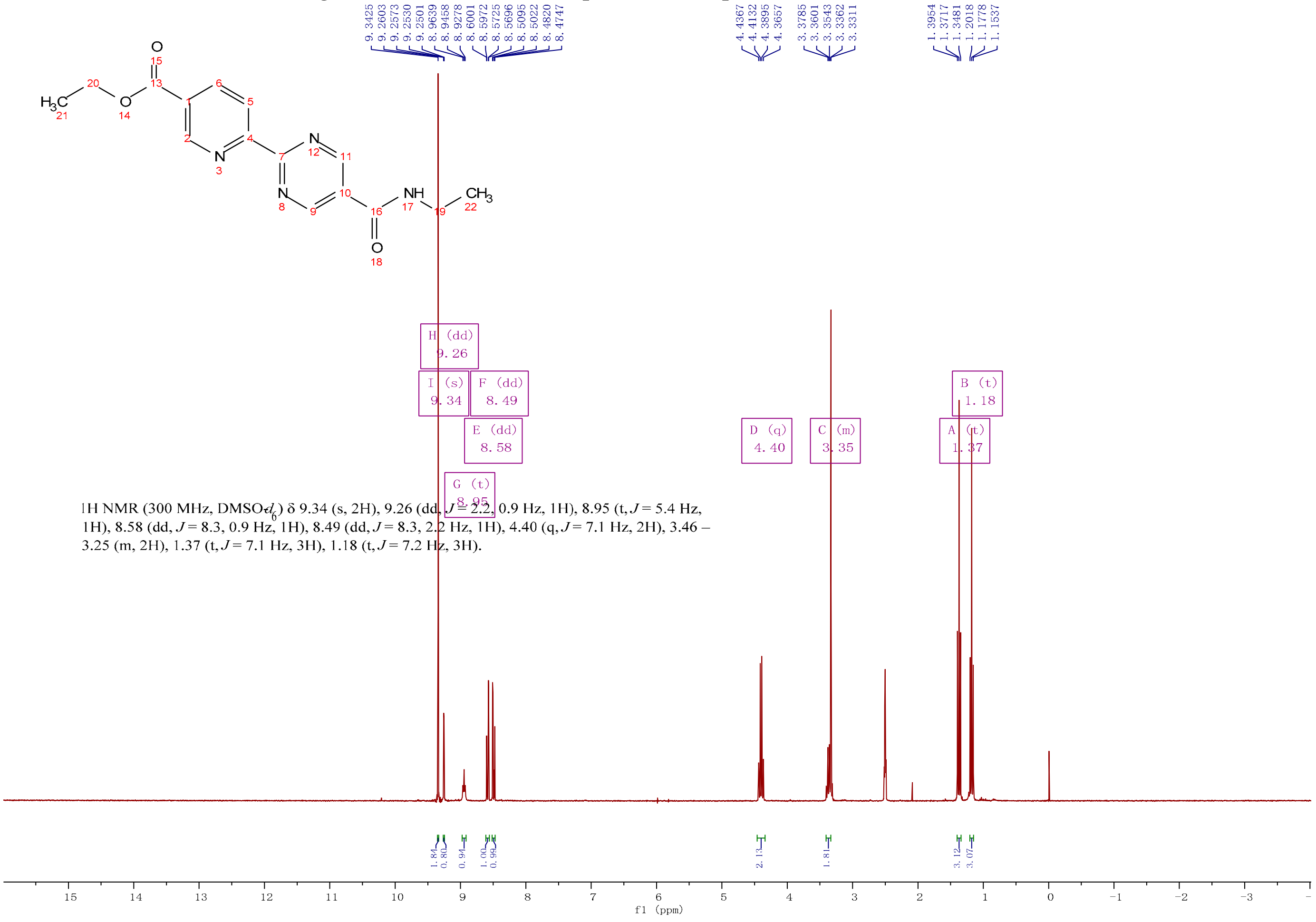

Figure S94: ^{1} H-NMR of spectrum compound 12t

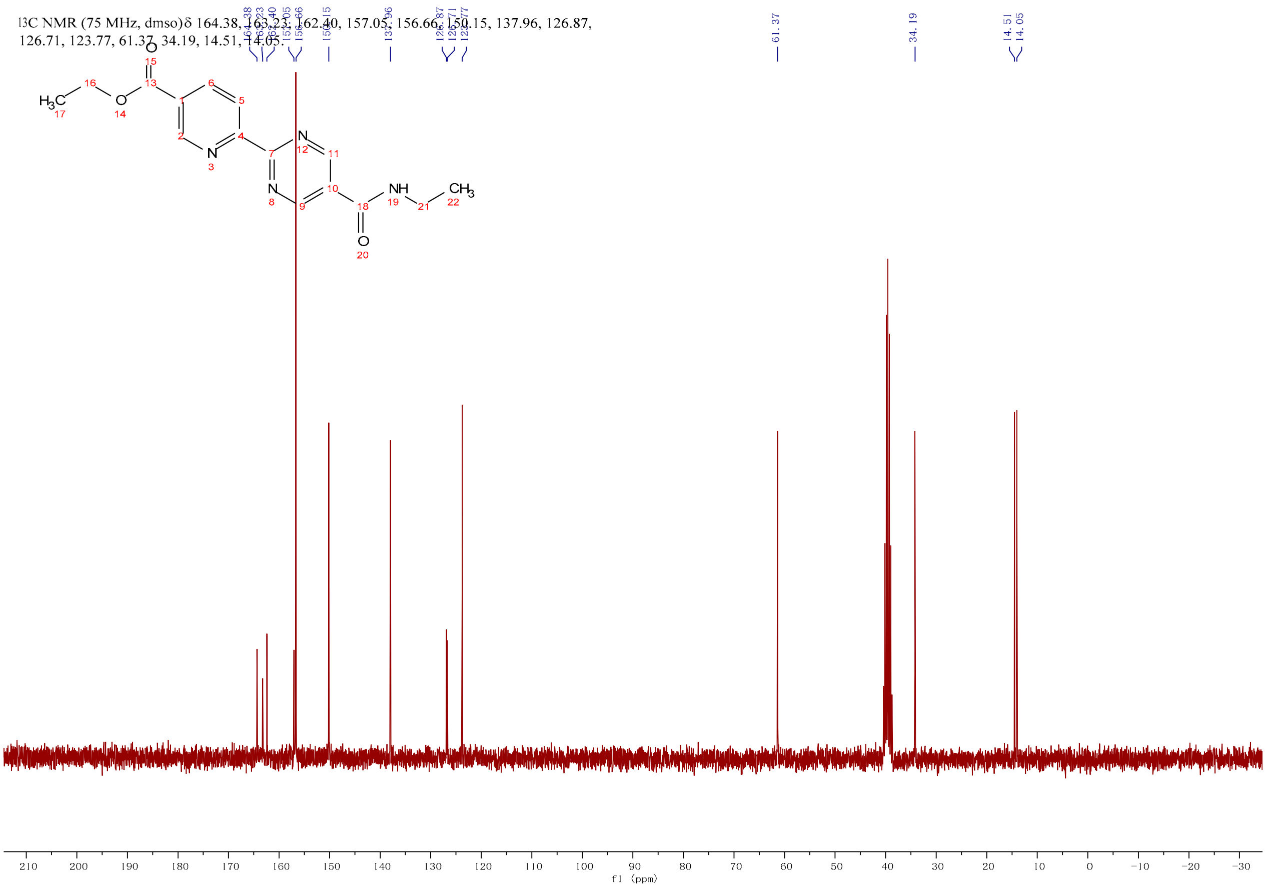

Figure S95: ^{13}C -NMR of spectrum compound 12t

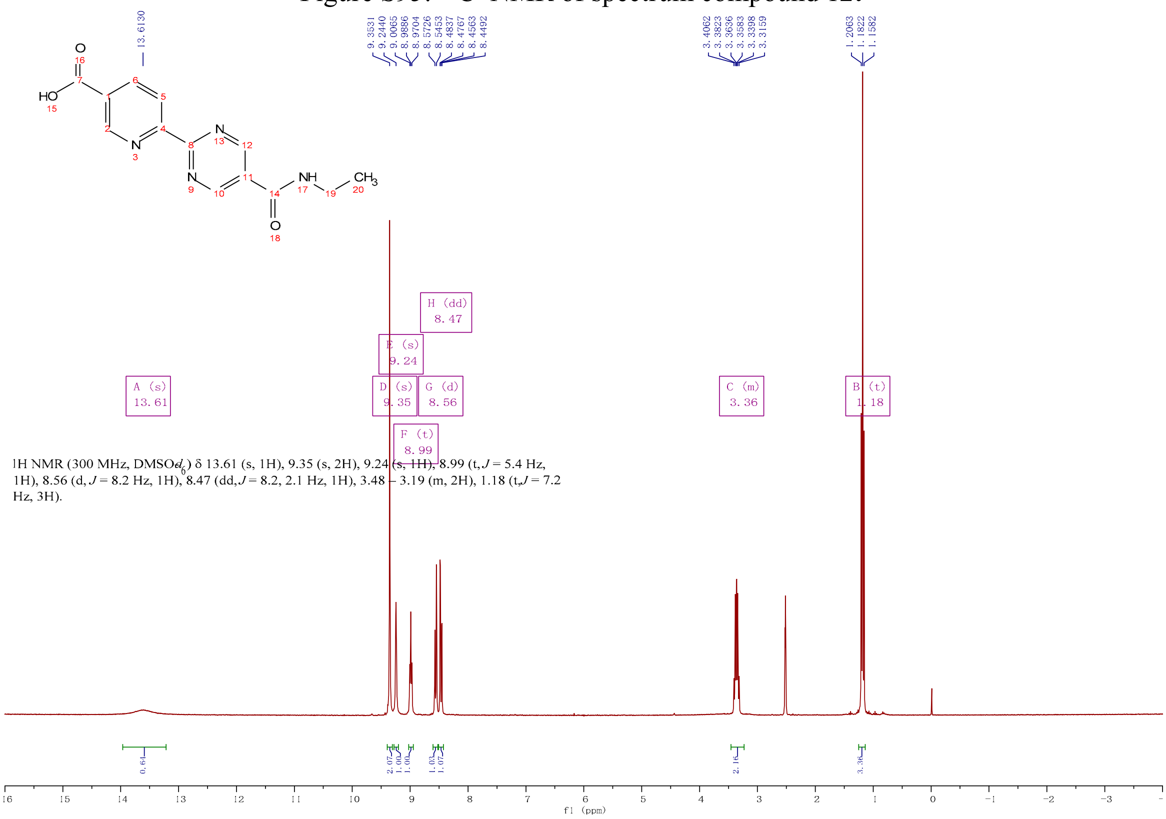

Figure S96: ^{1} H-NMR of spectrum compound 13t

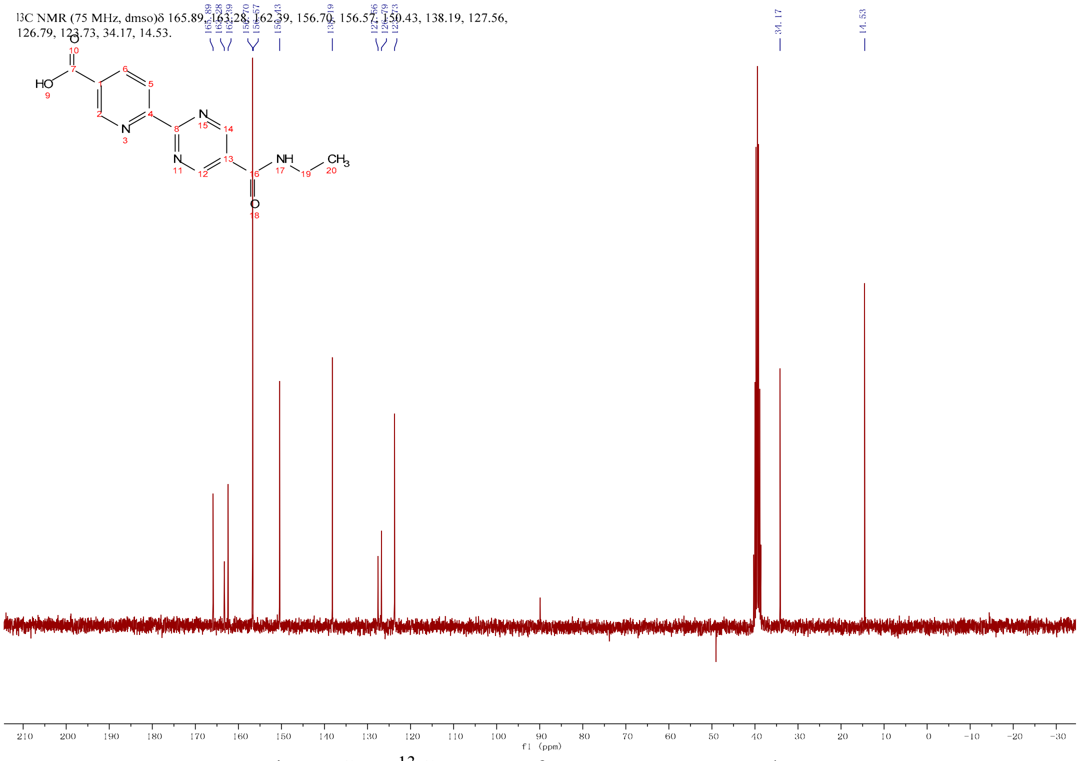

Figure S97: ^{13}C -NMR of spectrum compound 13t

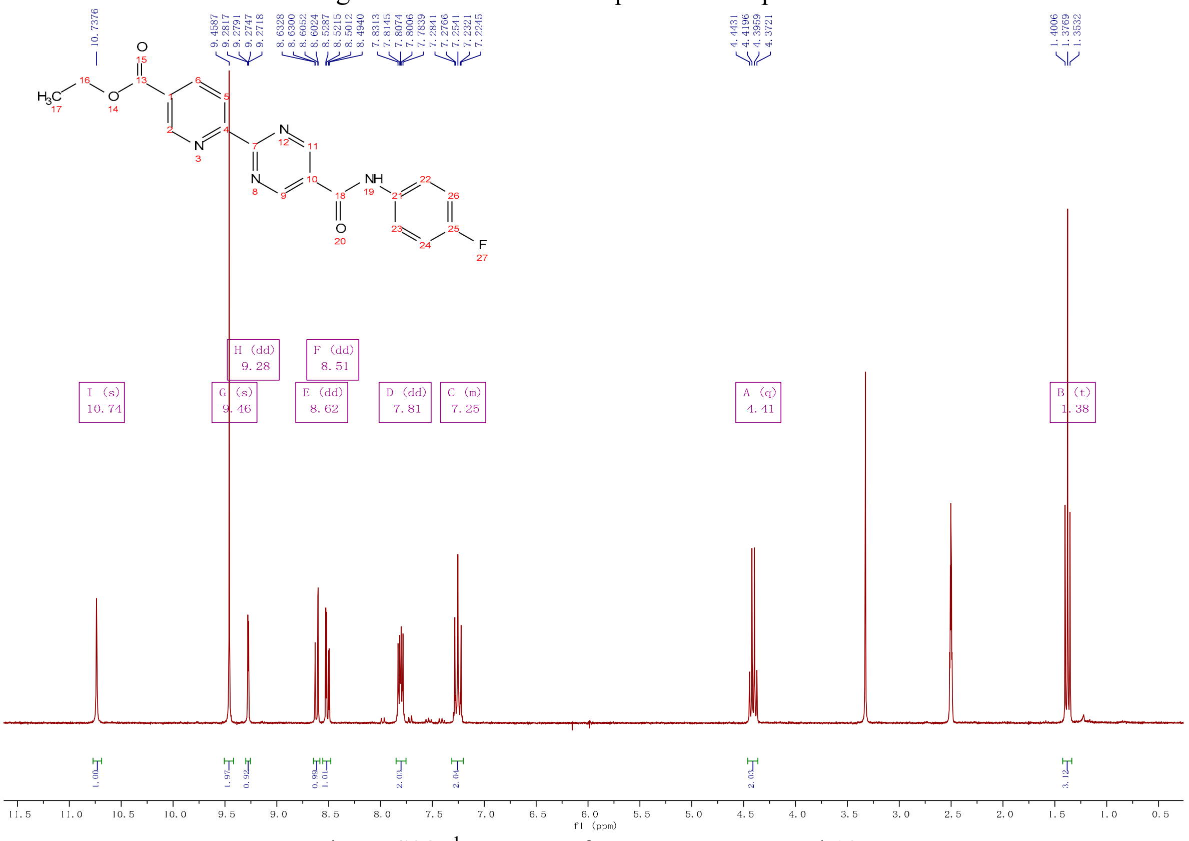

Figure S98: ^{1}H-NMR of spectrum compound 12u

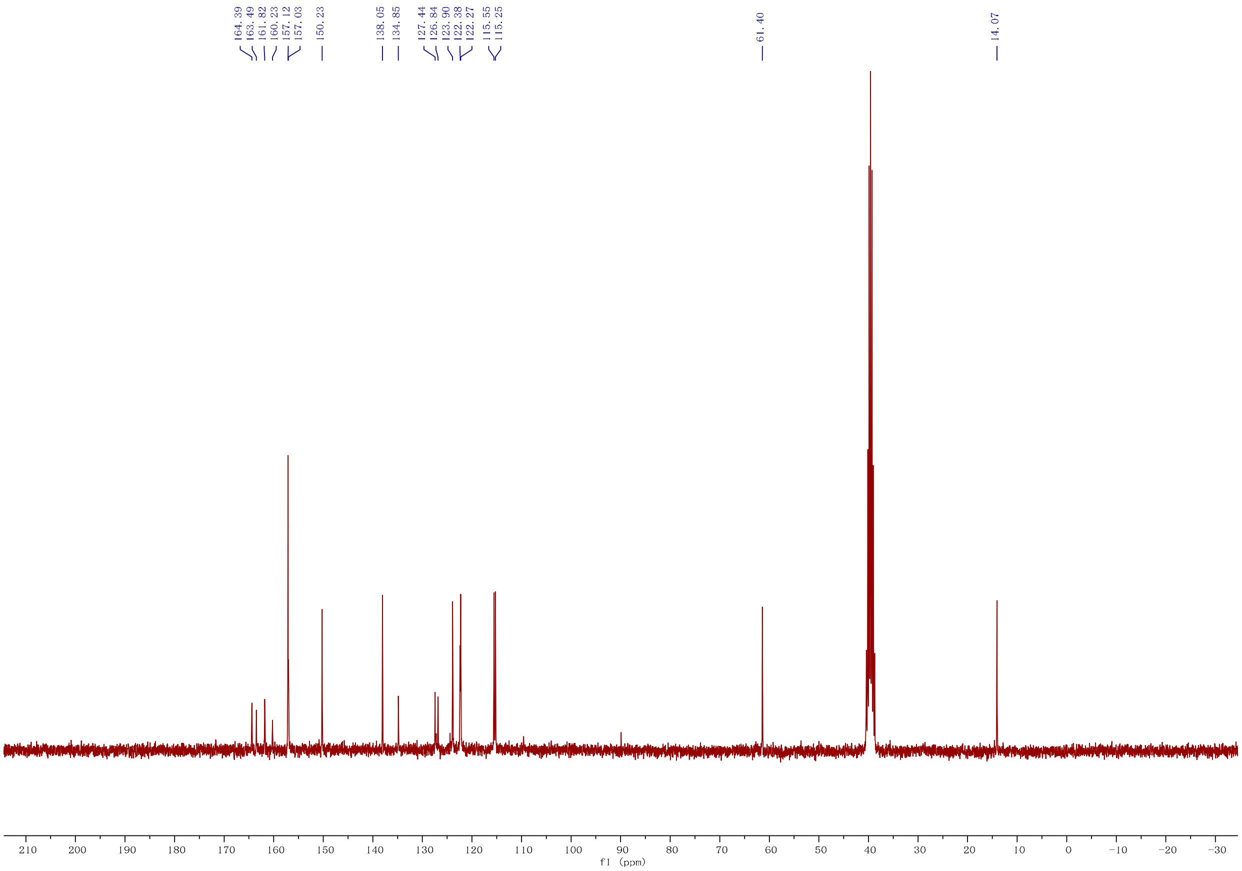

Figure S99: ^{13}C -NMR of spectrum compound 12u

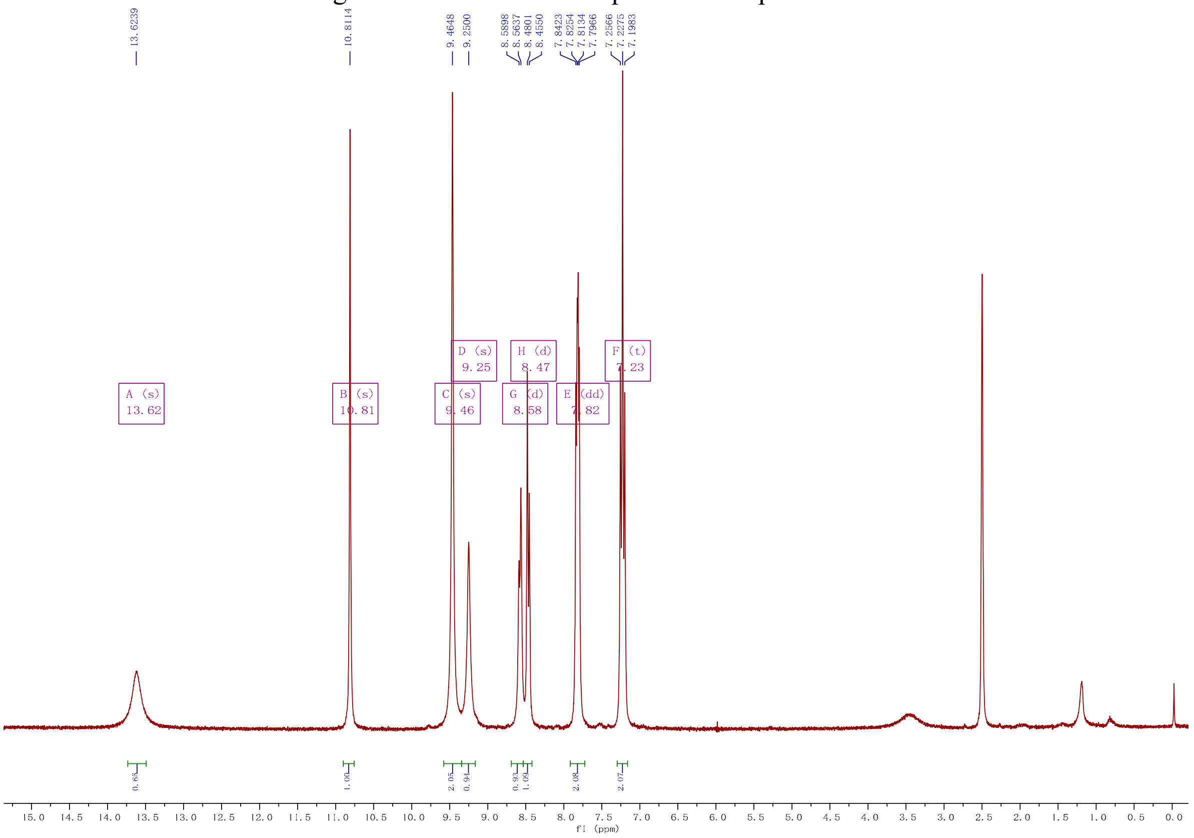

Figure S100: ^{1}H-NMR of spectrum compound 13u

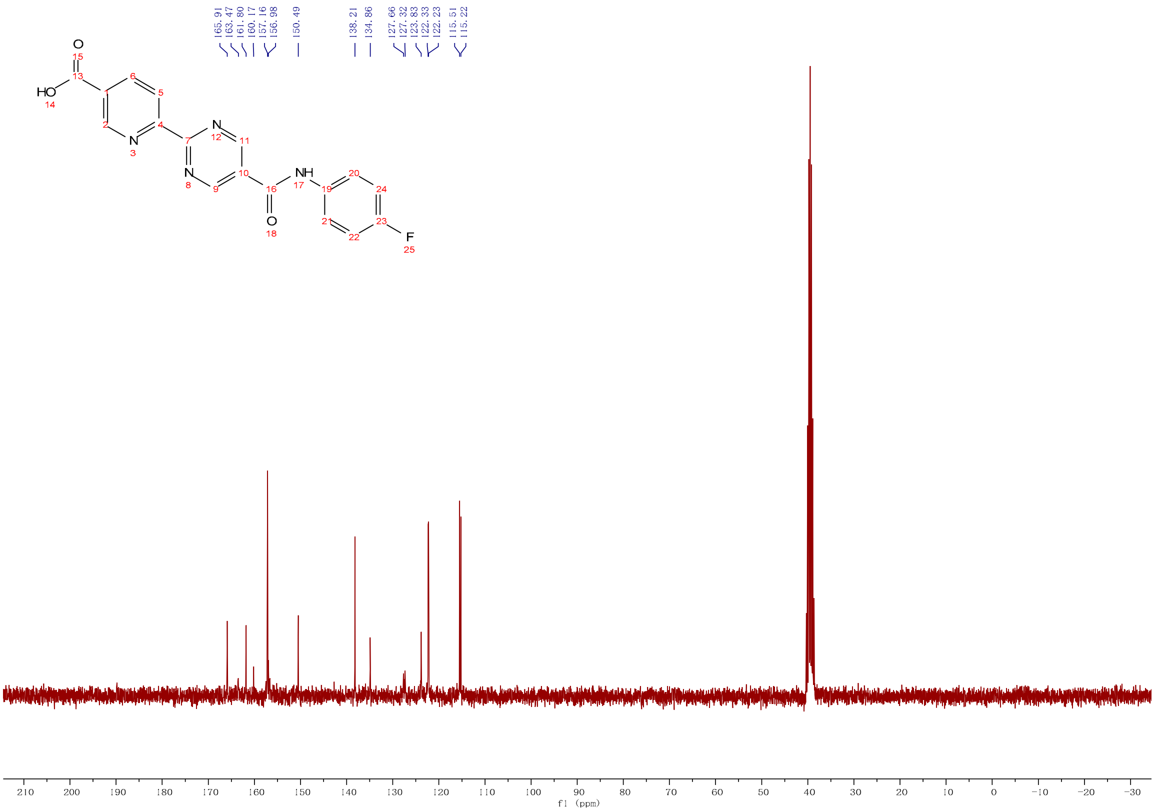

Figure S101: ^{13}C -NMR of spectrum compound 13u
